# Active layer microbial inocula restore missing functions across thawed permafrost soils

**DOI:** 10.1038/s41467-026-78060-4

**Published:** 2026-09-25

**Authors:** Sylvain Monteux, Ellen Dorrepaal, Sébastien Fontaine, Konstantin Gavazov, Sara Hallin, Jaanis Juhanson, Frida Keuper, Alexander T. Tveit, Josefine Walz, Rica Wegner, Birgit Wild, Eveline J. Krab

**Affiliations:** 1 Department of Soil and Environment, Swedish University of Agricultural Sciences, Uppsala, Sweden; 2 Department of Environmental Science, Stockholm University, Stockholm, Sweden; 3 Bolin Centre for Climate Research, Stockholm University, Stockholm, Sweden; 4 Tromsø Museum, UiT The Arctic University of Norway, Tromsø, Norway; 5 Climate Impacts Research Centre, Umeå University, Abisko, Sweden; 6 Grassland Ecosystem Research Unit, French National Institute for Food, Agriculture and Environment INRAE, Clermont-Ferrand, France; 7 Swiss Federal Institute for Forest, Snow and Landscape Research WSL, Birmensdorf, Switzerland; 8 Department of Forest Mycology and Plant Pathology, Swedish University of Agricultural Sciences, Uppsala, Sweden; 9 Science for Life Laboratory, Swedish University of Agricultural Sciences, Uppsala, Sweden; 10 BioEcoAgro Joint Cross-Border Research Unit, French National Institute for Food, Agriculture and Environment INRAE, Laon, France; 11 Department of Arctic and Marine Biology, UiT The Arctic University of Norway, Tromsø, Norway

**Keywords:** Climate-change ecology, Microbial ecology, Cryospheric science

## Abstract

Microbial dynamics in thawing permafrost induce a climate feedback of uncertain magnitude. Upon thaw, active layer microorganisms may establish in formerly frozen layers and introduce missing functions. Here we test the prevalence of functional limitations and how active layer microorganisms can mitigate these across four widespread permafrost types over 389 days. By restoring functions with a multifunctional microbial community, we demonstrate widespread functional limitations, as increased CO_2_ emissions across four permafrost types, and onset N_2_O emissions in two of those. However, realistic active layer inocula increased CO_2_ or N_2_O production less than the multifunctional community, due to unsuccessful coalescence or limitations in the inocula. Under anoxia, active layer inocula quintupled CH_4_ while decreasing CO_2_ production, resulting in a 35% increase in CO_2_-eq over six months. Understanding the functional limitations of permafrost microbial communities will be key to predicting their substantial, yet unaccounted, consequences on the biogeochemistry of the permafrost region.

## Abstract (web summary)

After thawing, the introduction of top-soil microorganisms into different permafrost soils can restore missing functions. This increases the production of greenhouse gases, potentially altering the Arctic greenhouse gas budget.

## Introduction

Arctic permafrost soils store large amounts of carbon (C) that can fuel greenhouse gas (GHG) emissions when these soils thaw due to climate change^1,2^. Understanding the feedback between global warming and GHG emissions from these soils is important for climate change mitigation strategies^2–4^. Research efforts have been dedicated to sampling both the perennially frozen permafrost and the seasonally thawing active layer of soil profiles across the Arctic, to relate soil physical and chemical composition to long-term GHG production through in vitro incubation studies^1,2,5,6^. Incubations provide valuable data and insights into remote sites not easily captured by ecosystem-level measurements, which have allowed model estimates of the permafrost carbon-climate feedback^2,7,8^. However, most incubation studies neglect the role of biotic interactions, notably microbial community assembly and its effects on soil functioning, which appear increasingly important in determining post-thaw permafrost functioning in nature^9–12^. Upon permafrost thaw, microbial community composition often changes substantially^12–14^. This can occur through endogenous changes in the microbial community present in the permafrost under the new environmental conditions^15,16^, but also through invasion of surface microorganisms in the newly-thawed permafrost^13,17^. Due to dispersal limitations in permafrost when still frozen^18^, local stochastic extinctions are not counteracted by the dispersal of new microbial species from the active layer. Over tens of millennia, this is thought to decrease diversity and functional redundancy of the microbial community, eventually resulting in communities deprived of certain functions, and therefore hampering or losing specific biogeochemical processes^10^. Thus, the nature and rate of biogeochemical processes resulting in net production of the GHGs carbon dioxide (CO_2_), methane (CH_4_) and nitrous oxide (N_2_O) are not only constrained by soil abiotic and climatic factors, but also rely on microbial community composition^19,20^, in ways that remain poorly understood in permafrost soils^10,21^.

Permafrost occurs across a large part of the Arctic and boreal zone^22^ and includes different soil types and different durations of frozen conditions. Yedoma deposits occur in regions that were not glaciated during the late Pleistocene (c. 84–11.7 ka BP^23^) and are among the oldest permafrost deposits. They store 297 Pg-C and 37 Pg-N (29% of all C and 38% of all N in permafrost^22,24^) and their high ice content puts them at risk of abrupt thaw. Functional limitation of the microbial community was demonstrated in Yedoma permafrost by adding a functionally rich microbial community from a grassland, which introduced new microbial functions that increased CO_2_ emissions and introduced nitrification^10^. The production of CH_4_^9^ and N_2_O^11^ also seems affected by such limitations in Yedoma permafrost, and slow growth of specialized microorganisms in very old Yedoma permafrost could explain these findings^25^. In contrast to the restricted geographical extent of Yedoma, most present-day permafrost, such as those found in palsa peat bogs and sedge-derived peat, or in cryoturbated soils where frost-induced soil movements actively bury organic matter, were formed after the Last Glacial Maximum and have thus experienced perennial frost for a much shorter time. Cryoturbated soils are widespread and store up to 46% of permafrost C^1,26^, and peat permafrost soils store 18% and 7% of permafrost C and N despite their limited and decreasing extent^26–28^. How much of these C and N stocks will be converted to greenhouse gases after thaw is of great interest considering the consequences for climate feedbacks (e.g. refs. ^21,28–30^), but it remains unclear whether functional limitations have also developed in more recent permafrost, or if functional limitations are common for production of different GHG across permafrost types.

Upon natural thaw of permafrost soils, the introduction of microbial functions would need to originate from the microbial communities in the overlying active layer, through, e.g., seepage of groundwater during gradual deepening of the active layer, or mixing of soils due to cryoturbation or thermokarst. This local active layer community can be functionally diverse, but it may also lack certain functions^31^, or may not successfully adapt to different soil conditions or outcompete permafrost microorganisms and establish in newly thawed permafrost. Ice-rich deposits such as Yedoma are found below various types of overlying vegetation, and their high ice content renders them susceptible to abrupt thaw leading to soil layers mixing, which could expose them to a variety of active layer microbial communities. Although post-thaw microbial community dynamics are now actively studied (e.g., refs. ^17,21,32^), it remains unclear whether different active layer microbial communities can introduce potentially missing or lost functions to different thawing permafrost types and thereby alleviate functional limitations.

**Fig. 1:**
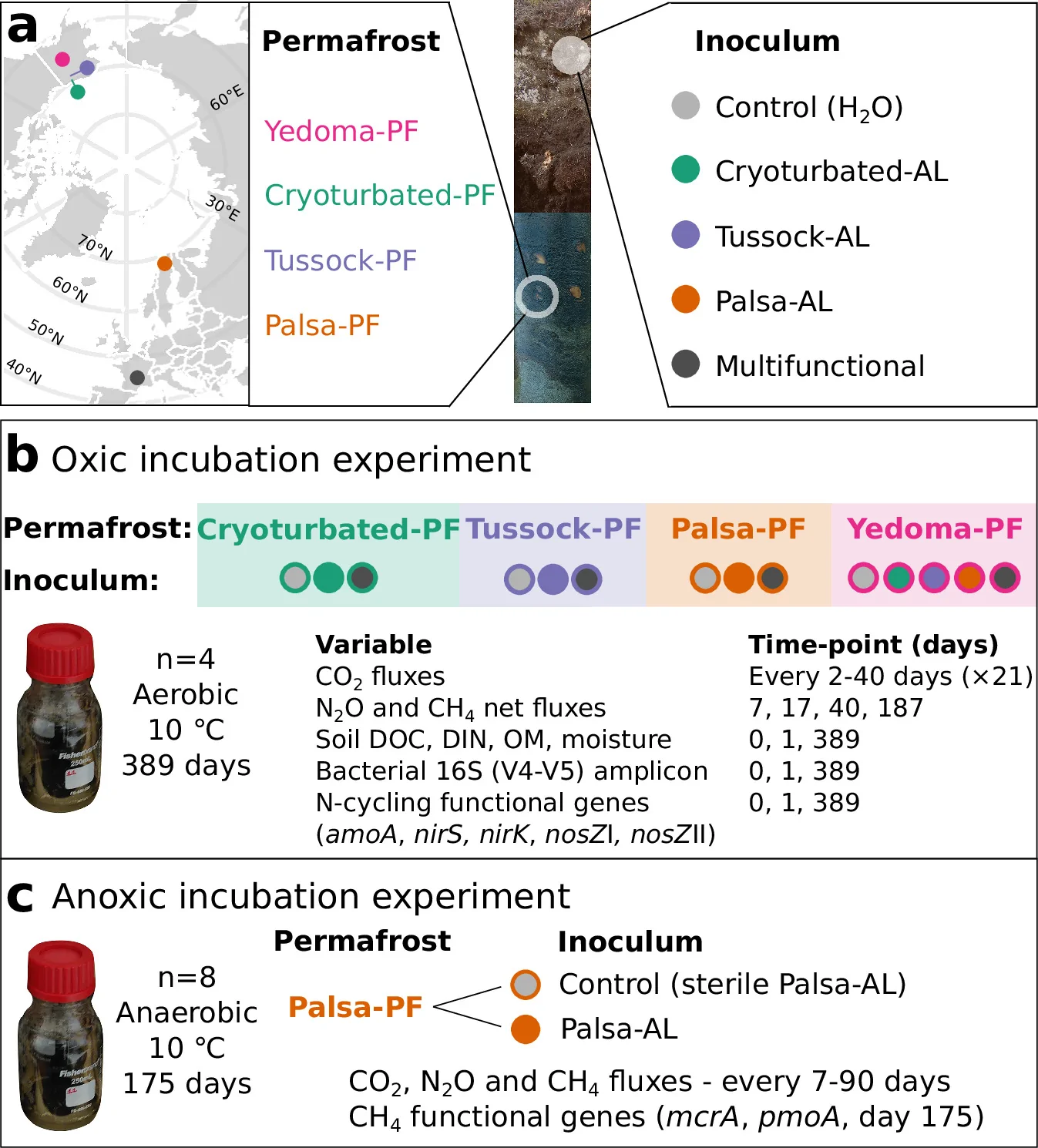
Experimental design. **a** Sampling sites for permafrost and active layer soils. Picture credits Sylvain Monteux, Konstantin Gavazov. Made with Natural Earth. Free vector and raster map data @ naturalearthdata.com. Natural Earth Data accessed through rnaturalearth^101^. **b** In the oxic incubation experiment, four permafrost soils were inoculated with a multifunctional inoculum from a temperate soil and three with their respective active layer to explore, respectively, whether they harbored functional limitations and whether their active layer microbial communities would alleviate those. All three active layer inoculums were used in Yedoma permafrost, to compare their ability to alleviate functional limitations in Pleistocene-old, ice-rich permafrost. **c** In the anoxic incubation we explored functional limitations of CO_2_, N_2_O, and CH_4_ production in the Palsa permafrost.

We compared the occurrence of functional limitations of CO_2_, N_2_O, and CH_4_ production across four common permafrost types of different origin and ages (palsa peat, tussock peat, cryoturbated soil and Yedoma sediment), and tested the ability of active layer microbial communities to alleviate such limitations, through two incubation experiments (Fig. 1). In the first experiment, we incubated permafrost soils from a palsa peat bog, a sedge peat from moist acidic tussock tundra, and a cryoturbated soil, under oxic conditions for 389 days and compared dynamics of CO_2_ and N_2_O production and nitrogen (N) pools when incubated with a soil microbial inoculum from their own active layer, a functionally rich microbial community from a temperate grassland as in ref. ^10^ (Multifunctional), and no microbial inoculum (Control). Yedoma sediment was incubated alongside the other soils with Control, Multifunctional, and all three active layer inoculum types to compare their potential within this type of deposit. In the second experiment, we incubated palsa permafrost under anoxic conditions for 175 days to explore whether inoculation with its own active layer microbial community would stimulate anaerobic GHG production. We hypothesize that (1) missing functions in microbial communities limit biogeochemical processes in all permafrost types—although older permafrost deposits may be more strongly affected—, (2) active layer microbial communities can restore missing or stimulate hampered processes by introducing missing functions. We further anticipate that microbial community coalescence events (that is, the interchange of entire microbial communities upon “invasion” or “establishment”, sensu^33,34^) should be observed wherever functional limitations are detected, while failing to induce coalescence would suggest that microorganisms with missing functions could not establish.

## Results

### Greenhouse gases: oxic CO_2_ production

**Fig. 2:**
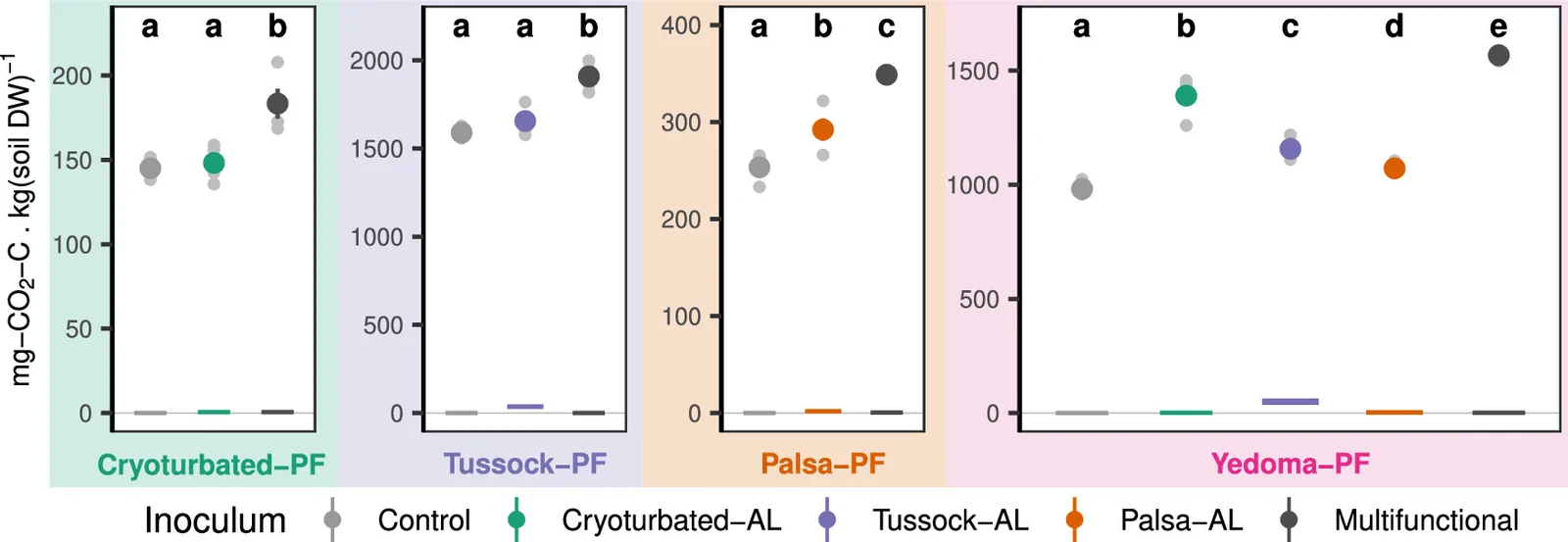
Cumulative CO_2_ production of four permafrost soils subjected to microbial community manipulation after 389 days of oxic incubation. Large symbols and error-bars indicate mean +/− SE (*n* = 4), small gray dots are individual measurements. Horizontal bars denote the amount of carbon introduced by the inoculum solutions. Letters denote significant (estimated marginal means Holm-adjusted *P* < 0.05) differences between inoculum treatments within a given soil type. Cryoturbated-PF: ANOVA *F*_2,9_ = 11.65, *P* = 0.003, Tussock-PF *F*_2,9_ = 23.59, *P* = 2.6.10^−4^, Palsa-PF *F*_2,9_ = 36.84, *P* = 4.6.10^−5^, Yedoma-PF *F*_4,15_ = 92.95, *P* = 2.1.10^−10^. Source Data are provided as a Source Data file.

We found clear limitations of CO_2_ production in all soils. The Multifunctional inoculum (addition of a functionally diverse community) enhanced the production of CO_2_ in all soils, with increases in cumulative CO_2_ production ranging from 20.1 to 59.6 % (Fig. 2 and Supplementary Fig. S1). CO_2_ production in the Palsa-PF permafrost increased significantly in response to its own active layer inoculation (+15.4 %, 95% CI 8.3–22.5 %, *t* = 3.47, d*f* = 9, *P* = 0.007), while neither the Tussock-PF nor Cryoturbated-PF responded when inoculated with their respective active layer community. For the Yedoma-PF deposit, all three active layer inoculums significantly increased CO_2_ production, but to a lesser extent than the Multifunctional inoculum. Further, the order was reversed, with Cryoturbated-AL inoculum causing the greatest increase (41.5 %, CI 36.2–46.6 %, *t* = 11.58, *P* < 0.001; d*f* = 15), Tussock-AL intermediate (17.8 %, CI 12.4–23.2 %, *t* = 4.98, *P* = 0.001), and Palsa-AL the smallest (9.1 %, CI 3.7–14.1 %, *t* = 2.55, *P* = 0.044, Fig. 2).

### Greenhouse gases: oxic N_2_O production

**Fig. 3:**
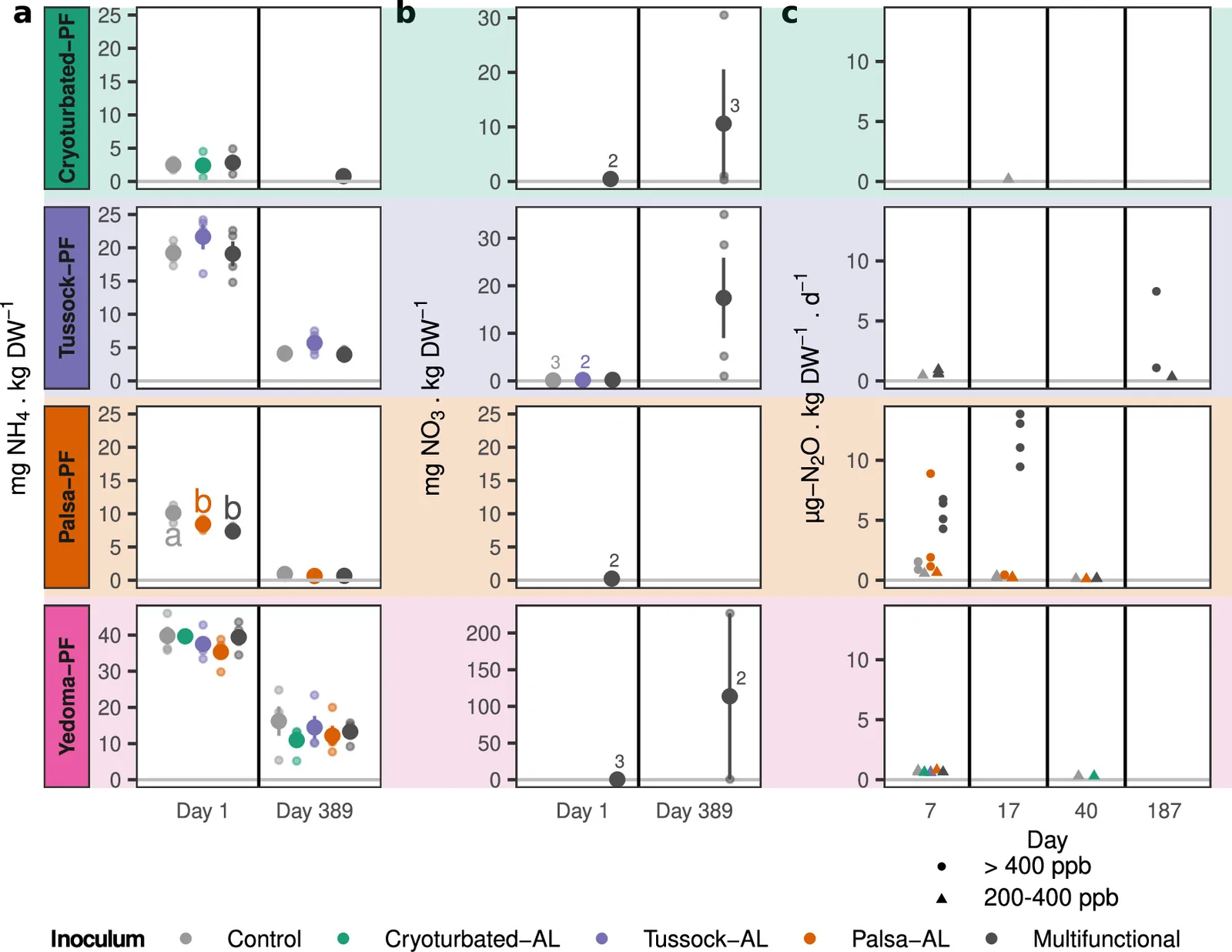
Dissolved mineral nitrogen pools and net N_2_O emissions in four permafrost soils subjected to microbial community manipulation over 389 days of oxic incubation. Water-extractable dissolved inorganic nitrogen pools ammonium (**a**) and nitrate+nitrite (**b**), and net N_2_O flux rates (**c**). Large symbols in (**a**) and (**b**) indicate mean +/− SE, (*n* = 4 unless indicated otherwise by numbers when below detection limit), different letters denote statistically significant differences (Supplementary Table S3, estimated marginal means Holm-adjusted *P* < 0.05). Small symbols represent individual measurements; triangles denote trace amounts shown and discussed but excluded from statistical analyses (based on concentration in samples before back-calculating to fluxes), measurement values below 200 ppb (0.5 × detection limit) are not shown. Ammonium: Cryoturbated PF day 1 ANOVA *F*_2,9_ = 0.11, *P* = 0.898, day 389 not applicable, Tussock-PF day 1 *F*_2,9_ = 0.80, *P* = 0.478, Tusssock-PF day 389 *F*_2,9_ = 3.05, *P* = 0.097, Palsa-PF day 1 *F*_2,9_ = 11.21, *P* = 0.004, Palsa-PF day 389 *F*_2,9_ = 1.33, *P* = 0.311, Yedoma-PF day 1 *F*_4,15_ = 1.05, *P* = 0.41, Yedoma-PF day 389 *F*_4,15_ = 0.65, *P* = 0.636. N_2_O Palsa-PF day 7 Kruskal-Wallis *χ*^2^ = 5.11 *P* = 0.077. Source Data are provided as a Source Data file.

Inorganic N dynamics and N_2_O production provide further indication of functional limitations, as they strongly responded to inoculation, mainly by the Multifunctional inoculum. At the end of the incubation, ammonium had decreased in all soils regardless of inoculation, by 60%–100% (Fig. 3a). Very high nitrate and nitrite contents, in some cases far exceeding initial ammonium concentrations, were observed in soils inoculated with the Multifunctional inoculum, except for the Palsa-PF soil where nitrate was no longer detected (Fig. 3b). Overall, N_2_O remained below detection limit in most soils regardless of inoculation, with N_2_O detected mostly in soils inoculated with the Multifunctional inoculum (Tussock-PF, Palsa-PF) and Palsa-AL active layer (Palsa-PF). In the Palsa-PF soil, net N_2_O emissions were initially (day 7) higher in the Multifunctional inoculum than in the control or the active layer inoculum (Fig. 3), with a + 425% increase in the Multifunctional inoculum compared to the control (Fig. 3; Control: mean ± SE 1.32 ± 0.37 µg N_2_O-N day^−1^ kg dry soil^−1^ and 95%CI 0.60–2.05, *n* = 3; Multifunctional mean ± SE 5.65 ± 1.15 CI: 3.39–7.90, *n* = 4). However, this increase was not statistically significant due to the measurements below the detection limit and the non-parametric test (Kruskal-Wallis *Χ*^2^ = 4.3, d*f* = 2, *P* = 0.112). After 17 days, N_2_O was detected in the Palsa-AL active layer treatment (*n* = 2, mean ± SE: 0.45 ± 0.02 µg-N_2_O-N day^−1^ kg dry soil^−1^) and several orders of magnitude higher in the Multifunctional inoculum (*n* = 4, mean ± SE: 11.86 ± 0.99 µg-N_2_O-N day^−1^ kg dry soil^−1^), but this increase was not statistically tested due to the low sample size. Nitrous oxide was further found in trace amounts (200–400 ppb) in the control and Palsa-AL active layer treatment (*n* = 4 and 2, respectively). N_2_O was only observed in trace amounts at day 40 and was below the detection limit at day 187 (400 ppb equivalent c 0.4–1.4 µg N_2_O-N kg soil DW^−1^d^−1^ over days 180–187). In the Tussock-PF soil, N_2_O was detected in trace amounts at day 7 in the control and Multifunctional inoculum (in 1 and 4 replicates, respectively), then at day 187 in large amounts in the Multifunctional inoculum treatment only (*n* = 2). In the Cryoturbated-PF and Yedoma-PF soils, N_2_O remained below the detection limit in all treatments at all time points, and was only occasionally detected in trace amounts.

### Greenhouse gases: anoxic CH_4_ production

**Fig. 4:**
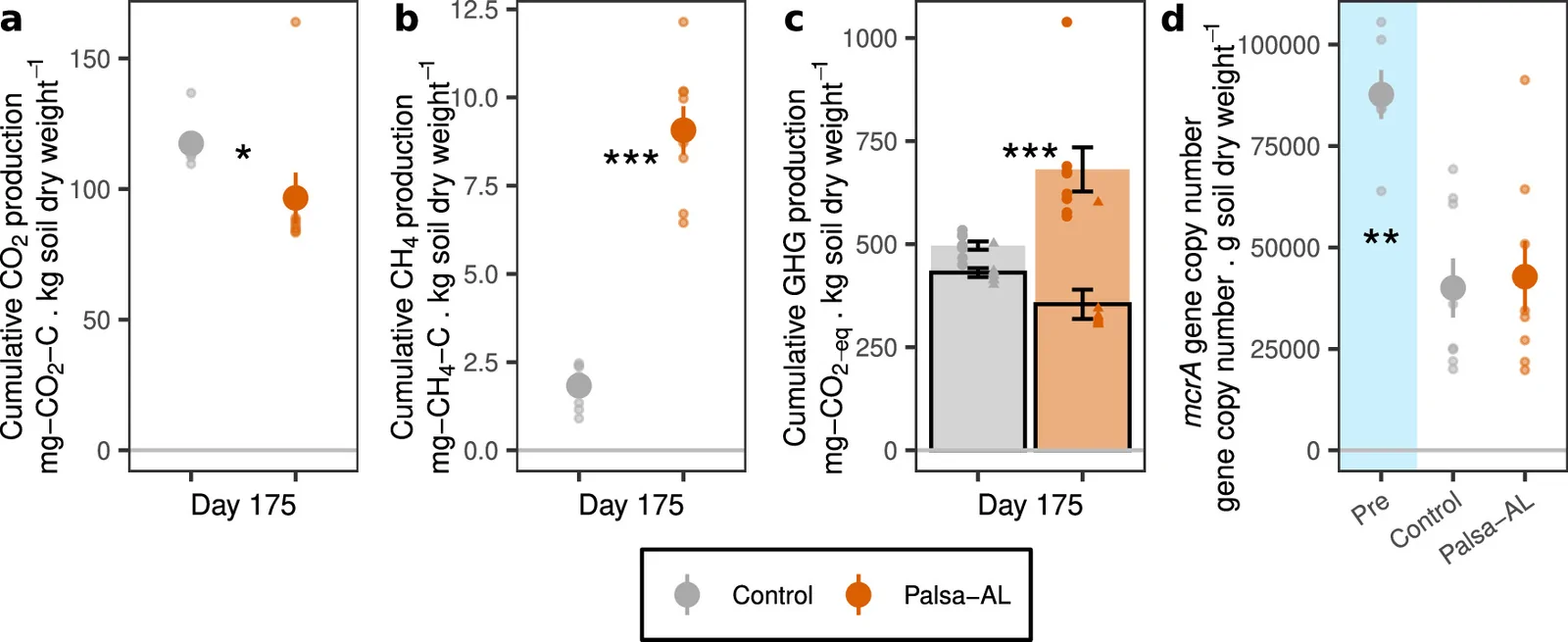
Cumulative greenhouse gases emissions in Palsa permafrost subjected to microbial community manipulation over 175 days of anoxic incubation. Cumulative CO_2_ (**a**), CH_4_ (**b**), CO_2-eq_ (**c**) production and methanogenesis functional gene (**d**). Symbols and error-bars indicate mean +/− SE (*n* = 8), small symbols are individual measurements. Colored bars and dots in (**c**) indicate CO_2-eq_, black outline bars and triangles denote the CO_2_ fraction of CO_2-eq_ (data from a converted from mg-CO_2_-C to mg-CO_2_). Blue background in (**d**) denotes pre-incubation permafrost. Symbols denote statistically significant differences (***: *P* < 0.001; **: *P* < 0.01; *: *P* < 0.05). **a**, **c**: two-sided Wilcoxon rank sum exact test, **a**: *W* = 56, *P* = 0.010; **c**: *W* = 0, *P* = 2.10^−4^; **b**: two-sided t-test with unequal variances, *t* = −10.51, d*f* = 11.11, *P* = 4.10^−7 ^d: one-way ANOVA, *F*_2,19_ = 10.7, *P* = 8.10^−4^ and estimated marginal means. Source Data are provided as a Source Data file.

Methane production in the Palsa-PF soil under anoxic conditions strongly responded to inoculation with its own active layer compared to the control (RM-ANOVA *F*_8,84_ = 61.22, Greenhouse-Geisser ε corrected-*P* < 0.001), resulting in a quintupling of cumulative CH_4_ production over six months (Fig. 4b; +395 %, CI 339–452 %, *t* = 10.51, d*f* = 21, *P* < 0.001), while CO_2_ production significantly decreased (*W* = 56, *P* = 0.01, d*f* = 15, Fig. 4a), and N_2_O was not detected. Cumulative estimated warming potential (using GWP_100_ for non-fossil CH_4_ as conversion factor) increased by 35% with inoculation (CI 15–59%, *t* = 8.27, *P* = 0.003, Fig. 4c). Gammaproteobacterial *pmoA* genes could not be quantified at the end of the anoxic incubation, but were detected in pre-incubation Palsa-PF permafrost. The amount of *mcrA* gene copies (Fig. 4d) was unaffected by inoculation but was higher in pre-incubation permafrost than after the incubation.

### Bacterial communities and inoculation effects

Bacterial community coalescence and increased bacterial richness were observed in some inoculation treatments. These changes coincided with biogeochemical changes described in previous sections.

Before the incubation, the bacterial communities in the different active layers and positive control soils differed significantly from each other (manyglm pairwise comparisons *P* = 0.018, *n* = 4). The bacterial communities in permafrost soils prior to incubation also appeared to differ from each other and from active layer soils, although this could not be formally tested as too few replicates passed quality filtering (Supplementary Fig. S2).

**Fig. 5:**
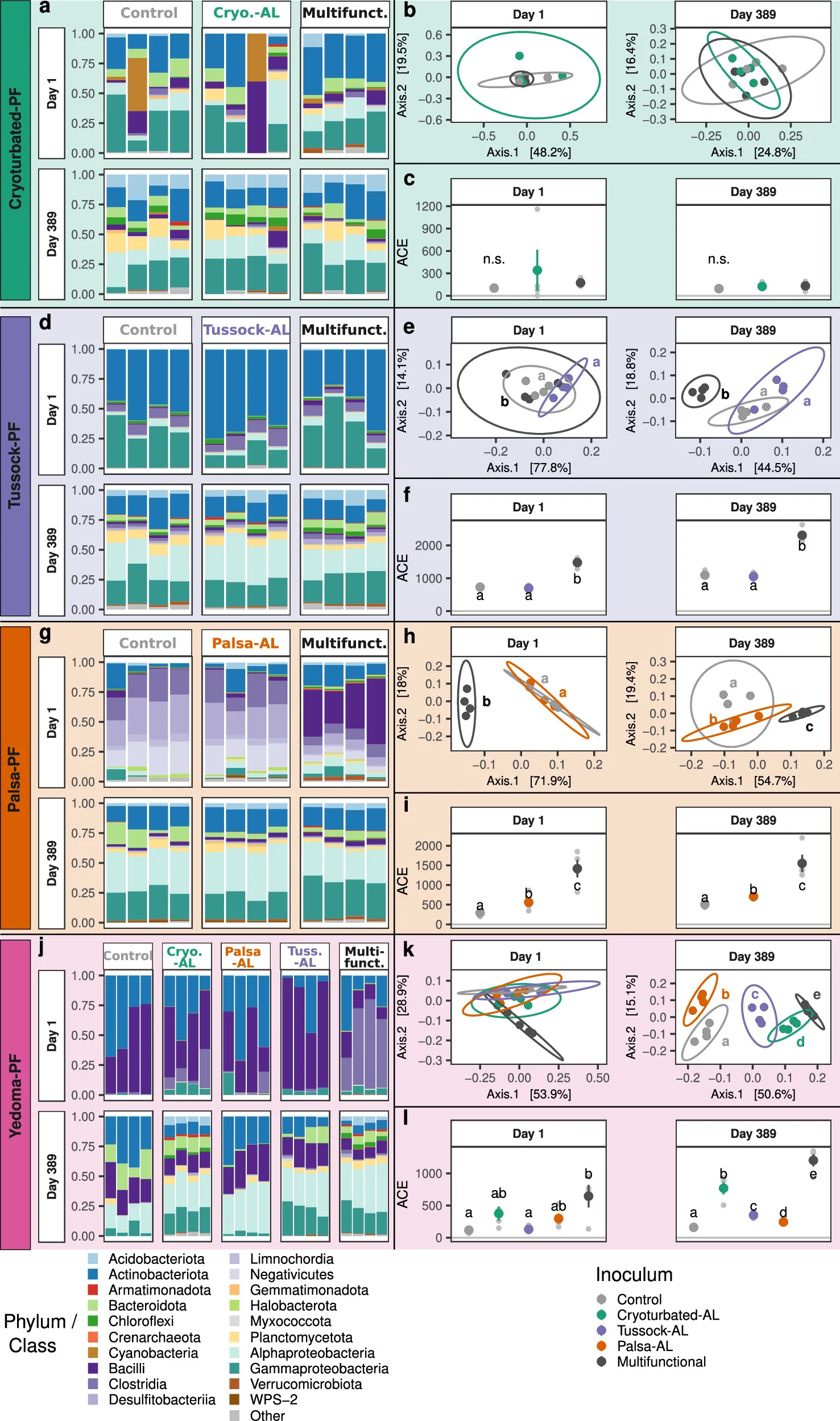
Bacterial community composition (16S V4V5 amplicons) in four permafrost soils subjected to microbial community manipulation over 389 days of oxic incubation. **a**, **d**, **g**, **j**: Relative abundance of the 20 most abundant bacterial phyla (class for Firmicutes and Proteobacteria), each stack of bars represents one sample. **b**, **e**, **h**, **k**: Principal coordinate analyses (PCoA) of weighted UniFrac distances. Points represent individual samples, ellipses indicate 95% confidence intervals for each treatment (*n* = 4). Different lower-case letters indicate significant differences within a soil-date combination (Supplementary Table S1, manyglm ANOVA pairwise comparison Holm-adjusted *P* < 0.05). **c**, **f**, **i**, **l**: Species richness estimate (abundance-based coverage estimator ACE). Large symbols and error-bars represent means +/− SE (*n* = 4), small symbols are individual measurements, different letters indicate significant differences within a soil-date combination (ANOVA *P* < 0.05, estimated marginal means Holm-adjusted *P* < 0.05). Cryoturbated-PF day 1 Kruskal-Wallis *χ*^2^ = 3.73, d*f* = 2, *P* = 0.155, Cryoturbated-PF day 389 ANOVA *F*_2,8_ = 0.40, *P* = 0.683, Tussock-PF day 1 *F*_2,9_ = 70.98, *P* = 3.1.10^−6^, Tussock-PF day 389 *F*_2,9_ = 63.86, *P* = 4.1.10^−6^, Palsa-PF day 1 *F*_2,9_ = 21.79, *P* = 3.6.10^−4^, Palsa-PF day 389 *F*_2,9_ = 44.46, *P* = 2.2.10^−5^, Yedoma-PF day 1 *F*_4,15_ = 5.03,*P* = 0.009, Yedoma-PF day 389 *F*_4,15_ = 89.62, *P* = 2.8.10^−10^. Source Data are provided as a Source Data file.

**Fig. 6:**
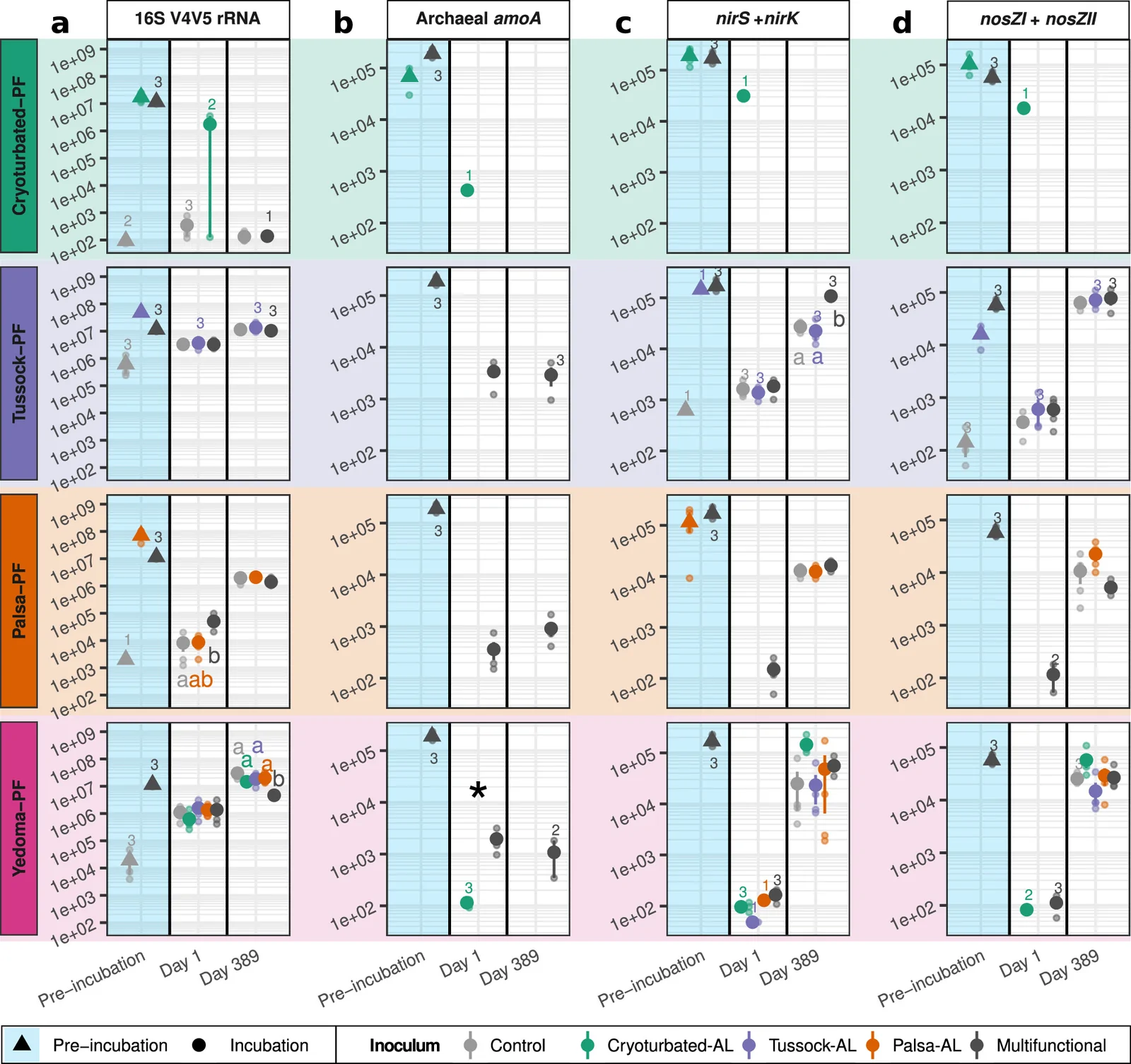
Ribosomal and nitrogen cycling functional genes in four permafrost soils subjected to microbial community manipulation over 389 days of oxic incubation. Abundance of ribosomal gene (**a**) and genes controlling nitrification (**b**), production (**c**) and consumption (**d**) of N_2_O. Gene copy number per g soil dry weight. Symbols and error-bars indicate mean +/− SE (*n* = 4 unless indicated otherwise by numbers when below detection limit), letters indicate statistically significant differences within a soil:date combination (Supplementary Table S2, estimated marginal means Holm-adjusted *P* < 0.05), asterisk denotes significant differences within a soil:date combination (two-sided t-test *P* < 0.05). Pre-incubation samples are the respective permafrost soils prior to incubation (Control), active layer (Turbel-AL, Palsa-AL, Tussock-AL), and Multifunctional soils used for inoculum suspensions. 16S rRNA Tussock-PF day 1 ANOVA *F*_2,8_ = 0.08, *P* = 0.926, Tussock-PF day 389 *F*_2,7_ = 0.44, *P* = 0.661, Palsa-PF day 1 *F*_2,9_ = 5.85, *P* = 0.024, Palsa-PF day 389 *F*_2,9_ = 2.42, *P* = 0.144, Yedoma-PF day 1 *F*_4,14_ = 1.08, *P* = 0.400, Yedoma-PF day 389 *F*_4,15_ = 15.68, *P* = 3.1.10^−5^. Archaeal *amoA* Tussock-PF day 1 *t* = −3.87, d*f* = 3, *P* = 0.031. Sum of *nirS*+*nirK* Tussock-PF day 1 *F*_2,7_ = 0.47, *P* = 0.642, Tussock-PF day 389 *F*_2,7_ = 20.66, *P* = 0.001, Palsa-PF day 389 *F*_2,9_ = 1.63, *P* = 0.249, Yedoma-PF day 389 *F*_4,15_ = 3.38, *P* = 0.037. Sum of *nosZI*+*nosZII* Tussock-PF day 1 *F*_2,8_ = 0.61, *P* = 0.568, Tussock-PF day 389 *F*_2,7_ = 0.10, *P* = 0.911, Palsa-PF day 389 *F*_2,9_ = 3.76, *P* = 0.065, Yedoma-PF day 389 *F*_4,14_ = 2.94, *P* = 0.059. Source Data are provided as a Source Data file.

One day after inoculation the composition of the bacterial communities was only affected by inoculation with the Multifunctional inoculum in the Tussock-PF and Palsa-PF permafrost soils (Fig. 5). Tussock-PF and Yedoma-PF soils also showed an increase in bacterial richness in the Multifunctional inoculum, and the Palsa-PF soil with both the Palsa-AL active layer and Multifunctional inoculum (Fig. 5). Bacterial abundance, estimated by the number of 16S rRNA gene copies, was initially unaffected by inoculation, except for the Palsa-PF soil where the samples with the Multifunctional inoculum were one order of magnitude higher than the control and the active layer inoculum (respectively *t* = 3.19 and 2.66, *P* = 0.033 and 0.052, d*f* = 9, Fig. 6).

After 389 days of incubation, bacterial community composition in all inoculation treatments differed significantly from each other and the control in the Palsa-PF and Yedoma-PF permafrost (manyglm post-hoc pairwise comparisons *P* < 0.05, Supplementary Table S1 and Fig. 5). In the Tussock-PF soil, the Multifunctional inoculum differed from the control, but the Tussock-AL active layer inoculum treatment did not, while in the Cryoturbated-PF soil no significant differences were observed between treatments. Bacterial richness estimates corresponded to changes in community composition, with significant increases in estimated richness only in cases where communities significantly differed from the control (Supplementary Table S2). Bacterial biomass was 1–3 orders of magnitude higher at the end than at the beginning of the incubation, regardless of inoculation treatments, except for the Yedoma-PF sediment where the Multifunctional inoculum was one order of magnitude below the control and Palsa-AL inoculum (*t* > 3.11, *P* < 0.02, d*f* = 15, Fig. 6).

### Linking bacterial communities and CO_2_ production

We explored associations between CO_2_ production on one hand, and soil chemistry and bacterial community composition on the other, by using RandomForest to identify the most important variables. Of the 107 edaphic and bacterial variables explored, 93 were considered informative for interpretation by VSURF, nine showed a significant (*P* < 0.05) increase in both mean squared error and node purity in rfPermute, and these nine were retained by both approaches. These nine variables were all bacterial classes, and we further explored which exhibited consistent responses in all soils (except Cryoturbated-PF), in the shape of a significant association between relative abundance and net CO_2_ production (Supplementary Fig. S3). Five bacterial classes significantly correlated with CO_2_ production, all positively: *Acidobacteriota Subgroup 5*, *Dehalococcoidia*, *Fimbriimonadia*, *Holophagae*, and *Nitrososphaeria*. Those are mainly poorly characterized groups with few cultured relatives, limiting our interpretation; however, three ASVs in *Holophagae* could be identified as *Geothrix* sp. and *Geothrix fermentans*, while all *Nitrososphaeria* ASVs were *Nitrososphaeraceae*, including two *Nitrosocosmicus* sp. The prevalence of ASVs resolved at the genus- and species-level corresponded to those observed at the class level, that is, were mostly observed with Multifunctional and Cryoturbated-AL inocula. However, not all ASVs followed identical correlations as their classes, implying that other ASVs within those classes, not resolved at the genus or species level, were responsible for the observed correlations.

### Functional genes

Archaeal *amoA* genes coding ammonia oxidation in the nitrification process were prevalent in the Cryoturbated-AL active layer soil, but were otherwise in low abundance (100–400 copies per g dry soil) or below detection at day 1, and not detected at day 389 unless inoculated with the positive control (Fig. 6). Bacterial *amoA* genes were not detected. By contrast, *nir* and *nosZ* genes were detected in fairly equal amounts in all soils by the end of the incubation, regardless of inoculation, even though they were either low in abundance or undetected on day 1 (Fig. 6). As a proxy for N_2_O source vs sink potential, the *nir*/*nos* ratio was largely unaffected by inoculation (Supplementary Fig. S4), except for a significant increase in Tussock-PF soil, with the Multifunctional inoculum being the only treatment with a *nir*/*nos* ratio higher than 1, suggesting a stronger source than sink potential. In the Palsa-PF soil, the Multifunctional inoculum had significantly higher *nir*/*nos* ratios than the Palsa-AL active layer, suggesting a sink in the active layer and a source in the Multifunctional inoculum, but neither differed from the control. Only minute amounts of 16S rRNA and N-cycling genes were detected in the Cryoturbated-PF permafrost soil overall and therefore not interpreted further. This suggests low biomass in this mineral soil, and larger soil amounts may have allowed for better DNA-based results. We recommend future studies to increase soil amounts for mineral soils with low biomass.

### Soil chemistry

Changes in soil chemistry upon inoculation could obfuscate impacts of microbial community dynamics; thus, we assessed whether soil chemistry variables were affected one day after inoculation. Soil moisture was unaffected by inoculation treatments, while other factors were, although mainly in the Palsa-PF soil. Here, soil pH was affected by inoculation, with increased pH for the Multifunctional and Palsa-AL inoculum compared to the control, by 0.68 and 0.90, respectively (±0.23, *t* = 2.90 and 3.85, *P* = 0.042 and 0.010, respectively, d*f* = 9, Supplementary Table S3). Likewise, total dissolved N, ammonium, and dissolved inorganic N were slightly lower after inoculum addition in the Palsa-PF soil. There were small decreases in water-extractable DOC in two inoculated soils relative to the control (Tussock-PF and Palsa-PF *P* = 0.010 and 0.003, respectively), and a small decrease in DON in Tussock-PF but only when inoculated with its own active layer (Tussock-AL, Supplementary Fig. S5). Nitrate and nitrite were undetected at day 1, except in some Tussock-PF soils and in samples inoculated with the Multifunctional inoculum. We further explored putative artefacts due to chemistry alteration upon inoculation in Supplementary Text.

At the end of the oxic incubation, day 389, dissolved inorganic N was significantly higher in the positive control inoculum treatments, in both Cryoturbated-PF and Tussock-PF (Supplementary Table S3 and Supplementary Fig. S5). Water-extractable total dissolved N had increased up to 9-fold compared to the start of the incubation, with water-extractable organic N increasing 2 to 9-fold (Supplementary Fig. S6), irrespective of inoculation. Dissolved organic C content decreased significantly for the positive control in Yedoma-PF sediment. Other soil variables (SOM content, pH, moisture, total and organic N) were unaffected by inoculation treatments, with the exception of inorganic N pools.

## Discussion

Functional limitations were observed in all permafrost soils, as evidenced by large increases in CO_2_ production (20%–60%, Fig. 2), changes in inorganic N pools (Fig. 3), and the introduction of missing N-cycling genes (Fig. 6) when inoculated with the Multifunctional inoculum. In addition, we found indications of functional limitation of N_2_O and of CH_4_ production in some of the permafrost soils. Our hypothesis that changes in biogeochemistry would correspond to changes in bacterial community composition was largely supported, as changes in dynamics of GHG production or inorganic N pools were only detected in cases where bacterial community composition was significantly modified by inoculation. The exception was the Cryoturbated-PF soil, where DNA-based results indicate low biomass. Strong and long-lasting changes in community composition, systematically linked to an increase in bacterial richness, indicate that coalescence events^33^ occurred between permafrost and inoculum communities (Figs. 2 and 5). The framework of microbial functional limitations of biogeochemical processes in permafrost, hitherto only shown in Pleistocene-old Yedoma deposits^9–11^ is therefore relevant across all common types of permafrost, even in more recent Holocene deposits. “Not all permafrost microbiomes are created equal”, as Barbato and colleagues^16^ described the locally important variation in permafrost functional potential. We also see such variation across soils, and further research should clarify whether this stems from functions missing upon permafrost aggradation^35,36^, lost over time due to selection and drift^37^, or a combination of these mechanisms. Nevertheless, functions appear missing or lost from permafrost microbial communities not only in permafrost frozen for dozens of millennia^25^ but also in Holocene deposits a few centuries to a few thousand years old (e.g. Palsa-PF < 9600 cal.BP, Tussock-PF ~ 8000 cal.BP^38,39^).

Microbial communities from local active layers were not consistently able to alleviate the functional limitations to the same extent as our multifunctional inoculum. This could be because the microbial communities in the active layers were missing the same functions as those in the underlying permafrost layers, which agrees with the selected N-cycling functional genes not always being observed in the active layer soils we used for inoculation (Fig. 6). This corroborates observations of truncated denitrifier communities in topsoils, notably in the tundra^31,40^, but it is unclear how common this is over space and soil depth throughout the Arctic. *nir* and *nosZ* genes in denitrifiers were likely present from the beginning in amounts below the detection limit of our qPCR assays, in both the active layer inoculums and permafrost soils. However, *nir* and *nosZ* gene copy numbers within a given soil were similar in abundance by the end of the incubation regardless of inoculation, indicating equal capacity to produce and consume N_2_O. Thus, the active layer samples we used for inoculation were likely missing some functions, but not all. Another explanation is that microbial communities in active layer soils carried the functions missing from the permafrost, but failed to establish in the permafrost and introduce these missing functions. Indeed, successful coalescence events appear as a necessary condition to the restoration of missing functions. All permafrost soils were susceptible to coalescence at least with the Multifunctional inoculum (with the exception of the Cryoturbated-PF soil), and all active layer inocula induced coalescence in the Yedoma-PF sediment (Fig. 5), ruling out bacterial communities in the inocula being unable to establish in permafrost soil. Our experiments were not designed to answer which drivers favor or impede microbial community coalescence in newly-thawed permafrost, but our ancillary measurements offer insights. Differences in pH, organic matter content, moisture content, or DOC between permafrost and active layer did not provide consistent explanations for the (lack of) success of coalescence (Supplementary Fig. S5). Therefore, this suggests that the success of coalescence could depend on host- or guest- soil community or soil variables beyond our range of measurements. While the factors affecting modalities and consequences of microbial community assembly upon thaw are increasingly studied^12,17,21,41^, combinations of field and laboratory experiments distributed across the permafrost region will be critical to assess the (lack of) functionality of active layer microbial communities, the controls over coalescence dynamics and their biogeochemical implications.

The Palsa-AL inoculum, which was the only active layer inoculum to induce a significant increase in CO_2_ production in its own permafrost, induced the lowest increase in Yedoma-PF permafrost. Conversely, the Cryoturbated-AL inoculum, which did not increase CO_2_ production in Cryoturbated-PF permafrost, induced the highest increase in Yedoma-PF. This suggests that the impact of coalescence events is difficult to predict, but may depend on the complementarity between functions present in the permafrost and active layer, as well as on microbial factors determining the success of coalescence. We speculate that certain functions may be entirely missing in both active and permafrost layers, but that those functions differ across soil types or space. Under this assumption, microbial dispersal through percolation upon active layer deepening may not efficiently restore the limited functional potential of permafrost microbial communities. More disruptive permafrost thaw events involving soil mixing, such as thaw slumps or thermokarst features, may have greater impacts on microbial communities, as additional vectors of dispersal, such as wind^42,43^ or soil fauna^44^ could then accelerate microorganism migration into newly-thawed permafrost. Increasing anthropic pressure in the Arctic, notably through tourism and mining, may also increase microbial dispersal^45^, which could harmonize the functionality of active layer microbial communities. Moreover, microbial communities vary over depth within the active layer^14,46^, although not systematically^13,41^, therefore, our inocula from well-drained topsoils may differ in community, functions, and impacts from those located deeper. Understanding the variability of active layer microbial communities and of their potential to alleviate permafrost functional limitations is essential to assess the feasibility and impact of measures mitigating microbial dispersal.

Across the oxic incubations, we identified five bacterial classes associated with increased CO_2_ production. Most of those remain poorly characterized, with none or few cultured representatives, but among the *Holophagae* the Tussock-PF contained an ASV identified as *Geothrix fermentans*. This species is known for Fe(III) reduction, which has been suggested as a strong driver of post-thaw metabolism^47^. While strictly anaerobic, it could have developed in anaerobic microsites during the incubation. The prevalence of *Nitrososphaera* correlated with CO_2_ production across soil types, and all archaeal ammonia oxidizers belong to *Nitrososphaera*. Two ASVs in that class were identified as *Nitrosocosmicus* sp., a well-studied genus of ammonia oxidizers^48,49^. Although mainly present in samples inoculated with Multifunctional inoculum, *Nitrososphaera* were also found in the Tussock-AL and Cryo-AL inoculated soils, but in none of the Controls. Nitrifiers were absent from Control soils, and the lower CO_2_ production observed in Control soils thus results in a strong association between the presence of nitrifiers and increased CO_2_ production, but this remains correlative, and more causal relationships deserve to be further explored.

Nitrous oxide production in oxic incubations increased in Palsa-PF soil when inoculated with both the active layer and Multifunctional inoculum, and in the Tussock-PF soil, N_2_O production was only detected in the Multifunctional inoculum. The rates of net N_2_O production (0.44–13.86 µg N_2_O-N kg DW^−1^ d^−1^) are within the range of those in the literature (Supplementary Table S6,^11,50–56^) under similar settings, i.e. oxic conditions without plants. The processes governing N_2_O production are therefore affected by functional limitations in these two soils, and not only in Yedoma soils as previously observed^11^. However, the abundance of genes indicating the capacity for production of N_2_O from NO_2_^−^ reduction (*nirK* and *nirS*) and the reduction of N_2_O into N_2_ (*nosZI* and *nosZII*)^57^ in the denitrification pathway did not differ between treatments, as they indicated a balanced capacity. This could explain the low levels of N_2_O detected, or alternatively there was no N_2_O production due to unfavorable conditions. Complete denitrification to N_2_ may have occurred before we could detect NO_3_^-^ or N_2_O since the gases used to flush the headspace between measurements did not contain N_2_O and we therefore could not detect or quantify net N_2_O uptake. Together with the introduction of archaeal *amoA* genes by the positive control, and the observation of NO_3_^-^ only where archaeal *amoA* genes were introduced, this suggests that the observed functional limitations of net N_2_O production are linked to a limited oxidation process of NH_4_^+^ to NO_3_^-^ (nitrification process). This interpretation is consistent with recent genomic evidence from Siberian permafrost^58^, but gross nitrification rates measurements would be required to confirm it. Nitrous oxide could also be produced during nitrification, but ammonia-oxidizing bacteria are known to produce more N_2_O than the archaeal counterpart^59^, yet bacterial *amoA* genes could not be quantified and only archaeal *amoA* genes were introduced with the positive control. Altogether, this suggests that N_2_O production is mainly limited by the supply of NO_3_^−^ for denitrification and not the absence of organisms with functional capacity to denitrify. All permafrost soils offered hospitable conditions for archaeal nitrifiers, but their low prevalence in active layer soils questions the likelihood of their establishment in situ. The primers used for bacterial *amoA* quantification do not target *amoA* in complete nitrifying bacteria (comammox), which have an oligotrophic lifestyle^60^ and were recently shown to be abundant in Arctic soils^61^. Based on the bacterial sequences, at the end of the incubation, comammox taxa were, however, only observed in two samples that were not inoculated with the positive control, although our 16S rRNA gene primers may not have detected all complete nitrifiers within the genus *Nitrospira*^62^. Contrary to our previous findings^10^, NH_4_^+^content decreased in all soils throughout the incubation, regardless of inoculation, which we therefore cannot entirely attribute to ammonia oxidation. Perhaps, since microbial biomass increased by 1–2 orders of magnitude during the incubation, assimilation of NH_4_^+^into microbial biomass would also explain the decrease. Overall, unclear N dynamics remain at play, but a ubiquitous and large increase in organic N over the incubation (Supplementary Fig. S6) was detected in all soils regardless of inoculation. Further studies should explore N-cycling dynamics in greater detail, for instance by linking gross rates and fluxes of N-transformation with transcriptomics. Plants moreover strongly affect N_2_O emissions^11^ through N uptake, and how they interact with microbial dynamics will require integration in future studies. Our results suggest that in certain permafrost soils, microbial community composition can be a determining factor in the variability of net N_2_O production.

We found functional limitation of methanogenesis under anoxic conditions, evidenced by a quintupling of cumulative CH_4_ production in the presence of active layer inoculum consistently in all eight replicates. This was not balanced by the decrease in CO_2_ production, and in CO_2-eq_ this resulted in a 35% increase in GHG emissions over six months. However, inoculation did not increase the abundance of *mcrA* genes, and the large decrease in *mcrA* copies between pre-incubation and incubated samples suggests that most belong to relic DNA from dormant or dead cells. More detailed analyses based on RNA or SIP could answer this question. In the field, net CH_4_ fluxes correlated with the abundance of Candidatus *Methanoflorens stordalenmirensis* in the active layer of newly-thawed permafrost from a neighboring peatland^63^, and related species may be responsible for the increase we observed. Functional limitations of methanogenesis were originally observed in Pleistocene-old Yedoma permafrost, where CH_4_ production only occurred in isolated permafrost samples in a multi-year incubation, but could be restored by cross-inoculation from other samples where this function was present^9^. Our results expand upon these earlier observations by going beyond Yedoma deposits to more recent Holocene permafrost deposits (SOM dated to c. 9100 yrs BP^38,64^) presumably frozen for a much shorter time. Further, the earlier observations suggested an on-off switch in methanogenesis triggered by the presence/absence of methanogens^9^, but our results instead suggest that even permafrost in which CH_4_ production is observed can produce CH_4_ at much higher rates upon introduction of methanogen communities present in the active layer. Similarly to N_2_O, interactions with plants are an important next step to integrate^65–67^, yet our results highlight that when estimating CH_4_ production from permafrost based on incubations, it is necessary to account for the increased functional potential upon coalescence with active layer microbial communities.

Microbial community coalescence dynamics may affect GHG emissions from thawing permafrost. Therefore, estimates of GHG emissions based on upscaling earlier permafrost incubation results need to account for coalescence dynamics, once we untangle drivers of limitations and quantify their biogeochemical impacts. Field studies, even those manipulating permafrost thaw, e.g., ref. ^13^, cannot distinguish coalescence from endogenous changes without experimental approaches. Such approaches remain confined to laboratories, yet suggest that not only soil mixing^17^ but also liquid inoculums from, e.g., thermokarst ponds can induce coalescence^46^. Our setup resembles percolating water (leachates) and thus likely has a subtler impact than soil mixing scenarios. Field testing across different scenarios of thawing and microbial migration is needed to understand these dynamics in real-life situations to be able to predict their impacts. Laboratory approaches to further our understanding should include interactions with larger soil biota, both fauna and plants, and future upscaling exercises will require regional-scale predictions of active layer microbial communities and their functionality. Meanwhile, we advocate that incubation studies account for these potential dynamics explicitly by comparing presence and absence of an active layer inoculum, or at least by including an active layer inoculum in permafrost incubations.

To conclude, we demonstrate microbial functional limitations affecting three major biogenic greenhouse gases, CO_2_, N_2_O, and CH_4_, across three Holocene permafrost soils as well as in the older Yedoma deposits. Active layer microbial communities could not always entirely alleviate these functional limitations, which we attribute to unsuccessful community coalescence. When they could, they were outperformed by temperate soil microorganisms, probably due to active layer microbial communities missing functions. Upon coalescence with topsoil microorganisms, we observed increases in CO_2_ production of up to 60%, and for N_2_O and CH_4_ up to 400%. The consequences on the Arctic terrestrial permafrost-climate feedback could therefore be globally relevant. We propose that it is crucial to rapidly assess (a) the spatial variability in the functional potential of active layer microbial communities and how this might change in a warmer Arctic, (b) the conditions under which coalescence is possible or impeded, and (c) how other soil biota such as roots, fauna or humans may mediate coalescence. We recommend combining the functional assessment of a collection of circum-Arctic soil samples with mechanistic laboratory and in situ studies untangling the drivers of community coalescence to bridge these knowledge gaps. Such knowledge will be required to elaborate strategies limiting microbial dispersal under increasing anthropic pressure in the Arctic, to predict these expected large increases in biogenic GHG production and account for those in climate change mitigation scenarios.

## Methods

### Soil collection and overall experimental design

The four studied permafrost soils (Fig. 1A) comprise a cryoturbated soil, a tussock tundra sedge peat, a *Sphagnum* palsa peat (both histels), and a Yedoma sediment that together represent the main pools of permafrost soil organic carbon (SOC). Samples from these permafrost soil types and their corresponding active layers were taken in 2012 or 2015 using a SIPRE corer (Jon’s Machine, Fairbanks, Alaska, USA) or a concrete drill bit (⌀ 10 cm) mounted on a gasoline-powered engine. Physical and chemical characteristics of the permafrost and active layer soils are given in Supplementary Table S5. The Cryoturbated soil was sampled from the rim of a non-sorted circle (frost boil) in the Alaskan North Slope (Franklin Bluffs, 69.6685 N, 148.7329 W, 2012, 70–100 cm depth). The sedge peat histel (“Tussock”) was sampled from an *Eriophorum vaginatum*-dominated tussock tundra (moist acidic tundra) of the Alaskan North Slope (Ice Cut, 69.0467 N, 148.8343 W, 2015, 70–100 cm depth). The second histel (“Palsa”) was sampled from a *Sphagnum*-dominated ombrotrophic peat bog underlain by silty permafrost (palsa mire) in sub-Arctic Sweden (Storflaket, 68.3462 N, 18.9714E, 2015, 110–150 cm depth). The Yedoma sediment originated from the Cold Region Research Engineering Laboratory (CRREL) Permafrost Tunnel (Fox, Alaska, USA, 64.9513 N, 147.6206 W, 2012, 15 m depth). The soil was sampled from the upper silt unit originating from an upper Pleistocene silty deposit, previously described in ref. ^10^. Active layer soils used later for inoculum preparation were collected above the water table to reflect the oxic conditions in the incubation (5–15 cm depth), from the same cores as the Cryoturbated, Tussock and Palsa permafrost soils. The functionally diverse soil microbial community used as a positive control (“Multifunctional”) was collected from the surface layer (0–15 cm depth) of a drained Cambisol developed on granitic bedrock at the LTER research site of the French National Research Institute for Agriculture, Food and the Environment (INRAE) in central France (45.7167N, 3.0167E). The site is an experimental grassland, specifically an abandonment-treatment which had not undergone any cutting or fertilization for 15 years prior to soil sampling in winter 2018–2019. The physicochemical and microbiological properties of this soil have been described before^10,68–70^. All samples were stored frozen (−18 °C) until the experiment.

We tested for the existence of functional limitations of CO_2_ and N_2_O production in four permafrost soils, the ability of active layer microbial communities to alleviate these functional limitations, and whether this can be linked to the communities establishing in permafrost (oxic incubation experiment, Fig. 1B). This simulates events such as abrupt thaw, precipitation seepage, or bioturbation, in which newly thawed permafrost becomes exposed to surface soil microbiomes. Each soil was subjected to three separate inoculum treatments: inoculation (1) with its respective overlying active layer (Cryoturbated-AL, Palsa-AL, Tussock-AL); (2) with a functionally diverse microbial community previously used to evidence functional limitations (Multifunctional); or (3) with autoclaved ultrapure water (Control). As Yedoma permafrost may be exposed to a variety of active layer types upon abrupt thaw, we instead subjected it to inoculation with each of the three active layer soils from the other permafrost types (1), as well as to Multifunctional and Control treatments (2) and (3). We further tested for the ability of the Palsa-AL microbial community to alleviate putative functional limitations of CH_4_, CO_2_, and N_2_O production in thawing Palsa-PF permafrost under anoxic conditions (anoxic incubation experiment, Fig. 1C), focusing on this soil as the palsa thaw sequences typically include water-logged, anoxic conditions^71,72^. Here, the permafrost was inoculated with its own active layer and compared to a negative control inoculated with a sterilized sample of its own active layer (Supplementary Text).

In the oxic experiment, each treatment was replicated four times, resulting in 56 individual flasks ([3 permafrost types × 3 inoculation treatments + Yedoma-PF × 5 inoculation treatments] × 4 replicates) per destructive sampling date. Two sets of flasks were prepared to allow a destructive sampling one day after inoculation—to assess putative biases due to inoculation—and at the end of the 389 days of incubation. Flasks were incubated at field moisture content after decanting water from excess ice, under oxic conditions at 10 °C in the dark. This temperature is in range with the mean active-layer temperatures in Arctic regions during summer and is low enough to be within the thermal tolerance range of psychrophilic microorganisms^73^.

Net soil CO_2_ and N_2_O production were quantified on the second set of flasks by measuring cumulative CO_2_ production throughout the incubation period and N_2_O production rates at several time points (7, 17, 40, 187 days). The functional limitation of permafrost microbial communities was defined as a statistically significant (*α* = 0.05) increase in GHG production or a change in N pools in inoculated treatments compared to the negative control. We measured bacterial community composition, abundance, N-cycling functional genes, and pH, dissolved carbon, and N pools of active layer and permafrost soils immediately after their thawing in the lab, one day after inoculation, and at the end of the incubation to monitor changes in community and functional dynamics.

In the anoxic incubation experiment, we incubated the “Palsa-PF” permafrost described above under anoxic conditions and monitored the net change of CO_2_, CH_4_, and N_2_O in the headspace. Each of the Control and inoculated treatments was replicated 8 times with one destructive sampling date, resulting in 16 flasks. We incubated all flasks at 10 °C in the dark under an N_2_ atmosphere for 175 days. We assessed functional limitations of net CO_2_, N_2_O, and CH_4_ production by comparing their production rates and cumulative release in the inoculated soil to that of the sterile inoculum control throughout the incubation period.

### Methods specific to the oxic experiment

#### Permafrost and inocula preparation

The permafrost soils were thawed overnight in a UV-treated and bleached positive pressure hood, then homogenized through an autoclaved 2 mm sieve into a stainless-steel bowl, and excess water was decanted. Approximately 20 g (fresh weight) homogenized soil was set in UV-treated 120 ml incubation flasks, sealed with Parafilm to allow for gas but not moisture or microorganism exchange, and pre-incubated at 10 °C for 11 days before inoculation. Samples of homogenized soil were taken to determine initial gravimetric water content, soil organic matter content (SOM), dissolved organic C (DOC), total dissolved N (TN), and pH (*n* = 4, except for Tussock-PF moisture and SOM where *n* = 3 due to low material amount).

We prepared microbial inoculum solutions by submerging active layer soils in autoclaved ultrapure water under a laminar flow hood. Soil slurries were prepared in autoclaved Erlenmeyer flasks with 50 g fresh weight active layer soil. The organic and mineral active layer soils differed in water holding capacity (SI Table S1), therefore, we used either 100 ml (“Cryoturbated-AL” and “Multifunctional” soils) or 150 ml (organic “Tussock-AL” and “Palsa-AL” soils) water. Slurries soaked for 1 h at 4 °C were shaken for 90 min (150 rpm, orbital), stored overnight at 4 °C, then filtered through qualitative filter paper (Ahlstrom-Munksjö, Eskilstuna, Sweden; 10 µm pore size, previously washed with autoclaved ultrapure water to prevent cellulose leaching). Three samples of each active layer soil were used to determine soil moisture, SOM, and pH. One milliliter of the respective soil suspensions was added to randomly assigned flasks for each of the four inoculum treatments (Cryoturbated-AL, Tussock-AL, Palsa-AL and Multifunctional), and one ml autoclaved ultrapure water was added to Control flasks. Putative artefacts due to the introduction of labile substrates such as microbial necromass were assessed in a separate incubation and found negligible (Supplementary Material).

#### Greenhouse gases measurements

To measure CO_2_ production rates, 10–15 ml headspace air was sampled with a syringe to measure CO_2_ concentrations with an infrared gas analyzer (EGM-5 IRGA, PP Systems, USA, static sampling mode) throughout the whole incubation period after production intervals ranging from 2 to 37 days (Supplementary Fig. S1), keeping [CO_2_]_v:v_ below 28,000 ppm. After measurement, the flasks were flushed with CO_2_-free air moisturized with a dew point generator (LI-COR Biosciences, Lincoln, Nebraska) and filtered through a 0.45 µm syringe filter for >90 s at 1 l min^−1^, i.e., with at least 10 times the volume of the headspace, to re-establish a near-zero headspace GHG concentration prior to the next measurement period. CO_2_ production rates (*τ*) were calculated as follows:

1 $${\tau }_{i}=\frac{{\left[{{\mathrm{CO}}}_{2}\right]}_{i}\cdot \frac{{P}_{i}\cdot V}{R\cdot T}}{{\Delta t}_{i}}$$

Where ${\tau }_{i}$ is the rate of CO_2_ production per time (net flux averaged over production interval), [CO_2_]_*i*_ is the CO_2_ concentration at measurement time i, Δ*t*_*i*_ is the duration of the production interval between measurement (_*i*_) and the previous flushing, *P*_*i*_ is atmospheric pressure at measurement time *i*, *V* the headspace volume of the incubated flasks, *R* the ideal gas constant and *T* the incubation temperature. Cumulative CO_2_ production over the entire 389-day period was obtained by summing up the quantity of CO_2_ produced in each flask across all measurements. Dissolved inorganic carbon was ignored in calculations but contributed to measured CO_2_ concentrations, as the flushing process primarily affects the gas phase. As no differences in pH were observed between treatments, this does not affect our interpretations but would have to be considered prior to upscaling or using our flux data quantitatively.

Concentrations of CH_4_ and N_2_O were measured at four time points during the incubation: after 7, 17, 40, and 187 days, to calculate the respective net production rates over the 5, 10, 17, and 7 days preceding these dates (Supplementary Fig. S1). On these days, 13 ml gas samples were taken with a syringe from the headspace—after sampling for CO_2_ but before flushing the headspace—and transferred to previously vacuumed 12 ml exetainer tubes (Labco, United Kingdom). The samples were shipped to INRAE facilities in Clermont-Ferrand and analyzed for CH_4_ and N_2_O using a gas chromatograph (GC, Clarus 480) equipped with a photoionization detector (PID, Vici 07709-S). N_2_O concentrations between 200 ppb v:v and our lowest technical reference gas of 400 ppb are not included in statistical analyses and only shown for reference, referred to as “trace amounts”. CH_4_ remained below the detection limit throughout the oxic incubation experiment.

#### Soil physical and chemical parameters

Soil chemistry variables—pH, water-extractable DOC, ammonium, nitrate, and TN—were measured on the homogenized permafrost soil prior to incubation, one day after inoculation, and at the end of the 389 days incubation. About 3 to 5 g fresh soil was sampled from the homogenized soils or from individual flasks and shaken for 2 h with 42 ml ultrapure water on an orbital shaker (150 rpm). The slurries were filtered through 10 µm qualitative filter paper, pH of the extracts was measured on a MP-150 pH-meter (Mettler Toledo, Switzerland), and the extracts were frozen at −20 °C until further analyses. DOC and TN of the extracts and of the inocula (see section 3.3.1 for inocula preparation) were analyzed on filtered and acidified aliquots (0.45 μm, Filtropur S, Sarstedt AG & Co., Germany; 50 μl 20% HCl to 20 ml filtrate) by high temperature catalytic oxidation using a Shimadzu TOC-V CPH analyzer with a TN unit (Shimadzu Corporation, Japan). Ammonium and nitrate were measured from the water extracts on a AA3 system (SEAL Analytical Ltd, United Kingdom). Water-extractable organic N was estimated by subtracting dissolved inorganic N (ammonia, nitrate and nitrite) from total water-extractable N. Soil moisture content (gravimetric) was determined by drying the soils at 105 °C for 48 h, and SOM by loss on ignition at 450 °C for 4 h. Soil physical and chemical variables prior to the beginning of the incubation for the four permafrost soils and the active layer soils used as inoculum are reported in Supplementary Table S5.

#### DNA extraction

Microcentrifuge tubes (1.5 ml) were filled with soil and snap-frozen in dry ice to analyze microbial communities from the flasks harvested after one day and at the end of the oxic incubation. Initial soils were sampled likewise, after thawing and homogenizing but before pre-incubation for the permafrost soils and before preparing soil suspensions for active layer soils. The frozen tubes were kept at −80 °C for 4 to 17 months until freeze-drying, then homogenized by bead-beating (Precellys CK-68 15 ml tubes, Bertin Technologies, France, 2 × 30 s 5000 rpm). Between 100 and 300 mg of homogenized freeze-dried soil were used for DNA extraction using DNEasy PowerSoil Pro Kit (Qiagen, Germany) according to the manufacturer’s instructions, and DNA concentrations were measured on a Qubit (ThermoFisher, USA) 1.0 fluorometer.

#### Bacterial amplicon sequencing and bioinformatics

The V4–V5 region of the 16S ribosomal RNA gene was PCR amplified using primers 515F^74^ and 926R^75^ with Illumina sequencing adapters, in a 2-step PCR procedure with conditions described in Supplementary Table S6. For a subset of samples from the Cryoturbated-PF soil, DNA concentrations were very low, and thus the extracts were used undiluted and/or with 30 amplification cycles to obtain products. Each pool of 96 PCR products included samples, negative controls (1–3 replicates of each DNA extraction- and PCR-blank), and a mock community as a positive control (2–5 replicates of ZymoBIOMICS Microbial Community DNA Standard, Zymo Research, USA). Two pools of 96 PCR products each were sequenced on Illumina MiSeq with V3 chemistry (2 × 300 bp, 15% PhiX spike-in) at the SNP&SEQ Technology Platform in Uppsala, where demultiplexing was performed by the sequencing platform.

Bioinformatics processing code is available at ref. ^76^. Amplicon sequence variants (ASVs) were created in R 4.5.2 with DADA2^77^ (pseudo-pooling) after removing non-target base pairs with cutadapt v5.0^78^. Taxonomy was assigned to ASVs with the RDP naïve Bayesian classifier^79^ as implemented in DADA2, and ASVs resolved to the genus rank were further assigned a species rank by the exact string-matching algorithm implemented in DADA2 (assignSpecies), with SILVA v138.1 reference data^80^. Putative contaminant ASVs were identified in silico using the decontam 1.28 package^81^. The very low DNA concentrations in some DNA extracts, particularly in the pre-incubation and day 1 extracts, would induce issues with the frequency-based method; therefore, only the prevalence-based method was used to identify contaminants, with a threshold of 0.15 (more stringent than the default recommended value of 0.1). Contaminant status of the ASVs was evaluated separately for each sequencing flow cell, and subsequently combined using default parameters (“minimum”), leading to 26 contaminant ASVs amounting up to 0.18% of the overall 14.3 million good-quality reads. Further ASV filtering was carried out so that mock community positive controls would be depicted as accurately as possible: ASVs present in fewer than 3 samples or amounting to fewer than 100 reads in total represented 8% of the reads and were removed. Samples with fewer than 20,000 reads were removed, resulting in a final ASV table of 13.1 million reads across 138 samples. Details of filtering steps, contaminant ASVs, and intermediate files are found in the code and data repository.

#### Functional genes assays

To determine the genetic potential and potential bottlenecks for different processes contributing to N_2_O production and reduction, qPCR of several functional genes were performed: the ammonia-monooxygenase *amoA* found in betaproteobacterial ammonia oxidizing bacteria^82^ and ammonia oxidizing archaea^83^ within the phylum Thermoproteota, nitrite-reductase genes in denitrification (*nirK*^84^ and *nirS*^85^), and nitrous oxide-reductase genes (*nosZ* clades I^84^ and II^86^), as well as the V4-V5 region of the bacterial 16S rRNA gene as a proxy for bacterial abundance^87^. DNA extracts were diluted to 1 ng µl^−1^ (or 1:8 for samples below 4 ng µl^−1^). Two 15 µl reactions per gene were analyzed on independent runs using CFX Connect or CFX-96 Real-Time System thermocyclers (Bio-Rad Laboratories, USA), and primers and conditions described in Supplementary Table S6, with touchdown conditions for *nirS*, *nirK*, and *nosZ* clade I. Each reaction contained 2 µl of diluted DNA template, 10 µg bovine serum albumin, 1x Biorad iQ SYBR Green Supermix (Bio-Rad Laboratories). The absence of polymerase inhibitors was ensured by amplifying a known amount of pCR 4-TOPO plasmid (Invitrogen, USA) added to the DNA extracts or no-template controls and comparing the threshold cycle number. No inhibition of the amplification reactions in any sample was detected with the amount of DNA used.

For samples where data were obtained for both *nir* and *nos* genes (Supplementary Table S7), we calculated the ratio of (*nirS* + *nirK*)/(*nosZ*I + *nosZ*II) as a proxy for potential N_2_O source vs. sink. Reliable quantitative data could not be obtained for bacterial *amoA* due to non-specific PCR amplification, and for the other genes, amplification could not always be obtained for all replicates (Supplementary Table S7). In the Cryoturbated-PF soil, 16S rRNA gene copy numbers were orders of magnitude lower than for other soils, and only one sample (out of 24) yielded functional gene copy numbers above detection limits; we therefore excluded Cryoturbated-PF from further analyses and discussion of qPCR data.

### Anoxic experiment

#### Permafrost and inocula preparation

The “Palsa-PF” permafrost was homogenized, decanted, and set into flasks as described above but in an anaerobic glovebox under N_2_ atmosphere. Soil moisture was measured using 5 g of homogenized soil (*n* = 4). Active layer soil suspensions were prepared using the active layer soil as described above, and compared a fresh soil inoculum (hereafter, “Palsa-AL”) and an inoculum prepared with gamma-irradiated (45 kGy) soil as a negative control (“Control”, see Supplementary Text for more detail). Soil was harvested at the end of the incubation for DNA extraction as described above, and then subjected to qPCR of *mcrA* and *pmoA* genes specific for methanogenesis and methane oxidation, respectively (Supplementary Table S6).

#### Greenhouse gases measurements

Net CO_2_, CH_4_, and N_2_O fluxes were measured and calculated as described above for CO_2_, except for using an Agilent 7890 GC coupled with a flame ionization detector (FID, CO_2_, and CH_4_) and an electron capture detector (ECD, N_2_O), and using moisturized N_2_ instead of CO_2_-free air for headspace flushing. N_2_O remained below the detection limit (100 ppb v:v) throughout the 175-day incubation. We estimated total warming potential by summing the mass of CO_2_ and CH_4_ converted into CO_2_-*eq*. We used the global warming potential at a 100-year horizon for non-fossil CH_4_ from the IPCC (GWP_100_, Table 7.15 in ref. ^88^) as a conversion factor, that is, multiplying grams of CH_4_ by 27.0.

#### DNA extractions and functional gene assays

In the anoxic experiment, DNA was extracted from permafrost soil in the pre-incubation (*n* = 6) and from samples at day 175 (*n* = 8) as described above for the oxic experiment, except for using fresh rather than freeze-dried samples. DNA extracts were diluted to reach about 1 ng µl^−1^ prior to qPCR. Quantitative PCR of genes coding for methyl-coenzyme M reductase (*mcrA*^89^) for methanogenesis and methane monooxygenase (*pmoA*^90,91^) for gammaproteobacterial methanotrophy was performed in 25 µl qPCR reactions in technical duplicates on a CFX Connect thermocycler with conditions according to Supplementary Table S2. The absence of inhibition was tested as described above for the oxic experiment with a pGEM plasmid, and no inhibition was detected. Reliable quantitative data could not be obtained for *pmoA* in incubated samples due to non-specific PCR amplification.

#### Statistical analyses

Statistical analyses were carried out in R 4.5.3^92^, and all data and code are available at ref. ^76^. While the effects of inoculation on daily rate of aerobic CO_2_ production were not necessarily stable through time, particularly in the first months (Supplementary Fig. S1), we decided to avoid speculative interpretations of these patterns due to the lack of supporting data at this temporal resolution. We therefore focused on cumulative CO_2_ production over the incubation and assessed how it was affected by inoculation treatments within each soil with one-way ANOVAs. Pairwise contrasts were computed when appropriate with the estimated marginal means method (emmeans 1.11.1 package and function^93^) and Holm adjustment for multiple comparisons.

We measured N_2_O at four out of 21 measurement dates (Supplementary Fig. S1), and since the headspace was flushed in between measurements, we could not calculate a cumulative budget for net N_2_O production in the same way as for CO_2_ (which was measured at all dates). Moreover, N_2_O was below the detection limit on most occasions; only the Palsa-PF soil at day 7 yielded enough data points for statistical testing. In this case, we accounted for unequal sample sizes and variances with a Kruskal–Wallis test on net N_2_O fluxes, using inoculum as a fixed factor.

Anaerobic CO_2_ and CH_4_ flux rates were analyzed with repeated-measures ANOVA (RM-ANOVA with the ez 4.4 package^94^), using time as within-subject variable and inoculum as between-subject variable. The time:inoculum interactions were not statistically significant (*P* > 0.3); therefore, cumulative CO_2_ and CH_4_ over the incubation, as well as the product of their conversion into CO_2-eq_ were compared between inoculum treatments using t-tests.

Differences in bacterial community composition between inoculation treatments for each soil type were assessed using manyglm models^95^ separately for day 1 and day 389, and pairwise comparisons were obtained using Holm *P* value adjustment with the wrapper provided in the mvabund 4.2.1 package^96^.

Within each soil type, the effects of the different inoculation treatments on most variables (functional gene abundances, TOC, TN, organic matter content, moisture, pH) were assessed with one-way ANOVAs using inoculation as a fixed factor, followed by pairwise contrasts when appropriate, using log-transformed data when necessary to meet ANOVA assumptions or Kruskal-Wallis tests when assumptions were not met (Supplementary Table S3). Since our hypotheses focused on treatment effects rather than temporal dynamics per se, separate ANOVAs were carried out one day after inoculation—to test for putative artefacts introduced by inoculation—and at the end of the incubation to test for functional consequences of inoculation.

We explored cross-dataset effects of community-functioning relationships by focusing on CO_2_, as this variable was available for all samples. To identify the best descriptors of CO_2_ production dynamics among 107 soil chemistry and bacterial community variables, we used a machine learning regression approach (Random Forests), essentially comparing model fits in the presence or absence of each variable to compare their explanatory power. We focused on relative differences in cumulative CO_2_ production at day 389 within a given soil; i.e., cumulative CO_2_ per gram dry soil was detrended by dividing each value by the average for its soil type—allowing us to explore the variation caused by treatments while ignoring the intrinsic variation between soils. Class-level taxonomy was the best compromise between interpretability and representativeness, and therefore we used all 94 bacterial classes identified throughout the dataset. Soil chemistry variables were soil moisture, organic matter, DOC, DIN, ammonia, nitrite+nitrate, and ON content, as well as pH, with dissolved variables expressed both per soil dry weight and per soil organic matter weight. This resulted in 107 variables for 58 data points. We used Random forests^97^, a regression tree machine learning approach well-suited to cases where the number of variables far exceeds the number of observations. We used two implementations of Random forests designed to select variables among large numbers of collinear variables: Variable Selection Using Random Forest (VSURF 1.2.1^98,99^) and rfPermute 2.5.5^100^. In both cases, the number of variables in each tree was kept at the default value (mtry, 1/3 of all variables), and variables were scaled; for VSURF, we increased the number of forests at the thresholding and interpretation steps to 50; for rfPermute, we increased the number of permutation replicates to 10,000. We retained only variables selected at the interpretation step of VSURF and for which both metrics (increase in mean squared error and node purity) significantly affected importance in rfPermute (*P* < 0.05). We then tested the correlation between these variables and CO_2_ production detrended within each soil, and focused the discussion on those which significantly correlated with CO_2_ in all soils.

### Reporting summary

Further information on research design is available in the Nature Portfolio Reporting Summary linked to this article.

## Supplementary information

Supplementary Information

Reporting Summary

Transparent Peer Review File

## Source data

Source Data

## Data Availability

The data generated in this study are available at the Bolin Centre Database under project monteux-2026-permafrost-inoculations [https://doi.org/10.57669/monteux-2026-permafrost-inoculation-1.1.0]. The sequencing data generated in this study have been deposited in the ENA under accession code [https://www.ebi.ac.uk/ena/browser/view/PRJEB97802]. The cumulative CO_2_, CH_4_, inorganic nitrogen, net N_2_O production rate, bacterial community composition, and bacterial alpha diversity are further provided as Source Data. Source data are provided with this paper.

## Code availability

All codes used to produce the findings presented in this study are available at https://doi.org/10.57669/monteux-2026-permafrost-inoculation-1.1.0.
