# Supplementary material for: Active layer microbial inocula restore missing functions across thawed permafrost soils
Source: Nat Commun. 2026 Sep 25;17:10230. doi: 10.1038/s41467-026-78060-4 (PMC13614969; doi:10.1038/s41467-026-78060-4)
Supplement: Supplementary file 1 [file 41467_2026_78060_MOESM1_ESM.pdf]

Active layer microbial inocula restore missing functions across thawed permafrost soils

Supplementary Information

Sylvain Monteux[1,2,3,4], Ellen Dorrepaal[5], Sébastien Fontaine[6], Konstantin Gavazov[7], Sara Hallin[8,9], Jaanis Juhanson[8], Frida Keuper[10], Alexander T. Tveit[11], Josefine Walz[5], Rica Wegner[2], Birgit Wild[2,3], Eveline J. Krab[1]

1: Department of Soil and Environment, Swedish University of Agricultural Sciences, Uppsala, Sweden
2: Department of Environmental Science, Stockholm University, Stockholm, Sweden
3: Bolin Centre for Climate Research, Stockholm University, Stockholm, Sweden
4: Tromsø Museum, UiT The Arctic University of Norway, Tromsø, Norway
5: Climate Impacts Research Centre, Umeå University, Abisko, Sweden
6: Grassland Ecosystem Research Unit, French National Institute for Food, Agriculture and Environment INRAE, Clermont-Ferrand, France
7: Swiss Federal Institute for Forest, Snow and Landscape Research WSL, Birmensdorf, Switzerland
8: Department of Forest Mycology and Plant Pathology, Swedish University of Agricultural Sciences, Uppsala, Sweden
9: Science for Life Laboratory, Swedish University for Agricultural Sciences, Uppsala, Sweden
10: BioEcoAgro Joint Cross-Border Research Unit, French National Institute for Food, Agriculture and Environment INRAE, Laon, France
11: Department of Arctic and Marine Biology, UiT The Arctic University of Norway, Tromsø, Norway

Table of contents

- Supplementary Methods
- Supplementary Figures
- Supplementary Tables

Supplementary Methods

Complementary oxic incubation: inoculum artefacts

Study design

The introduction of labile substrates, such as those derived from dead microbial cells, might be increased by the addition of 1 ml soil suspensions used as inoculum in active layer and positive control treatments. We tested this using the Yedoma sediment where functional limitations were known, by incubating two sets of jars mimicking the negative and positive controls used above (‘Control’ and ‘Live’, respectively), and one set where the inoculum consisted in the same soil used for positive controls but that had undergone gamma-irradiation at 45 kGy (‘Sterile’). The jars were incubated for 389 days at 10 °C under dark oxic conditions in 4 replicates, and CO_2 production was monitored as described for the main experiment. The entire design was duplicated, using in one case liquid inoculum as in the rest of the manuscript, and in the second case a solid soil transfer as in ref. [10].

The CO_2 flux data was analysed with RM-ANOVA for solid inoculum, and no interaction between inoculation treatment and time was detected ($P = 0.225$). The loss of one jar during the incubation unbalanced the design for liquid inoculum, thus we used a two-way ANOVA on cumulative emissions at day 356, using inoculum type (liquid or solid) and inoculum treatment (control, sterile or positive control) as fixed factors, followed by appropriate pairwise contrasts with *emmeans*.

Results

The inoculation method (i.e. liquid or solid) significantly interacted with the type of inoculum (control, gamma-irradiated or live positive control soil, Supplementary Figure S7). CO_2 production in the live positive control was significantly higher than in both the control and gamma-irradiated soil inoculum, but this increase was less pronounced when using a solid inoculum (+31% ± 2% compared to negative control, estimated marginal mean ± SE, n = 4) than a liquid inoculum (+61% ± 3%). The CO_2 production in soils inoculated with gamma-irradiated inoculum in either liquid or solid form did not differ from the negative control ($P \geq 0.735$).

Anoxic incubation: control on inoculum artefacts

To circumvent putative biases caused by introduced substrates upon inoculation, the experimental design of the anoxic incubation accounted for such biases. Instead of using ultrapure water as a negative control and a filtered solution of active layer soil suspension for inoculation, we used an active layer soil suspension in both cases. Soil from the active layer was separated into two flasks, once of which was shipped to Scandinavian Clinics Estonia for sterilization by gamma-irradiation at 45 kGy. While gamma-irradiation inevitably leads to modifications in soil chemistry, it is the least damaging solution for sterilization.

Permafrost soil was thawed at room temperature in an N_2-atmosphere. The excess water after thawing was decanted and filtered (8 µm Whatman grade 40 paper filter) into an autoclaved glass bottle: this constitutes suspension (1). *Soil slurries were created from both the sterilized ('Control') and the live active layer soil ('Palsa'), by putting 1 g of soil into 70 ml of suspension (1), shaking (150 rpm orbital shaker 90 min) and soaking for at least 2 hours at room temperature, before filtering (8 µm Whatman grade 40).* Control and Palsa suspensions were stored at 4 °C overnight, before adding 2 ml of those to half of the 16 jars containing 15 g of homogenized permafrost, selected at random. All steps were performed using autoclaved material, steps in italic font were not performed under a N_2-atmosphere but using sterile technique.

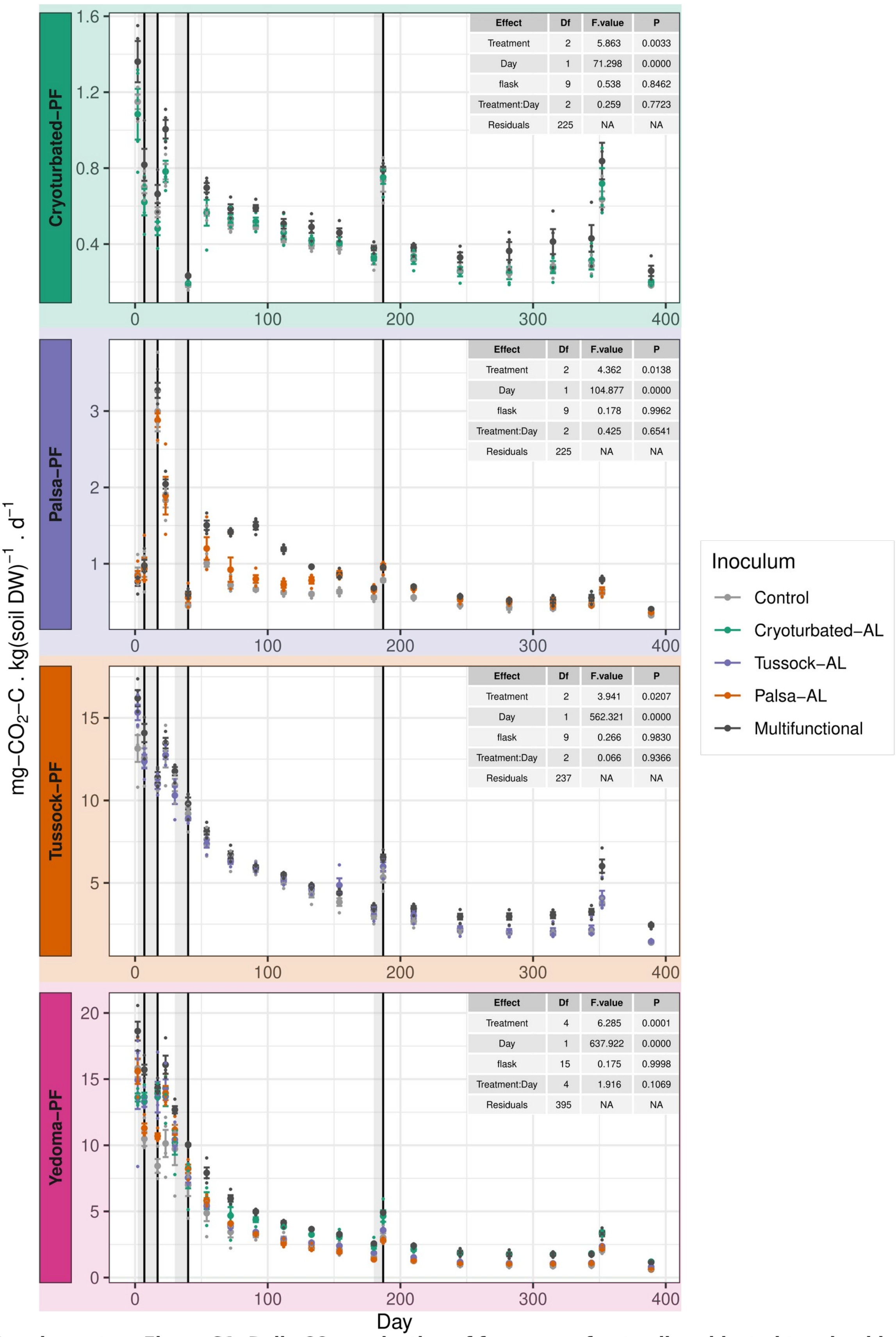

Supplementary Figure S1: Daily CO_2 production of four permafrost soils subjected to microbial community manipulation over 389 days of oxic incubation. Large symbols and error-bars indicate mean +/- SE (n=4), small symbols are individual measurements. Vertical black bars indicate sampling dates for CH_4 and N_2O, with greyed out sections depicting the period over which the corresponding net emission rates are calculated. Table insets show results of ANOVA on linear models used to assess inoculum:time (Treatment:Day) interactions prior to testing differences in cumulative CO_2 release. Higher values at days 187 and 352 are presumably due to the shorter time interval between measurements, giving a disproportionately high role to the diffusion from pore space to CO_2-free headspace immediately after flushing. Conversely, lower values at day 40 for Turbel and Palsa soils are presumably due to the longer time interval between measurements.

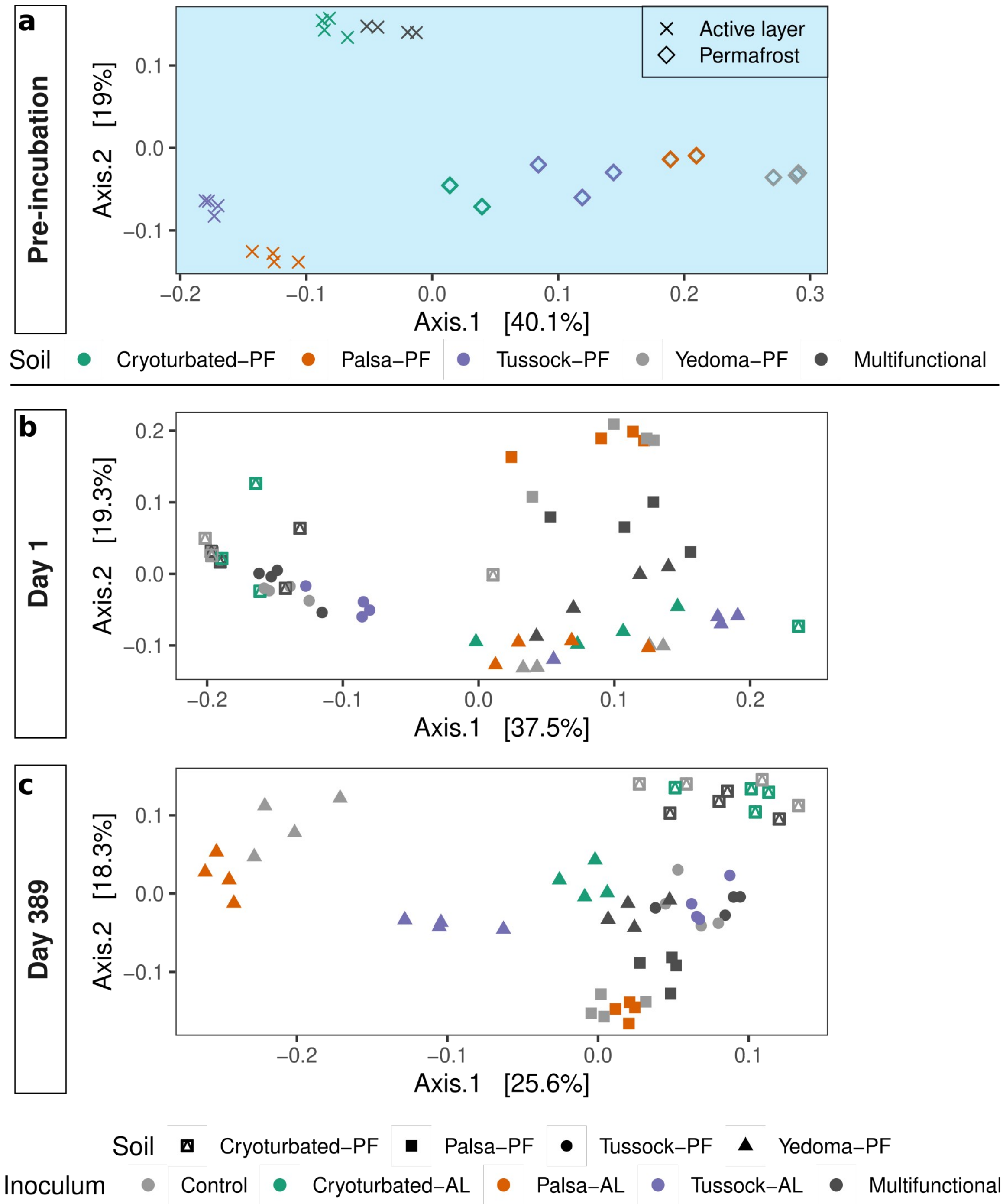

Supplementary Figure S2: Bacterial communities of four permafrost soils subjected to microbial community manipulation before (a), after 1 day (b) and after 389 days (c) of oxic incubation.
Principal coordinate analyses (PCoA) of weighted UniFrac distances of amplicon sequence variants (ASVs) computed from amplicons of 16S rRNA gene V4-V5 region.
(a) Pre-incubation permafrost and active layer samples, permafrost samples were processed in triplicates and samples failed sequencing quality control for Palsa and Cryoturbated resulting in n=2.
(b,c) Incubated jars after one day and 389 days of aerobic incubation, n=4.

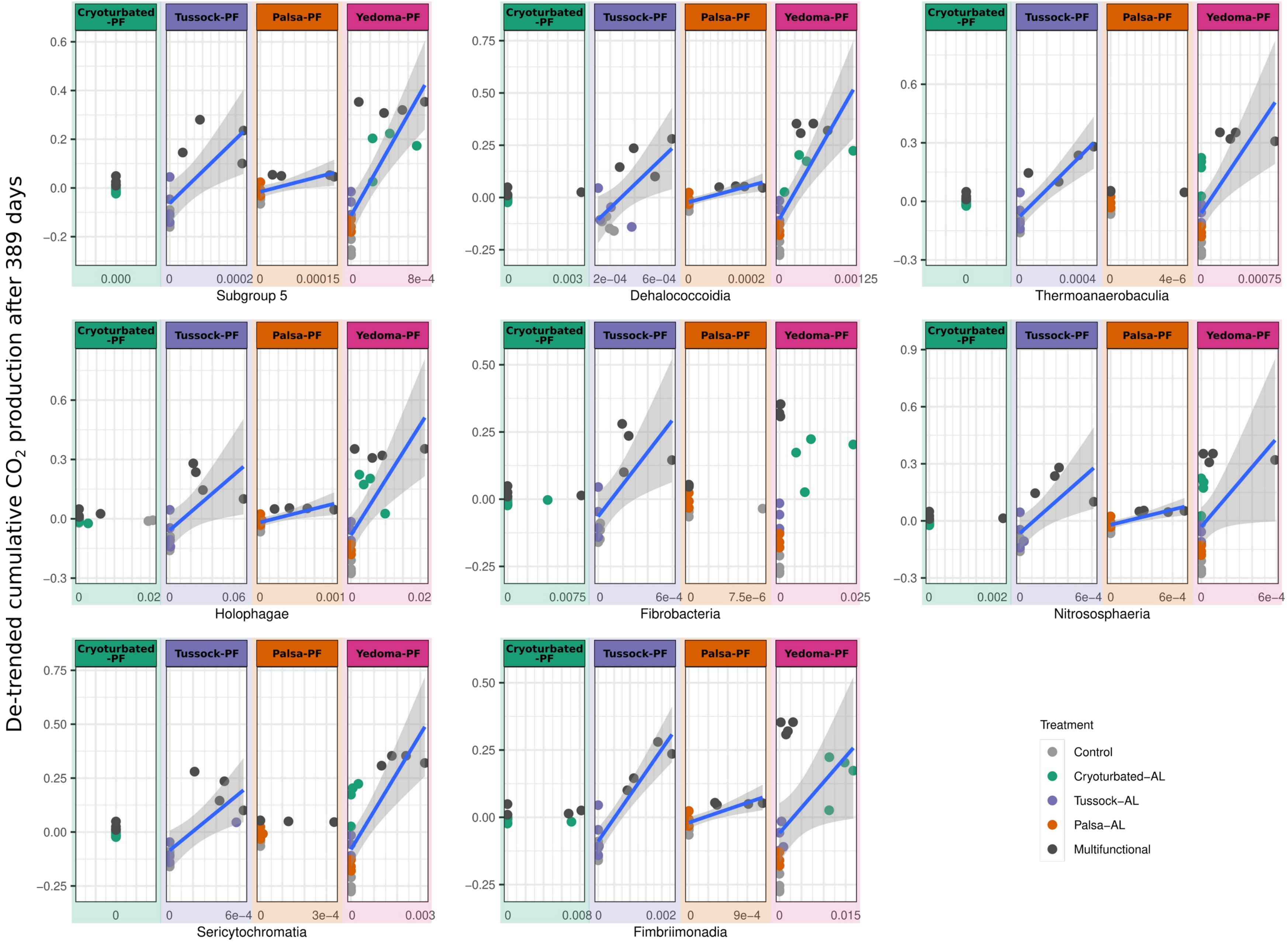

Supplementary Figure S3: Relationship between cumulative CO_2 production and the variables identified as important in explaining the variation in CO_2 production by RandomForests, in four permafrost types subjected to microbial community manipulation after 389 days of oxic incubation. All variables retained by VSURF and rfPermute were bacterial classes and not soil chemistry variables. X-axes indicate the relative abundance of each class in each sample, Y-axes represent cumulative CO_2 production, de-trended within each soil to focus the analysis on within- rather than between-soil variance. Lines and 95% confidence intervals are only shown where the two variables are correlated (linear model fit $P < 0.05$). Red rectangles highlight variables with consistent relationships across soil types (excepted Cryoturbated where DNA quality was suboptimal).

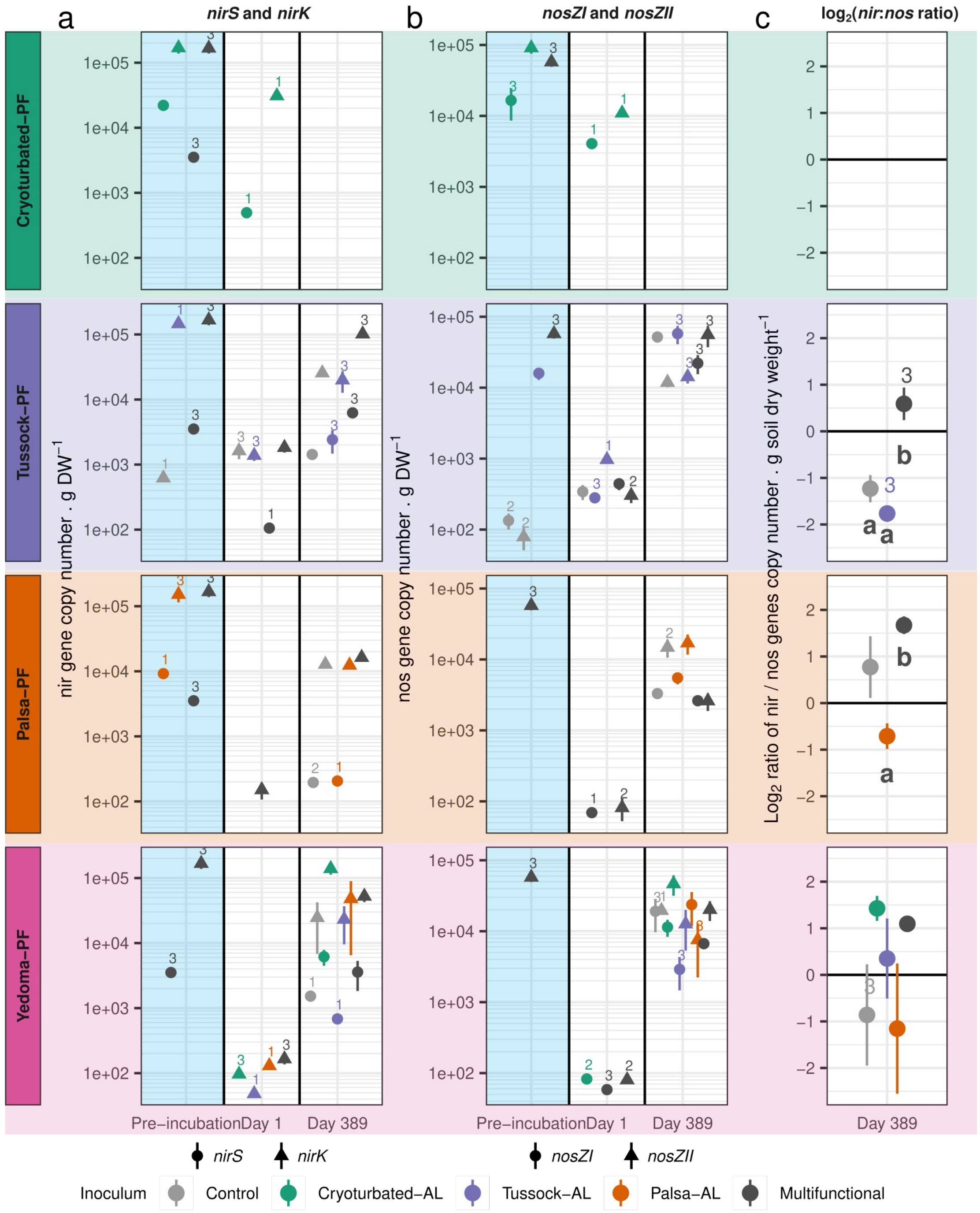

Supplementary Figure S4: Abundance of variants of genes controlling production (nitrite reductase, a) and consumption (nitrous oxide reductase, b) of N_2O as well as their ratio in four permafrost soils subjected to microbial community manipulation after 389 days of oxic incubation.

(**a-b**) gene copy numbers per gram soil dry weight, (**c**) $\log_2$ of the sum of *nirS* and *nirK* divided by sum of *nosZ* clade I and II gene copy numbers expressed per gram soil dry weight. Symbols and error bars depict mean +/- SE (n=4 unless specified otherwise by numbers when below detection limit). In (**c**) horizontal black lines indicate a $\log_2$ ratio of 0, i.e. a ratio of 1 (*nir* = *nos*); ratios computed only where both *nir* and *nos* genes were detected. Different letters denote significant differences within a soil type (EMmeans Holm-adjusted $P < 0.05$).

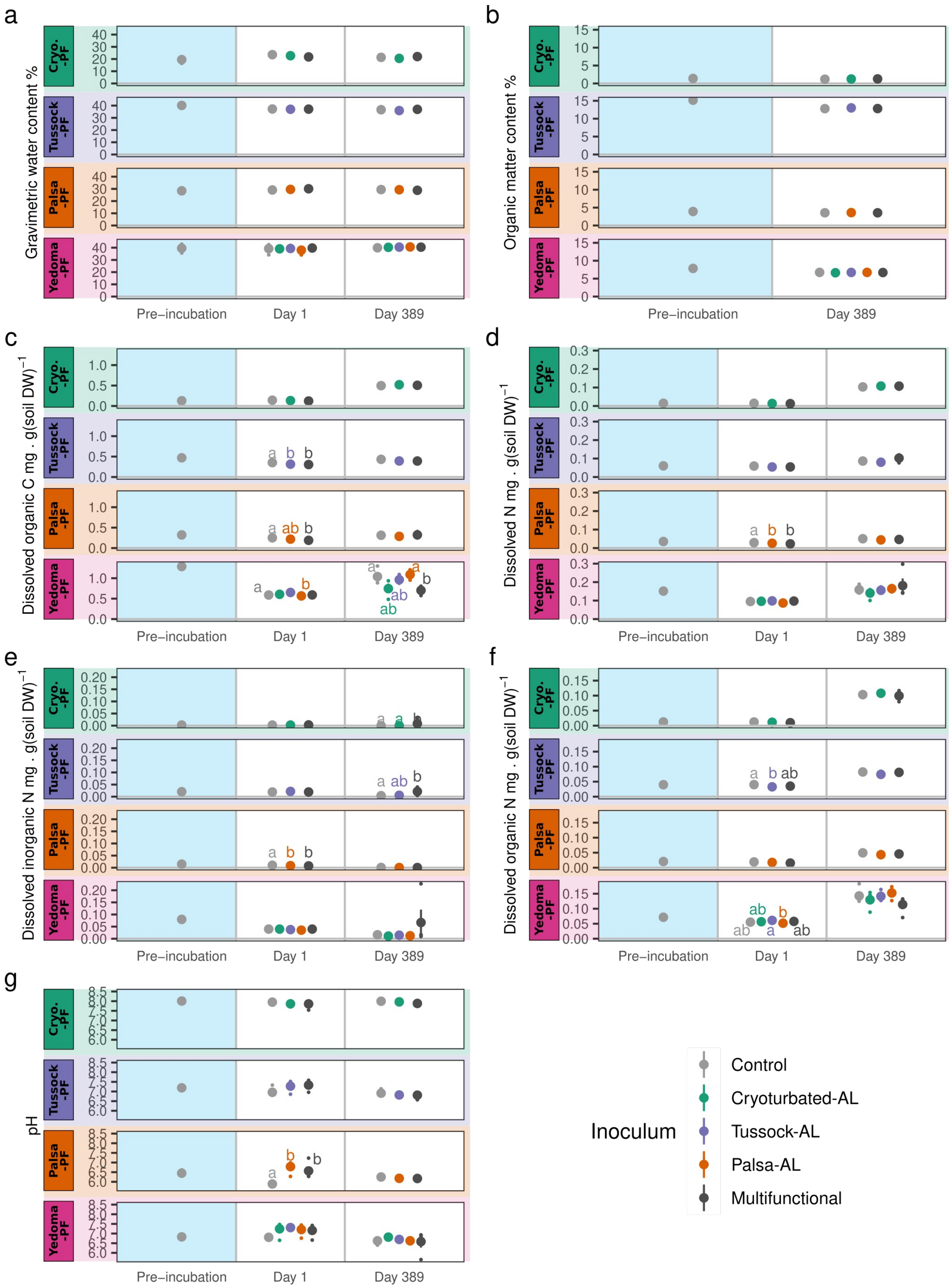

Supplementary Figure S5: Physical and chemical variables of four permafrost soils subjected to microbial community manipulation over 389 days of oxic incubation. (**a**) Gravimetric water content, (**b**) organic matter content, (**c**) water-extractable dissolved organic C, (**d**) water-extractable dissolved nitrogen. (**e**) dissolved inorganic nitrogen content, (**f**) dissolved organic nitrogen content, (**g**) pH. Large symbols and error-bars represent mean +/- SE (n=4), small symbols represent individual measurements, different letters denote statistically significant differences within a soil:date group (Holm-adjusted $P < 0.05$). Organic matter content was not measured at day 1 in (**b**), as it was presumedly unchanged from pre-incubation values.

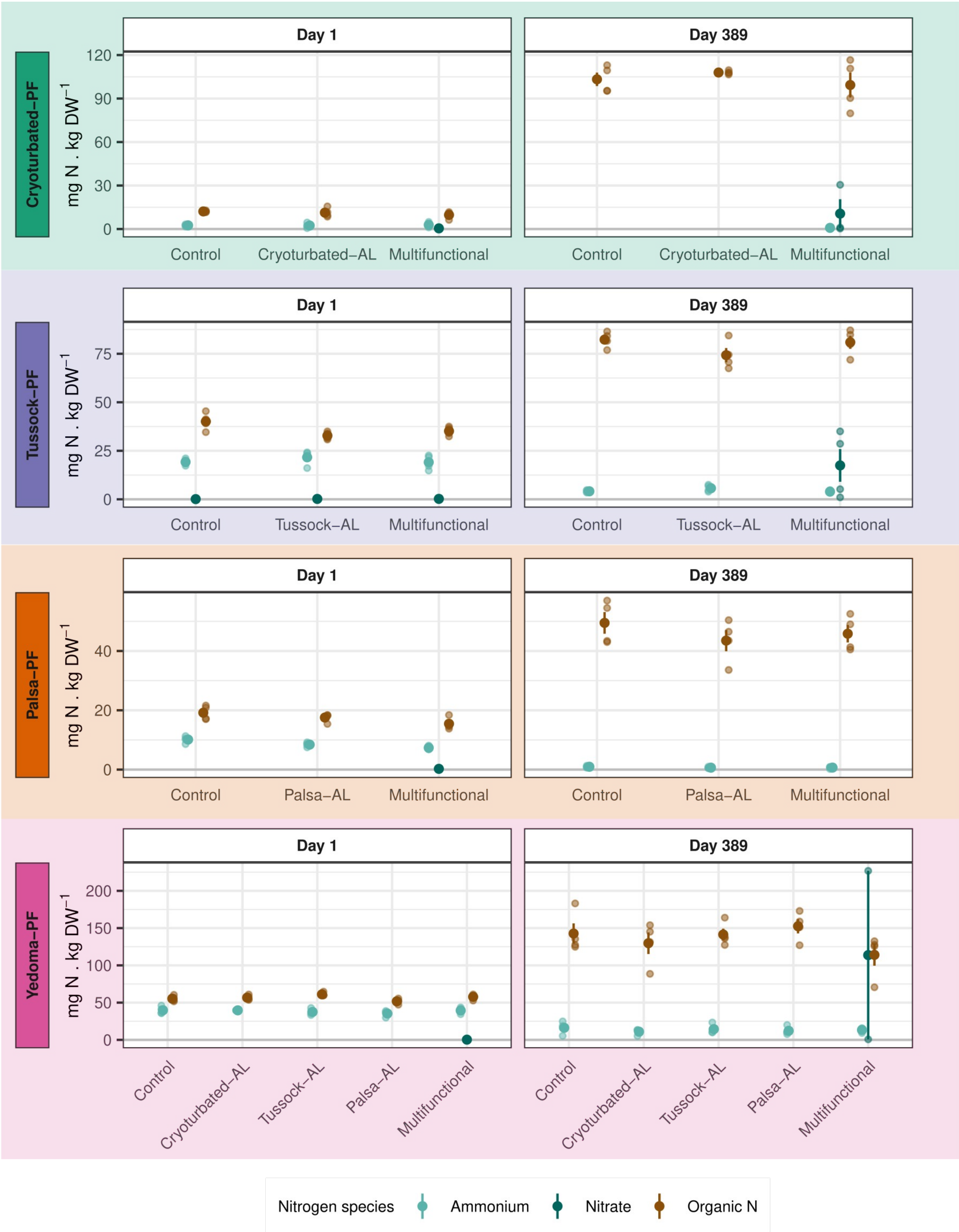

Supplementary Figure S6: Distribution of water-extractable nitrogen species in four permafrost soils subjected to microbial community manipulation over 389 days of oxic incubation. Points represent individual values for each sample, bars depict the same values added to each other for comparison of the relative contributions to the total dissolved N pool. Large symbols represent mean±SE (n = 4), small symbols represent invidivual measurements.

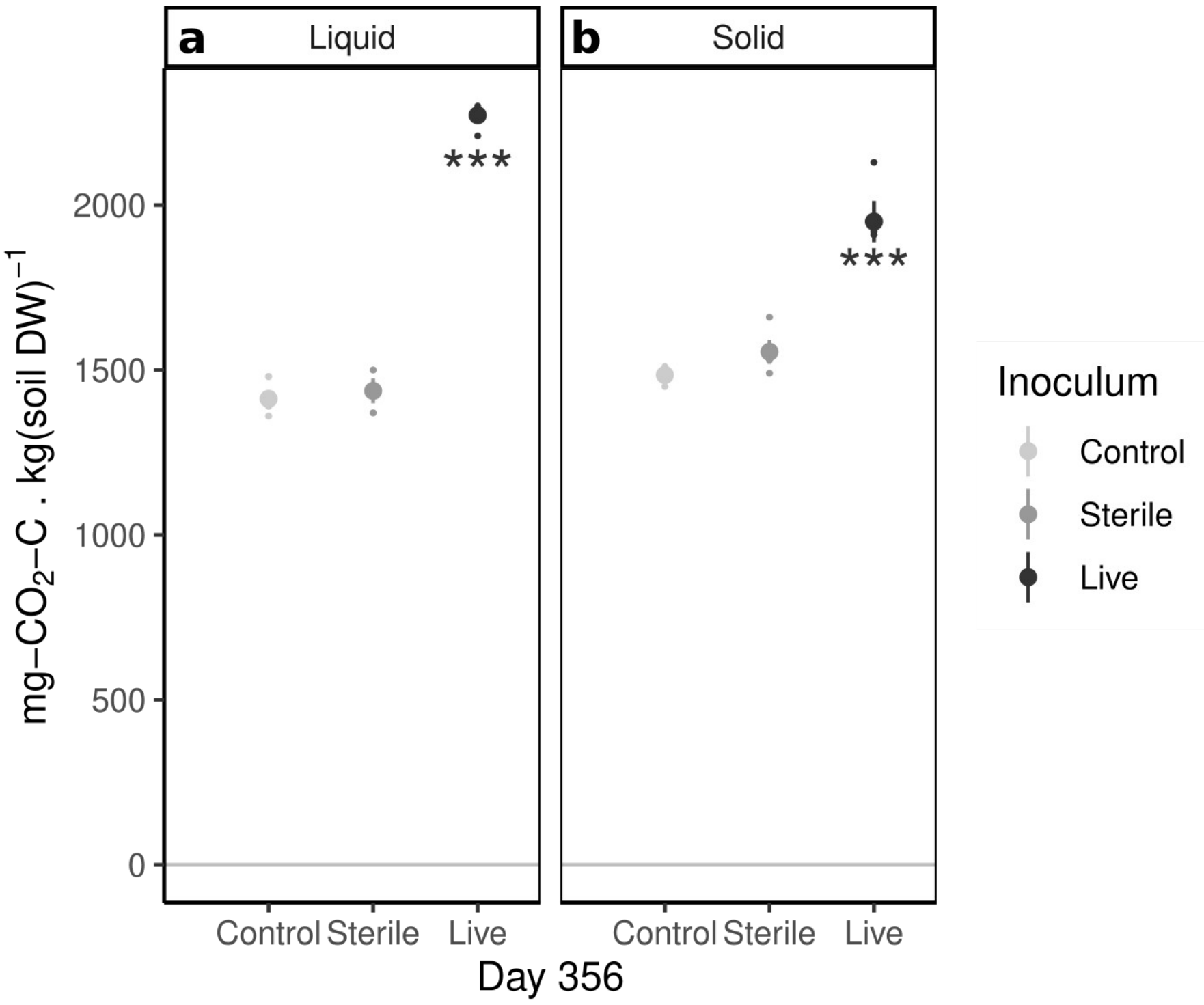

Supplementary Figure S7: Supplementary oxic incubation of Yedoma permafrost to assess artefacts from inoculation.
(a-b): Cumulative CO_2 production of Yedoma permafrost inoculated with the positive control soil (Live) or gamma-irradiated positive control soil (Sterile), after 356 days of oxic incubation. The two panels differ in inoculation method, either with **(a)** 1ml of soil suspension (ddH_2O for Control), or with **(b)** 1.5% weight:weight soil replacement (as in Monteux *et al*., 2020 ref [10]). Symbols and error-bars indicate mean +/- SE (n=4, except Liquid-Sterile where n=3), asterisks denote significant (Holm-adjusted $P < 10^{-4}$) differences from the Control.

Supplementary Table S1: Effects of inoculation on bacterial community composition in four permafrost soils (ManyGLM ANOVA with pairwise comparisons). Values in bold indicate significant (Holm free stepdown adjusted P<0.05) tests, n=4.

		Day 1			Day 389		
	**Soil type**	**Dev**	**df**	***P***	**Dev**	**df**	***P***
**ManyGLM ANOVA**	*Cryoturbated-PF*	3900	2	0.131	1936	2	0.064
	Tussock-PF	15779	2	**0.005**	29951	2	**0.002**
	Palsa-PF	24140	2	**0.002**	23226	2	**0.004**
	Yedoma-PF	15785	4	**0.003**	36970	4	**0.001**
**Pairwise comparisons**	**Contrast**	**Observed statistic**		***P***	**Observed statistic**		***P***
Cryoturbated-PF	*Cryoturbated-AL vs Multifunctional*	2332		0.242	1000		0.255
	Control vs Cryoturbated-AL	2038		0.242	873		0.255
	Control vs Multifunctional	796		0.242	793		0.255
Tussock-PF	*Tussock-AL vs Multifunctional*	10725		**0.02**	21678		**0.018**
	Control vs Tussock-AL	1873		0.353	3026		0.253
	Control vs Multifunctional	10686		**0.02**	21163		**0.018**
Palsa-PF	*Palsa-AL vs Multifunctional*	17365		**0.022**	16103		**0.019**
	Control vs Palsa-AL	2795		0.079	3204		**0.048**
	Control vs Multifunctional	16050		**0.022**	14871		**0.019**
Yedoma-PF	*Control vs Cryoturbated-AL*	2155		0.193	9270		**0.030**
	Control vs Tussock-AL	681		0.193	3043		**0.030**
	Control vs Palsa-AL	1482		0.193	1664		**0.030**
	Control vs Multifunctional	5213		**0.047**	13805		**0.030**
	Cryoturbated-AL vs Tussock-AL	2343		0.193	9912		**0.030**
	Cryoturbated-AL vs Palsa-AL	2568		0.193	9894		**0.030**
	Cryoturbated-AL vs Multifunctional	6179		**0.047**	14599		**0.030**
	Tussock-AL vs Palsa-AL	1669		0.193	3068		**0.030**
	Tussock-AL vs Multifunctional	5229		**0.047**	13974		**0.030**
	Palsa-AL vs Multifunctional	6092		**0.047**	14206		**0.030**

Supplementary Table S2: Effects of inoculation on bacterial alpha-diversity and on the abundance of 16S rRNA, *nir* and *nosZ* genes in four permafrost soils.
Values in bold indicate significant (Holm-adjusted P<0.05) tests, n=4.

			Day 1			Day 389		
**ANOVA**	**Soil**		**F**	**df**	***P***	**F**	**df**	***P***
**Alpha-diversity Coverage Estimator**	*Cryoturbated-PF*		3.727†	2	0.155	0.4003	2	0.683
	Tussock-PF		70.982	2	**<0.001**	63.855	2	**<0.001**
	Palsa-PF		21.787	2	**<0.001**	44.464	2	**<0.001**
	Yedoma-PF		5.025	4	**0.009**	89.624	4	**<0.001**
**16S rRNA gene copy number**	*Cryoturbated-PF*			N.A.			N.A.	
	Tussock-PF		0.078	2	0.926	0.439	2	0.661
	Palsa-PF		5.854	2	**0.024**	2.4215	2	0.144
	Yedoma-PF		1.083	4	0.400	15.68	4	**<0.001**
**Sum of *nirS* and *nirK***	*Cryoturbated-PF*			N.A.			N.A.	
	Tussock-PF		0.472	2	0.642	20.655	2	**0.001**
	Palsa-PF			N.A.		1.63	2	0.249
	Yedoma-PF			N.A.		3.383	2	**0.037**
**Sum of *nosZ*-I and *nosZ*-II**	*Cryoturbated-PF*			N.A.			N.A.	
	Tussock-PF		0.608	2	0.568	0.095	2	0.911
	Palsa-PF			N.A.		3.764	2	0.065
	Yedoma-PF			N.A.		2.936	2	0.059
**Estimated marginal means**	**Soil**	**Contrast**	**t-ratio**	**df**	**P**	**t-ratio**	**df**	**P**
**Alpha-diversity Coverage Estimator**	*Tussock-PF*	*Control – Tussock AL*	0.47	9	0.649	0.30	9	0.768
		Control – Multifunctional	-10.08	9	**6.9.10^{-6}**	-9.63	9	**1.1.10^{-5}**
		Multifunctional – Tussock AL	10.55	9	**6.9.10^{-6}**	9.94	9	**1.1.10^{-5}**
	Palsa-PF	*Control – Palsa AL*	-2.61	9	**0.028**	-2.81	9	**0.020**
		Control – Multifunctional	-6.56	9	**3.0.10^{-4}**	-9.20	9	**2.1.10^{-5}**
		Multifunctional – Palsa AL	3.95	9	**0.007**	6.39	9	**2.5.10^{-4}**
	Yedoma-PF	*Control – Cryoturbated AL*	-2.59	15	0.164	-12.62	15	**1.7.10^{-8}**
		Control – Multifunctional	-3.73	15	**0.020**	-16.41	15	**5.4.10^{-10}**
		Control – Palsa AL	-2.16	15	0.283	-3.44	15	**0.007**
		Control – Tussock AL	-0.27	15	1.000	-6.40	15	**6.0.10^{-5}**
		Cryoturbated AL – Multifunctional	-1.14	15	0.815	-3.79	15	**0.005**
		Cryoturbated AL – Palsa AL	0.43	15	1.000	9.18	15	**9.1.10^{-7}**
		Cryoturbated AL – Tussock AL	2.32	15	0.244	6.22	15	**6.5.10^{-5}**
		Multifunctional – Palsa AL	1.57	15	0.551	12.97	15	**1.3.10^{-8}**
		Multifunctional – Tussock AL	3.46	15	**0.031**	10.01	15	**3.4.10^{-7}**
		Palsa AL – Tussock AL	1.89	15	0.389	-2.96	15	**0.010**
**16S rRNA gene copy number**	*Palsa-PF*	*Control – Palsa AL*	-0.53	9	0.606		N.A.	
		Control – Multifunctional	-3.19	9	**0.033**			
		Palsa AL – Multifunctional	-2.66	9	0.052			
	Yedoma-PF	*Control – Cryoturbated AL*		N.A.		2.868	15.000	0.070
		Control – Tussock AL				2.180	15.000	0.228
		Control – Palsa AL				1.812	15.000	0.360
		Control – Multifunctional				7.510	15.000	**1.9.10^{-5}**
		Cryoturbated AL – Tussock AL				-0.687	15.000	1.000
		Cryoturbated AL – Palsa AL				-1.056	15.000	0.924
		Cryoturbated AL – Multifunctional				4.642	15.000	**0.002**
		Tussock AL – Palsa AL				-0.368	15.000	1.000
		Tussock AL – Multifunctional				5.330	15.000	**0.001**
		Palsa AL – Multifunctional				5.698	15.000	**4.10^{-4}**
**Sum of *nirS* and *nirK***	*Tussock-PF*	*Control – Tussock AL*		N.A.		1.110	7.000	0.304
		Control – Multifunctional				-5.244	7.000	**0.002**
		Multifunctional – Tussock AL				5.944	7.000	**0.002**
	Yedoma-PF	*Control – Cryoturbated AL*		N.A.		-2.805	15.000	0.120
		Control – Tussock AL				-0.204	15.000	1.000
		Control – Palsa AL				0.211	15.000	1.000
		Control – Multifunctional				-1.680	15.000	0.682
		Cryoturbated AL – Tussock AL				2.601	15.000	0.161
		Cryoturbated AL – Palsa AL				3.016	15.000	0.087
		Cryoturbated AL – Multifunctional				1.125	15.000	1.000
		Tussock AL – Palsa AL				0.415	15.000	1.000
		Tussock AL – Multifunctional				-1.476	15.000	0.803
		Palsa AL – Multifunctional				-1.891	15.000	0.547

Supplementary Table S3: Analyses of variance (ANOVA) of inoculation effect on biogeochemical variables in four permafrost soils subjected to microbial community manipulation, and pairwise post-hoc comparisons where relevant (estimated marginal means). Values in bold indicate significant (Holm-adjusted $P < 0.05$) tests, n=4, except for Ammonium-Yedoma-PF-Day389 where n=3. †: Kruskal-Wallis X^2.

			Day 1			Day 389		
**ANOVA**		**Soil**	**F**	**df**	**P**	**F**	**df**	**P**
	**Cumulative CO_2 production**	*Cryoturbated-PF*				11.647	2	**0.003**
		Tussock-PF		N.A.		23.59	2	**$2.6.10^{-4}$**
		Palsa-PF				36.844	2	**$4.6.10^{-5}$**
		Yedoma-PF				92.945	4	**$2.1.10^{-10}$**
	**Gravimetric water content**	*Cryoturbated-PF*	2.246	2	0.162	1.759	2	0.227
		Tussock-PF	0.335†	2	0.846	1.841	2	0.214
		Palsa-PF	3.204†	2	0.202	0.281	2	0.761
		Yedoma-PF	0.341	4	0.846	0.259	4	0.900
	**Organic matter content**	*Cryoturbated-PF*				1.197	2	0.346
		Tussock-PF		N.A.		0.597	2	0.571
		Palsa-PF				0.294	2	0.752
		Yedoma-PF				0.224	4	0.921
	**Dissolved organic carbon**	*Cryoturbated-PF*	0.658	2	0.541	0.389	2	0.689
		Tussock-PF	8.031	2	**0.010**	1.784	2	0.223
		Palsa-PF	11.773	2	**0.003**	0.565	2	0.587
		Yedoma-PF	3.772	4	**0.026**	6.236	4	**0.004**
	**Total dissolved nitrogen**	*Cryoturbated-PF*	1.7	2	0.236	0.39	2	0.688
		Tussock-PF	2.387	2	0.147	3.471	2	0.076
		Palsa-PF	12.926	2	**0.002**	0.864	2	0.454
		Yedoma-PF	2.727	4	0.069	2.992†	4	0.559
	**Ammonium**	*Cryoturbated-PF*	0.1085	2	0.898		N.A.	
		Tussock-PF	0.802	2	0.478	3.052	2	0.097
		Palsa-PF	11.214	2	**0.004**	1.334	2	0.311
		Yedoma-PF	1.054	4	0.413	0.65	4	0.636
	**Dissolved inorganic nitrogen**	*Cryoturbated-PF*	0.207	2	0.817	7.2†	2	**0.027**
		Tussock-PF	2.102†	2	0.35	6.362†	2	**0.042**
		Palsa-PF	10.883	2	**0.004**	1.334	2	0.311
		Yedoma-PF	1.123	4	0.383	2.514†	4	0.642
	**pH**	*Cryoturbated-PF*	0.447	2	0.653	3.343	2	0.082
		Tussock-PF	2.285	2	0.158	0.664	2	0.538
		Palsa-PF	8.054	2	**0.010**	2.262	2	0.16
		Yedoma-PF	4.807†	4	0.308	6.739†	4	0.15
	**Organic nitrogen**	*Cryoturbated-PF*	1.011	2	0.374	0.038†	2	0.981
		Tussock-PF	5.669	2	**0.026**	1.907	2	0.204
		Palsa-PF	3.485	2	0.076	0.78	2	0.487
		Yedoma-PF	4.252	4	**0.017**	1.418	4	0.276
**Estimated marginal means**	**Soil**	**Contrast**	**t-ratio**	**df**	**P**	**t-ratio**	**df**	**P**
**Cumulative CO_2 production**		*Control – Cryoturbated AL*				-0.36	9	0.729
	Cryoturbated-PF	*Control – Multifunctional*		N.A.		-4.35	9	0.006
		Cryoturbated AL – Multifunctional				-3.99	9	0.006
		Control – Tussock AL				-1.35	9	0.210
	Tussock-PF	*Control – Multifunctional*		N.A.		-6.51	9	0.000
		Tussock AL – Multifunctional				-5.16	9	0.001
		Control – Palsa AL				-3.47	9	0.007
	Palsa-PF	*Control – Multifunctional*		N.A.		-8.54	9	$4.0.10^{-5}$
		Palsa AL – Multifunctional				-5.06	9	0.001
		Control – Cryoturbated AL				-11.58	15	$5.4.10^{-8}$
		Control – Tussock AL				-4.98	15	$6.1.10^{-4}$
		Control – Palsa AL				-2.55	15	0.044
		Control – Multifunctional				-16.60	15	$4.6.10^{-10}$
	Yedoma-PF	*Cryoturbated AL – Tussock AL*		N.A.		6.60	15	$4.2.10^{-5}$
		Cryoturbated AL – Palsa AL				9.03	15	$1.1.10^{-6}$
		Cryoturbated AL- Multifunctional				-5.02	15	0.001
		Tussock AL – Palsa AL				2.42	15	0.044
		Tussock AL – Multifunctional				-11.62	15	$5.4.10^{-5}$
		Palsa AL – Multifunctional				-14.05	15	$4.4.10^{-9}$

Supplementary Table S3 (continued): Analyses of variance (ANOVA) of inoculation effect on biogeochemical variables in four permafrost soils subjected to microbial community manipulation, and pairwise post-hoc comparisons where relevant (estimated marginal means). Values in bold indicate significant (Holm-adjusted *P* < 0.05) tests, n=4, except for Ammonium-Yedoma-PF-Day389 where n=3. †: Kruskal-Wallis X^2.

Estimated marginal means	Soil	Contrast	t-ratio	df	P	t-ratio	df	P
**Dissolved organic carbon**		*Control – Tussock AL*	3.79	9	0.013			
	Tussock-PF	*Control – Multifunctional*	3.02	9	0.029			
		Tussock AL – Multifunctional	-0.77	9	0.460		N.A.	
		Control – Palsa AL	4.85	9	0.003			
	Palsa-PF	*Control – Multifunctional*	2.58	9	0.060			
		Palsa AL – Multifunctional	-2.27	9	0.060			
		Control – Cryoturbated AL	-0.75	15	1.000	2.99	15	0.065
		Control – Tussock AL	-2.72	15	0.144	0.78	15	1.000
		Control – Palsa AL	0.97	15	1.000	-0.50	15	1.000
		Control – Multifunctional	-0.08	15	1.000	3.41	15	0.031
	Yedoma-PF	*Cryoturbated AL – Tussock AL*	-1.97	15	0.475	-2.20	15	0.218
		Cryoturbated AL – Palsa AL	1.71	15	0.644	-3.49	15	0.030
		Cryoturbated AL- Multifunctional	0.67	15	1.000	0.42	15	1.000
		Tussock AL – Palsa AL	3.68	15	0.022	-1.28	15	0.877
		Tussock AL – Multifunctional	2.64	15	0.149	2.63	15	0.115
		Palsa AL – Multifunctional	-1.04	15	1.000	3.91	15	0.014
**Total dissolved nitrogen**		*Control – Palsa AL*	2.65	9	0.053			
	Palsa-PF	*Control – Multifunctional*	5.08	9	0.002		N.A.	
		Palsa AL – Multifunctional	2.43	9	0.053			
**Ammonium**		*Control – Palsa AL*	2.88	9	0.037			
	Palsa-PF	*Control – Multifunctional*	4.70	9	0.003		N.A.	
		Palsa AL – Multifunctional	-1.82	9	0.102			
**Dissolved inorganic nitrogen**		*Control – Cryoturbated AL*				0.0†	2	0.500
	Cryoturbated-PF	*Control – Multifunctional*		N.A.		2.32†	2	0.010
		Cryoturbated AL – Multifunctional				-2.32†	2	0.010
		Control – Tussock AL				-1.03†	2	0.500
	Tussock-PF	*Control – Multifunctional*		N.A.		-2.51†	2	0.010
		Tussock AL – Multifunctional				-1.48†	2	0.010
		Control – Palsa AL	2.95	9	0.032			
	Palsa-PF	*Control – Multifunctional*	4.60	9	0.004		N.A.	
		Palsa AL – Multifunctional	1.65	9	0.133			
**pH**		*Control – Palsa AL*	-3.85	9	0.012			
	Palsa-PF	*Control – Multifunctional*	-2.90	9	0.035		N.A.	
		Palsa AL – Multifunctional	0.95	9	0.366			
**Organic nitrogen**		*Control – Tussock AL*	3.30	9	0.028			
	Tussock-PF	*Control – Multifunctional*	2.25	9	0.102		N.A.	
		Tussock AL – Multifunctional	-1.05	9	0.323			
		Control – Cryoturbated AL	-0.57	15	1.000			
		Control – Tussock AL	-2.46	15	0.212			
		Control – Palsa AL	1.54	15	0.725			
		Control – Multifunctional	-1.01	15	0.989			
	Yedoma-PF	*Cryoturbated AL – Tussock AL*	-1.89	15	0.472		N.A.	
		Cryoturbated AL – Palsa AL	2.11	15	0.365			
		Cryoturbated AL- Multifunctional	-0.44	15	1.000			
		Tussock AL – Palsa AL	4.00	15	0.012			
		Tussock AL – Multifunctional	1.45	15	0.725			
		Palsa AL – Multifunctional	-2.55	15	0.202			

Supplementary Table S4: Examples of published values of N_2O fluxes in permafrost systems.

Gross N_2O fluxes are measured in presence of acetylene to inhibit N_2O-reductase. Values from the experiments with settings most directly comparable to that found in this study (i.e. aerobic incubation without vegetation) are italicized. WFPS: Water-filled pore space; WC: water content; *: whole core in mesocosm; **: mg-N_2O-N.m^{-2}.d^{-1}; b.d.l.: below detection limit; -: not measured.

**Lead author**	**doi**	**Permafrost soil type**	**Location**	**Depth**	**Vegetation**	**Incubation conditions**			**WFPS**	**WC**	**Net N_2O flux**		**Gross N_2O flux**	
				**[cm]**		**Temp.**	**Length**	**Atmosphere**			**[µg N_2O-N . kg DW^{-1} . d^{-1}]**			
						°C	d		%	%	Min	Max	Min	Max
Wegner	10.3390/nitrogen3040040	Thaw slump, eroded Yedoma + Holocene, mineral soil	Siberia	0-30	Yes	4	40	anaerobic	52-67	21-28	0.00	0.00	-	-
Wegner	10.3390/nitrogen3040040	Thaw slump, eroded Yedoma + Holocene, mineral soil	Siberia	0-30	No	4	40	anaerobic	52-67	21-28	0.10	95.43		
Wegner	10.3390/nitrogen3040040	Thaw slump, Yedoma, Mineral soil	Siberia	0-30	Yes	4	40	anaerobic	45-74	17-29	27.85	216.63	-	-
Wegner	10.3390/nitrogen3040040	Thaw slump, Yedoma, Mineral soil	Siberia	0-30	No	4	40	anaerobic	45-74	17-29	0.00	25.16	-	-
Wegner	10.3390/nitrogen3040040	Thaw slump, Yedoma, Mineral soil	Siberia	20-40	No	4	40	anaerobic	n.d.	17	-	63.66	-	-
Wegner	10.3390/nitrogen3040040	Thaw slump, Yedoma, Mineral soil	Siberia	40-70	No	4	40	anaerobic	43	16	-	22.17	-	-
Rasmussen	10.1016/j.soilbio.2022.108840	Glacial Valley, mineral soil	Greenland	0-15		5	31	aerobic	-	-	*480.00*	*9600.00*		
Sanders	10.1007/s13280-021-01665-0	Floodplain, mineral soil	Siberia	25-30	No	5	126	aerobic	-	-	*0.00*	*0.04*	-	-
Marushchak	10.1038/s41467-021-27386-2	Thaw slump, eroded Yedoma + Holocene, mineral soil	Siberia	0-10	Yes	10	1-6	aerobic	52-94	-	0.12	0.14	-	-
Marushchak	10.1038/s41467-021-27386-2	Thaw slump, eroded Yedoma + Holocene, mineral soil	Siberia	0-10	No	10	1-6	aerobic	52-94	-	*-0.19*	*0.02*	-	-
Marushchak	10.1038/s41467-021-27386-2	Thaw slump, Holocene, mineral soil	Siberia	0-10	Yes	10	1-6	aerobic	13	-	-	0.12	-	-
Marushchak	10.1038/s41467-021-27386-2	Thaw slump, eroded Yedoma + Holocene, mineral soil	Siberia	0-10	Yes	10	1-6	anaerobic	52-94	-	95.28	606.48	393.60	2820.96
Marushchak	10.1038/s41467-021-27386-2	Thaw slump, eroded Yedoma + Holocene, mineral soil	Siberia	0-10	No	10	1-6	anaerobic	52-94	-	3.12	19.20	6.00	10.08
Marushchak	10.1038/s41467-021-27386-2	Thaw slump, Holocene, mineral soil	Siberia	0-10	Yes	10	1-6	anaerobic	13	-	-	606.48	-	2820.96
Voigt	10.1073/pnas.1702902114	Peat palsa	Finland	0-100*	Mesocosm	10	28	aerobic	-	-	0,14**	0,20**		
Voigt	10.1073/pnas.1702902114	Peat palsa	Finland	0-100*	Mesocosm	10	28	aerobic	-	-	0,56**	2,81**		
Palmer	10.1038/ismej.2011.172	peat plateau, cryoturbated peat soil	Russia	0-5	No	20	3.75	anaerobic	-	-	-	8964.29	-	13446.43
Palmer	10.1038/ismej.2011.172	peat plateau, non-turbated peat soil	Russia	0-5	Yes	20	3,75 - 6,67	anaerobic	-	-	-	0.00	-	58.83
Elberling	10.1038/NGEO803	Wetland, active layer, mineral soil	Greenland	0-60	Yes	7	150	aerobic	-	-	-	32,88 (144)	-	-
Elberling	10.1038/NGEO803	Heath tundra, active layer, mineral soil	Greenland	0-80	Yes	7	150	aerobic	-	-	-	b.d.l.	-	-
Elberling	10.1038/NGEO803	Wetland, permafrost, mineral soil	Greenland	60-300	Yes	7	150	aerobic	-	-	-	20.88	-	-
Elberling	10.1038/NGEO803	Heath tundra, permafrost, mineral soil	Greenland	80-300	Yes	7	150	aerobic	-	-	-	b.d.l.	-	-
Rodionow	10.1016/j.geoderma.2005.10.008	Forest – heath tundra ecotone	Siberia	0-50	No	5-15	0.25-0.4	aerobic	-	60-100	-	*0.72*	-	-
This study		Palsa-PF, day 7, control	Sweden	110-130	No	10	7	aerobic	-	28-32	0.89	1.56	-	-
This study		Palsa-PF, day 7, Palsa-AL inoculum	Sweden	110-130	No	10	7	aerobic	-	28-30	1.15	8.89	-	-
This study		Palsa-PF, day 7, Multifunctional inoculum	Sweden	110-130	No	10	7	aerobic	-	28-29	4.29	6.76	-	-
This study		Palsa-PF, day 17, Palsa-AL inoculum	Sweden	110-130	No	10	17	aerobic	-	28-30	0.44	0.46	-	-
This study		Palsa-PF, day 17, Multifunctional inoculum	Sweden	110-130	No	10	17	aerobic	-	28-29	9.46	13.86	-	-
This study		Tussock-PF, day 187, Multifunctional inoculum	Alaska	70-100	No	10	187	aerobic	-	36-38	1.09	7.46	-	-

Supplementary Table S5: Abiotics variables in pre-incubation permafrost and active layer inoculum. Numbers indicate mean ± SE, n=4. GWC: Gravimetric water content; OMC: organic matter content; DOC: Dissolved organic carbon; TN: total dissolved nitrogen. †: n=2; ‡: n=1.

		**GWC [%]**	**OMC [%]**	**DOC [mg.kgDW^{-1}]**	**TN [mg.kgDW^{-1}]**	**NH_4 [mg.kgDW^{-1}]**	**NO_3 [mg.kgDW^{-1}]**	**pH**	**Bulk density [g.cm^{-3}]**	**Dissolved DOC:TN ratio**
**Permafrost**	Cryoturbated-PF	19.5 ± 1.37	1.39 ± 0.03 †	129.18 ± 3.65	14.8 ± 0.57	2.18 ± 0.31	0.28 ± 0.06	8.01 ± 0.03	1.43	8.75 ± 0.26
	Tussock-PF	40.17 ± 0.57	15.16 ± 0.07	470.4 ± 12.56	60.4 ± 1.69	20.08 ± 0.8	0.45 ± 0.06	7.19 ± 0.05	n.d.	7.79 ± 0.02
	Palsa-PF	28.42 ± 0.42	3.91 ± 0.06	320.95 ± 12.05	35.43 ± 0.98	14.25 ± 0.33	0.58 ± 0.05	6.45 ± 0.06	0.14	9.06 ± 0.24
	Yedoma	39.59 ± 1.51	7.85 ± 0.05	1286.25 ± 24.65	151.35 ± 2.69	79.58 ± 2.31	0.28 ± 0.05	6.83 ± 0.02	0.78	8.5 ± 0.08
**Active layer / Inoculum**	Cryoturbated-AL †	23.46 ± 1.19	6.82 ± 0.62	25.45 ± 5.05	2.2 ± 1.1	< d.l. ‡	1.50 ‡	7.67 ‡	n.d.	13.89 ± 4.65 †
	Tussock-AL †	81.32 ± 2.00	86.81 ± 1.86	7688.35 ± 110.85	9.3 ± 6.4	< d.l. ‡	< d.l. ‡	5.46 ‡	n.d.	1554.85 ± 1058.08 †
	Palsa-AL †	79.1 ± 0.84	95.91 ± 0.11	452.85 ± 56.05	5.6 ± 6.5	< d.l. ‡	< d.l. ‡	4.91 ‡	n.d.	42.06 ‡
	Multifunctional †	25.81 ± 0.19	13.5 ± 0.20	15.15 ± 5.25	11.05 ± 3.25	1.10 ‡	5.10 ‡	6.12 ‡	n.d.	1.35 ± 0.08 †

Supplementary Table S6: Primers and conditions for the PCR reactions for 16S amplicon and for the qPCR assays for inhibition test, 16S rRNA gene, *amoA*, *nir* and *nos* functional genes. The qPCR reactions were carried out in two technical replicates with an initial denaturation at 95 °C for 5 min, and cycles of denaturation at 95 °C for 15 s, annealing for 30 s and elongation at 72 °C, fluorescence signal was read for 5 s at the end of each non-touchdown cycle, at 77 °C for amoA and nosZ-II and at 80 °C for nirS, nirK and nosZ-I, and primer dimers were melted at 78 °C for 5 s before reading signals for pmoA. Standard curves R^2 and efficiency were derived by serial dilutions of linearized plasmids containing cloned fragments of the specific genes in the range of 10^1-10^7 copies per reaction. Oligonucleotides purified by standard desalting (ThermoFisher Scientific).

		16S rRNA gene V4V5 amplicons		Indexing PCR	
Primers and sequences 5' - 3'		515F GTGYCAGCMGCCGCGGTAA<br>926R CCGYCAATTYMTTTRAGTTT		N501-N504, N506, N507, N510, N511<br>N701-N712	
Reference		Parada et al., 2016, Quince et al., 2011		Nextera XT Index Kit v2, Illumina	
Phusion Green PCR Master Mix		12.5 µl		15 µl	
Nuclease-free water		9.25 µl		7 µl	
DNA template volume		2 µl		5 µl	
DNA template dilution		5 ng . µl^{-1} *		-	
Primer concentration		10 µM		3 µM	
Primer volume		0.625 µl		3 µl	
Reaction volume		25 µl		30 µl	
Initial denaturation		98 °C 3 min		98 °C 3 min	
Cycles	Denaturation	×25†	98 °C 15 s	×8	98 °C 30 s
	Annealing		50 °C 30 s		55 °C 30 s
	Elongation		72 °C 40 s		72 °C 30 s
Final elongation		72 °C 10 min		72 °C 10 min	
PCR clean-up		SequalPrep Normalization Plate Kit<br>20 µl elution		SequalPrep Normalization Plate Kit<br>20 µl serial elution across columns	
PCR product quantification		NA		Qubit dsDNA HS<br>Agilent 2100 Bioanalyzer	

*: undiluted and- †: 30 cycles- for most Cryoturbated samples

qPCR assay target	Target gene	Primer	Primer sequence 5' - 3'	Primer reference	Final primer conc.	Cycles	Annealing temp.	Elongation time	R^2 / Efficiency %
Inhibition	TOPO vector	M13F<br>M13R	GTAAAACGACGGCCAG<br>CAGGAAACAGCTATGAC		0.1 µM	35	55 °C	45 s	NA
Prokaryotes	16S rRNA V4V5	515F<br>926R	GTGYCAGCMGCCGCGGTAA<br>CCGYCAATTYMTTTRAGTTT	Parada *et al.*, 2016<br>Quince *et al.*, 2011	0.2 µM	35	55 °C	30 s	0.995 / 94.4
Ammonia-oxidizing bacteria (AOB) *†	*amoA*	amoA-1F<br>amoA-2R	GGGGTTTCTACTGGTGGT<br>CCCCTCKGSAAAGCCTTCTTC	Rotthauwe *et al.*, 1997	0.5 µM	40	55 °C	40 s	0.995 / 108.0
Ammonia-oxidizing archaea (AOA)	*amoA*	CrenamoA23f<br>CrenamoA616R	ATGGTCTGGCTWAGACG<br>GCCATCCATCTGTATGTCCA	Tourna *et al.*, 2008	0.5 µM	40	55 °C	40 s	0.993 / 88.7
Nitrite reductase S	*nirS*	cd3aFm<br>R3cdm	AACGYSAAGGARACSGG<br>GASTTCGGRTGSGTCTTSAYGAA	Throbäck *et al.*, 2004	0.5 µM	6 c. → 35	65-1°C / c. → 60 °C	30 s	0.997 / 83.6
Nitrite reductase K	*nirK*	F1aCu<br>1040R	ATCATGGTSCTGCCGCG<br>GCCTCGATCAGRTTRTGGTT	Henry *et al.*, 2006	0.5 µM	6 c. → 35	63-1°C / c. → 58 °C	35 s	0.997 / 83.3
Nitrous oxide reducers clade I	*nosZ*	1840F<br>2090R	CGCRACGGCAASAAGGTSMSSGT<br>CAKRTGCAKSGCRTGGCAGAA	Henry *et al.*, 2006	0.5 µM	6 c. → 35	65-1°C / c. → 60 °C	30 s	0.997 / 78.6
Nitrous oxide reducers clader II	*nosZ*	nosZ-II_F<br>nosZ-II_R	CTIGGICCIYTKCAYAC<br>GCIGARCARAAITCBGTRC	Jones *et al.*, 2013	2 µM	40	54 °C	45 s	0.998 / 82.1
Inhibition (anoxic)	pGEM plasmid	Sp6<br>T7	ATTTAGGTGACACTATAG<br>TAATACGACTCACTATAGGG		0.1 µM	40	55 °C	30 s	0.999 / 90.4
Methanogens	*mcrA*	Mlas<br>mcrA rev	GGTGGTGTMGGDTTCACMCARTA<br>CGTTCATBGCGTAGTTVGGRTAGT	Steinberg & Regan 2008	0.4 µM	40	57 °C	20 s	0.999 / 90.4
Methane oxidizers *	*pmoA*	A189gc<br>mb661	GGNGACTGGGACTTCTGG<br>CCGGMGCAACGTCYTTACC	Holmes *et al.*, 1995<br>Costello & Lidstrom 1999	0.2 µM	40	60 °C	20 s	0.980 / 90.6

*specific-size bands could not be obtained for most samples; † R^2/efficiency based on one replicate.

Supplementary Table S7: Quantitative data obtained from qPCR analyses. Empty cells indicate that no quantitative data could be obtained, italicized values were below the detection limit of 10 copies per reaction. DNA: DNA concentration in extracts; 16S: 16S rRNA gene V4V5 region; AOA: ammonia-oxidizing archaea *amoA* gene; *nirS*: nitrite reductase S gene; *nirK*: nitrite reductase K gene; *nosZ*-I: nitrous oxide reductase clade I gene; *nosZ*-II: nitrous oxide reductase clade II gene.

					Gene copy number per reaction					
**Soil**	**Date**	**Treatment**	**Replicate**	**DNA [ng.ul^{-1}]**	**16S**	**AOA**	***nirS***	***nirK***	***nosZ*-I**	***nosZ*-II**
Cryoturbated-PF	Pre-incubation permafrost		1	0.059	23					
			2	0.054	29					
Tussock-PF	Pre-incubation permafrost		1	1.61	65995				27	
			2	2.16	175951		*2*	84	23	14
			3	1.31	68453					12
Palsa-PF	Pre-incubation permafrost		2	0.108	501					
Yedoma-PF	Pre-incubation permafrost		1	0.263	985					
			2	0.722	13790					
			3	0.666	1921					
	Cryoturbated-AL inoculum		1	46.9	201643	789	207	1889	235	1057
			2	56	155588	560	205	1556		736
			3	34.7	135314	309	224	1374	19	633
			4	15.8	247793	1557	304	2273	422	1638
	Tussock-AL inoculum		1	47.9	116127				52	
			2	33.2	105807			337	42	
			3	40.1	93251				17	
			4	39.3	95130				27	
	Palsa-AL inoculum		1	> 60	71953			160		
			2	56	71714		*6*	158		
			3	57	141290			276		
			4	55	99661		13		*7*	
	Multifunctional inoculum		1	> 60	139888	2265	41	2178		721
			3	> 60	95130	1729	32	1438		492
			4	> 60	94499	1393	27	1149		425
Cryoturbated-PF	Day 1	Control	1	0.097	40					
			2	0.061	270					
			3	0.051	42					
		Cryoturbated-AL	2	0.077	46					
			4	4.66	210547	26	30	1855	244	657
	Day 389	Control	1	0.062	27					
			2	0.054	49			*2*		
			3	0.108	45					
			4	0.128	22					
		Multifunctional	1	0.116	53					
Tussock-PF	Day 1	Control	1	3.05	452263			156	20	
			2	2.41	472236			*8*	73	
			3	4.11	208458			158	19	
			4	4.83	199642			84	25	
		Tussock-AL	1	4.3	293573			106	17	58
			3	3.14	253627		11	116	34	
			4	4.46	261329			91	18	
		Multifunctional	1	3.49	323282	436	12	211	62	40
			2	6.26	295531	321		154	25	
			3	3.76	357184	368	*10*	218	66	27
			4	3.02	287775	152		127	28	
	Day 389	Control	1	22	184947		18	268	788	240
			2	20.4	288734		29	539	1142	206
			3	19.6	205022		42	539	1297	223
			4	16.6	208458		24	727	795	227
		Tussock-AL	2	28.1	278367		56	453	1206	263
			3	21.6	155588		13	231	697	154
			4	17.9	211952		50	230	804	284
		Multifunctional	2	26.7	178306	78	67	1720	534	1251
			3	15	302488	31	244	2785	681	626
			4	17.9	209152	56	139	2192	226	1360

Supplementary Table S7 (continued): Quantitative data obtained from qPCR analyses.

Soil	Date	Treatment	Replicate	DNA [ng.ul-1]	Gene copy number per reaction 16S	AOA	*nirS*	*nirK*	*nosZ*-I	*nosZ*-II
Palsa-PF	Day 1	Control	1	0.125	4791					
			2	0.151	577					
			3	0.117	2384					*2*
			4	0.137	311					
		Palsa-AL	1	0.169	2143					*3*
			2	0.198	3090					
			3	0.182	3747					
			4	0.173	556					
		Multifunctional	1	0.463	29818	227		76	21	33
			2	0.265	10570	52	*1*	32	*10*	*8*
			3	0.163	12113	96	*2*	51	*9*	14
			4	0.142	5677	41		13		*5*
	Day 389	Control	1	6.28	150002		11	550	280	
			2	1.63	211248			2266	401	
			3	4.31	122065			982	190	670
			4	6.02	164087		14	988	240	1277
		Palsa-AL	1	7.46	107938			616	275	949
			2	4.87	113456		14	682	194	478
			3	6.49	151505			1129	540	2076
			4	6.97	143658			633	354	666
		Multifunctional	1	3.61	134418	53		2172	373	231
			2	2.65	149504	100		1501	364	246
			3	3.98	171334	97		2126	248	241
			4	6.48	175367	144		1699	232	403
Yedoma-PF	Day 1	Control	1	1.07	222049			*4*		
			2	1.27	130023					
			3	1.68	407979				*10*	
			4	1.5	440395					
		Cryoturbated-AL	1	0.764	63204	22		18		
			2	1.82	388135	36		32	24	*9*
			3	0.853	114594			*6*		
			4	0.704	127455	39	*1*	32	26	*9*
		Tussock-AL	1	1.33	319012					
			2	1.76	762159			12	11	
			3	1.3	152010					
			4	1.91	438933					
		Palsa-AL	1	2.45	334209			20	*7*	
			2	1.45	220577					11
			3	1.23	228033		*1*	*5*		
			4	1.08	366810					
		Multifunctional	1	1.88	333100	233		26	14	
			2	1.17	151002	567		52	16	22
			3	1.22	106867	379		47	14	19
			4	13.1	105807	106		11	*4*	*8*
	Day 389	Control	1	29.9	380471			54	296	
			2	12.2	364379			166	28	391
			3	38.1	380471			796	344	
			4	38.8	388135		16	85		
		Cryoturbated-AL	1	21.1	196351		77	1941	126	321
			2	19.7	395954		132	2645	280	740
			3	21.6	186181		49	1628	92	734
			4	22.5	239691		149	2959	272	1214
		Tussock-AL	1	22.1	455280			239	*7*	536
			2	16.8	443332		16	164	14	196
			3	16.7	472236		*5*	183	70	118
			4	6.92	416198			3013	262	164
		Palsa-AL	1	5.04	493090		*10*	544	230	56
			2	16.7	458317			56	423	67
			3	28.4	456796			2400	818	
			4	14.8	478557			68	408	655
		Multifunctional	1	4.54	200973		37	2393	376	540
			2	8.36	186181		263	1901	217	710
			3	8.85	271964	86	316	3782	420	1825
			4	4.94	295531	24	25	2343	336	949
