# Supplementary material for: Active layer microbial inocula restore missing functions across thawed permafrost soils
Source: Nat Commun. 2026 Sep 25;17:10230. doi: 10.1038/s41467-026-78060-4 (PMC13614969; doi:10.1038/s41467-026-78060-4)
Supplement: Supplementary file 2 [file 41467_2026_78060_MOESM2_ESM.pdf]

nature portfolio

Corresponding author(s): Sylvain Monteux

Last updated by author(s): Jul 22, 2026

Reporting Summary

Nature Portfolio wishes to improve the reproducibility of the work that we publish. This form provides structure for consistency and transparency in reporting. For further information on Nature Portfolio policies, see our Editorial Policies and the Editorial Policy Checklist.

Statistics

For all statistical analyses, confirm that the following items are present in the figure legend, table legend, main text, or Methods section.

n/a	Confirmed	
☐	☒	The exact sample size (*n*) for each experimental group/condition, given as a discrete number and unit of measurement
☐	☒	A statement on whether measurements were taken from distinct samples or whether the same sample was measured repeatedly
☐	☒	The statistical test(s) used AND whether they are one- or two-sided<br>*Only common tests should be described solely by name; describe more complex techniques in the Methods section.*
☐	☒	A description of all covariates tested
☐	☒	A description of any assumptions or corrections, such as tests of normality and adjustment for multiple comparisons
☐	☒	A full description of the statistical parameters including central tendency (e.g. means) or other basic estimates (e.g. regression coefficient) AND variation (e.g. standard deviation) or associated estimates of uncertainty (e.g. confidence intervals)
☐	☒	For null hypothesis testing, the test statistic (e.g. *F*, *t*, *r*) with confidence intervals, effect sizes, degrees of freedom and *P* value noted<br>*Give P values as exact values whenever suitable.*
☒	☐	For Bayesian analysis, information on the choice of priors and Markov chain Monte Carlo settings
☒	☐	For hierarchical and complex designs, identification of the appropriate level for tests and full reporting of outcomes
☒	☐	Estimates of effect sizes (e.g. Cohen's *d*, Pearson's *r*), indicating how they were calculated

Our web collection on statistics for biologists contains articles on many of the points above.

Software and code

Policy information about availability of computer code

Data collection	No software was used to collect data - enaGroupGet 1.7.0 from the enaBrowserTools suite is suggested as a mean to access our raw sequencing data hosted at ENA.
Data analysis	All code used to process the raw sequencing data and generate figures and statistics mentioned in text and all display elements is found together with the data at https://doi.org/10.57669/monteux-2026-permafrost-inoculation-1.1.0<br>All software versions and dependencies are described within that repository, in the main README.md file and in the renv.lock file for all packages in the R environment. enaGroupGet 1.7.0 and cutadapt v5.0 were used upstream, all other analayses were carried out with R v4.5.2 with packages VSURF_1.2.1, rfPermute_2.5.5, ggpmisc_0.6.3, ggpp_0.6.0, extrafont_0.19, dunn.test_1.3.6, sciplot_1.2-0, reshape2_1.4.5, ez_4.4-0, stringi_1.8.7, nlme_3.1-168, rstatix_0.7.2, patchwork_1.3.0, mvabund_4.2.1, vegan_2.7-2, permute_0.9-10, emmeans_1.11.1 lubridate_1.9.4, forcats_1.0.0, purrr_1.2.1, tidyr_1.3.2, ggplot2_3.5.2, tidyverse_2.0.0, Biostrings_2.78.0, Seqinfo_1.0.0, XVector_0.50.0, IRanges_2.44.0, S4Vectors_0.48.0, BiocGenerics_0.56.0, generics_0.1.4, decontam_1.28.0, tibble_3.3.1, phyloseq_1.54.0, dplyr_1.2.0, readr_2.1.5, stringr_1.6.0, dada2_1.36.0 and Rcpp_1.1.1

For manuscripts utilizing custom algorithms or software that are central to the research but not yet described in published literature, software must be made available to editors and reviewers. We strongly encourage code deposition in a community repository (e.g. GitHub). See the Nature Portfolio guidelines for submitting code & software for further information.

Data

Policy information about availability of data

All manuscripts must include a data availability statement. This statement should provide the following information, where applicable:

- Accession codes, unique identifiers, or web links for publicly available datasets
- A description of any restrictions on data availability
- For clinical datasets or third party data, please ensure that the statement adheres to our policy

All our raw sequencing data is available at ENA under accession number PRJEB97802
All other data are found together with the code used to process them at https://doi.org/10.57669/monteux-2026-permafrost-inoculation-1.1.0
Source Data is provided with the manuscript

Research involving human participants, their data, or biological material

Policy information about studies with human participants or human data. See also policy information about sex, gender (identity/presentation), and sexual orientation and race, ethnicity and racism.

Reporting on sex and gender	N/A
Reporting on race, ethnicity, or other socially relevant groupings	N/A
Population characteristics	N/A
Recruitment	N/A
Ethics oversight	N/A

Note that full information on the approval of the study protocol must also be provided in the manuscript.

Field-specific reporting

Please select the one below that is the best fit for your research. If you are not sure, read the appropriate sections before making your selection.

☐ Life sciences ☐ Behavioural & social sciences ☒ Ecological, evolutionary & environmental sciences

For a reference copy of the document with all sections, see nature.com/documents/nr-reporting-summary-flat.pdf

Life sciences study design

All studies must disclose on these points even when the disclosure is negative.

Sample size	*Describe how sample size was determined, detailing any statistical methods used to predetermine sample size OR if no sample-size calculation was performed, describe how sample sizes were chosen and provide a rationale for why these sample sizes are sufficient.*
Data exclusions	*Describe any data exclusions. If no data were excluded from the analyses, state so OR if data were excluded, describe the exclusions and the rationale behind them, indicating whether exclusion criteria were pre-established.*
Replication	*Describe the measures taken to verify the reproducibility of the experimental findings. If all attempts at replication were successful, confirm this OR if there are any findings that were not replicated or cannot be reproduced, note this and describe why.*
Randomization	*Describe how samples/organisms/participants were allocated into experimental groups. If allocation was not random, describe how covariates were controlled OR if this is not relevant to your study, explain why.*
Blinding	*Describe whether the investigators were blinded to group allocation during data collection and/or analysis. If blinding was not possible, describe why OR explain why blinding was not relevant to your study.*

Behavioural & social sciences study design

All studies must disclose on these points even when the disclosure is negative.

Study description	*Briefly describe the study type including whether data are quantitative, qualitative, or mixed-methods (e.g. qualitative cross-sectional, quantitative experimental, mixed-methods case study).*
Research sample	*State the research sample (e.g. Harvard university undergraduates, villagers in rural India) and provide relevant demographic information (e.g. age, sex) and indicate whether the sample is representative. Provide a rationale for the study sample chosen. For*

studies involving existing datasets, please describe the dataset and source.

Sampling strategy
Describe the sampling procedure (e.g. random, snowball, stratified, convenience). Describe the statistical methods that were used to predetermine sample size OR if no sample-size calculation was performed, describe how sample sizes were chosen and provide a rationale for why these sample sizes are sufficient. For qualitative data, please indicate whether data saturation was considered, and what criteria were used to decide that no further sampling was needed.

Data collection
Provide details about the data collection procedure, including the instruments or devices used to record the data (e.g. pen and paper, computer, eye tracker, video or audio equipment) whether anyone was present besides the participant(s) and the researcher, and whether the researcher was blind to experimental condition and/or the study hypothesis during data collection.

Timing
Indicate the start and stop dates of data collection. If there is a gap between collection periods, state the dates for each sample cohort.

Data exclusions
If no data were excluded from the analyses, state so OR if data were excluded, provide the exact number of exclusions and the rationale behind them, indicating whether exclusion criteria were pre-established.

Non-participation
State how many participants dropped out/declined participation and the reason(s) given OR provide response rate OR state that no participants dropped out/declined participation.

Randomization
If participants were not allocated into experimental groups, state so OR describe how participants were allocated to groups, and if allocation was not random, describe how covariates were controlled.

Ecological, evolutionary & environmental sciences study design

All studies must disclose on these points even when the disclosure is negative.

Study description
The study consists in two main incubation experiments:
- a 389 days oxic incubation of three permafrost soils subjected to inoculation with soil suspensions from their own active layer soil, a temperate grassland (positive control) or sterile water (negative control). A fourth permafrost soil is subjected to inoculation with positive and negative control as well as all three active layer soil inoculums used for the other permafrost soils. Each soil:treatment combination consists in 4 replicate flasks prepared from the same homogenized permafrost core. This results in (4 replicates * 3 treatments * 3 soils) + (4 replicates * 5 treatments * 1 soil) = 56 experimental units. CO_2 fluxes were measured throughout the 389 days incubation, N_2O & CH_4 were measured at days 7, 17, 40, 187, and soil chemistry, bacterial community composition and functional genes were measured after 389 days of incubation. The entire design was duplicated to allow for assessing putative artefacts one day after inoculation. In addition, 4 subsamples of the initial permafrost after homogenization but prior to pre-incubation, and 3 subsamples of the initial active layer soils, were analyzed for initial soil chemistry, bacterial community composition and functional genes.
- an anoxic incubation of 175 days of one permafrost soil under a N_2 atmosphere, inoculated with a soil suspension prepared with either fresh or gamma-irradiated active layer (negative control). Each of the 2 treatments were replicated 8 times, resulting in 16 experimental units.

Research sample
Cryoturbated permafrost: sampled from the rim of a non-sorted circle (frost boil) in the Alaskan North Slope (Franklin Bluffs, 69.6685N, 148.7329W, 2012, 70-100 cm depth)
Tussock permafrost: sedge peat histel sampled from an Eriophorum vaginatum-dominated tussock tundra (moist acidic tundra) of the Alaskan North Slope (Ice Cut, 69.0467N, 148.8343W, 2015, 70-100 cm depth)
Palsa permafrost: histel sampled from a Sphagnum-dominated ombrotrophic peat bog underlain by silty permafrost (palsa mire) in sub-arctic Sweden (Storflaket, 68.3462N, 18.9714E, 2015, 110-150 cm depth)
Yedoma permafrost: Yedoma sediment originated from the Cold Region Research Engineering Laboratory (CRREL) Permafrost Tunnel (Fox, Alaska, USA, 64.9513N, 147.6206W, 2012, 15 m depth). The soil was sampled from the upper silt unit originating from an upper Pleistocene silty deposit, previously described in ref. 10 [https://doi.org/10.1038/s41561-020-00662-4].
Active layer soils: collected at 5-15cm depth within the same cores used for permafrost samples.
Temperate grassland soil used as positive control: surface layer (0-15 cm depth) of a drained Cambisol developed on granitic bedrock at the LTER research site of the French National Research Institute for Agriculture, Food and the Environment (INRAE) in central France (45°43' N, 03°01' E). The site is an experimental grassland, specifically an abandonment-treatment which had not undergone any cutting or fertilization for 15 years prior to soil sampling in the winter 2018-2019.

Sampling strategy
Single cores were selected from the permafrost cores collections, cores were selected to ensure sufficient material for the 112 flasks. No effect-size calculation or power analyses were carried out prior to the experiment, but we increased replication compared to our 2020 study ref. 10 [https://doi.org/10.1038/s41561-020-00662-4] from 3 to 4 experimental units.

Data collection
Data for CO_2 fluxes were recorded on paper and as log files by Sylvain Monteux and Josefine Walz upon measurement with an EGM5 infra-red gas analyzer. N_2O/CH_4 measurements for the oxic incubation were carried out by Sébastien Fontaine on a gas chromatograph (GC, Clarus 480) equipped with a photoionization detector (PID, Vici 07709-S). CO_2, N_2O and CH_4 measurements for the anoxic incubation were carried by Sylvain Monteux and Magnus Widesheim on a Agilent 7890 GC coupled with a flame ionization detector (FID, CO_2 and CH_4) and an electron capture detector (ECD, N_2O).
Data on soil moisture, soil organic matter content, total dissolved organic carbon, total dissolved nitrogen were collected by Sylvain Monteux, using respectively a drying oven, furnace, and high temperature catalytic oxidation using a Shimadzu TOC-V CPH analyser with a TN unit (Shimadzu Corporation, Japan) for TDC and TN.
Ammonium and nitrate-nitrite were measured on a a AA3 system (SEAL Analytical Ltd, United Kingdom) by Mina Spångberg at the SLU Uppsala Department of Soil and Environment accredited laboratory.
Amplicon data was collected by Sylvain Monteux as described in text.
qPCR data for amoA, nirS, nirK, nosZI, nosZII and 16S rRNA were collected by Jaanis Juhanson on CFX Connect or CFX-96 Real-Time

	System thermocyclers (Bio-Rad laboratories, USA).<br>qPCR data for mcrA and pmoA were collected by Sylvain Monteux on CFX Connect thermocyclers (Bio-Rad Laboratories, USA).
Timing and spatial scale	The oxic incubation lasted 389 days at 10 degrees, CO2 measurements were carried at varying intervals described in Supplementary Figure S1 to ensure that CO2 concentration in the headspace never exceeded 3%. CH4 and N2O were measured from samples taken at days 7, 17, 40 and 187 - concomitantly with the CO2 measurements taking place on these dates. All destructive analyses were performed at day 1 (to assess putative changes immediately induced by inoculation) and at the end of the incubation at day 389.<br>The anoxic incubation lasted 175 days at 10 degrees, CO2, CH4 and N2O were measured after 2, 4, 6, 12 and 25 weeks.
Data exclusions	Data were excluded for DNA data when samples did not pass quality filtering (as described in text), for ASVs that were identified as potential contaminants (described in text and logged in code/data repository), and for all other measurements when they were below detection limit. Deviations from the original design are consistently indicated throughout the manuscript and figures where numbers indicate the number of samples above detection limit whenever this is not 4.
Reproducibility	Part of the experiment (Yedoma sediment * negative/positive controls) reproduces our design in ref. 10 [https://doi.org/10.1038/s41561-020-00662-4] with a different permafrost core from the same sampling campaign/location, but also a different inoculum method (here liquid, in ref. 10 solid). An additional incubation presented in Supplementary Materials compares these two types of inoculum and effectively reproduces the results from both ref. 10 and this study for the (Yedoma sediment * negative/positive controls) part of the design. We deemed this sufficient to indicate the methodology is robust and reproducible, and did not attempt to reproduce the entire experimental design.<br>All code used through the analyses is provided at https://doi.org/10.57669/monteux-2026-permafrost-inoculation-1.1.0 to allow reproducing all in silico analyses from raw sequencing data to the generation of all figures, tables and individual statistical tests.
Randomization	Within a soil type, inoculation treatments were allocated randomly across flasks.
Blinding	No particular blinding was carried out in this study. Molecular analyses were carried out referring to samples with a unique number which did not intuitively match the experimental design. Data collected externally (e.g. N2O, DIN, qPCR) was collected without specific knowledge of the sample codes - experimental treatment correspondence, which may be considered blinding, but without any explicit intent to do so.

Did the study involve field work? ☒ Yes ☐ No

Field work, collection and transport

Field conditions	Permafrost and active layer cores were sampled in the field.
Location	Cryoturbated: Alaska USA, North Slope (Franklin Bluffs, 69.6685N, 148.7329W, 2012, 70-100 cm depth)<br>Tussock: Alaska USA, North Slope (Ice Cut, 69.0467N, 148.8343W, 2015, 70-100 cm depth)<br>Palsa: Norrbottens län, Sweden (Storflaket, 68.3462N, 18.9714E, 2015, 110-150 cm depth)<br>Yedoma: Alaska USA, Fox, (Cold Region Research Engineering Laboratory (CRREL) Permafrost Tunnel 64.9513N, 147.6206W, 2012, 15 m depth).<br>Positive control: Puy de Dôme, France (Theix, LTER research site of the French National Research Institute for Agriculture, Food and the Environment (INRAE) 45°43' N, 03°01' E, 2018-2019, 0-15cm depth).
Access & import/export	Samples were obtained in agreement with the relevant authorities as per guidelines from the field stations (Toolik Lake Field Station, Alaska USA and Abisko Scientific Research Station, Norrobottens län Sweden). Sampling in the CRREL permafrost tunnel was carried out under the supervision and with permission from CRREL staff, notably Thomas Douglas. No import/export or sampling permits were required.
Disturbance	The diameter of the cores was small and drilling caused minimal disturbance to the ecosystems. After sampling, we replaced the top layer of soil including vegetation in the drill holes to minimize impact.

Reporting for specific materials, systems and methods

We require information from authors about some types of materials, experimental systems and methods used in many studies. Here, indicate whether each material, system or method listed is relevant to your study. If you are not sure if a list item applies to your research, read the appropriate section before selecting a response.

Materials & experimental systems

n/a	Involved in the study
☒	☐ Antibodies
☒	☐ Eukaryotic cell lines
☒	☐ Palaeontology and archaeology
☒	☐ Animals and other organisms
☒	☐ Clinical data
☒	☐ Dual use research of concern
☒	☐ Plants

Methods

n/a	Involved in the study
☒	☐ ChIP-seq
☒	☐ Flow cytometry
☒	☐ MRI-based neuroimaging

Antibodies

Antibodies used: *Describe all antibodies used in the study; as applicable, provide supplier name, catalog number, clone name, and lot number.*

Validation: *Describe the validation of each primary antibody for the species and application, noting any validation statements on the manufacturer's website, relevant citations, antibody profiles in online databases, or data provided in the manuscript.*

Eukaryotic cell lines

Policy information about cell lines and Sex and Gender in Research

Cell line source(s): *State the source of each cell line used and the sex of all primary cell lines and cells derived from human participants or vertebrate models.*

Authentication: *Describe the authentication procedures for each cell line used OR declare that none of the cell lines used were authenticated.*

Mycoplasma contamination: *Confirm that all cell lines tested negative for mycoplasma contamination OR describe the results of the testing for mycoplasma contamination OR declare that the cell lines were not tested for mycoplasma contamination.*

Commonly misidentified lines (See ICLAC register): *Name any commonly misidentified cell lines used in the study and provide a rationale for their use.*

Palaeontology and Archaeology

Specimen provenance: *Provide provenance information for specimens and describe permits that were obtained for the work (including the name of the issuing authority, the date of issue, and any identifying information). Permits should encompass collection and, where applicable, export.*

Specimen deposition: *Indicate where the specimens have been deposited to permit free access by other researchers.*

Dating methods: *If new dates are provided, describe how they were obtained (e.g. collection, storage, sample pretreatment and measurement), where they were obtained (i.e. lab name), the calibration program and the protocol for quality assurance OR state that no new dates are provided.*

☐ Tick this box to confirm that the raw and calibrated dates are available in the paper or in Supplementary Information.

Ethics oversight: *Identify the organization(s) that approved or provided guidance on the study protocol, OR state that no ethical approval or guidance was required and explain why not.*

Note that full information on the approval of the study protocol must also be provided in the manuscript.

Animals and other research organisms

Policy information about studies involving animals; ARRIVE guidelines recommended for reporting animal research, and Sex and Gender in Research

Laboratory animals: *For laboratory animals, report species, strain and age OR state that the study did not involve laboratory animals.*

Wild animals: *Provide details on animals observed in or captured in the field; report species and age where possible. Describe how animals were caught and transported and what happened to captive animals after the study (if killed, explain why and describe method; if released, say where and when) OR state that the study did not involve wild animals.*

Reporting on sex: *Indicate if findings apply to only one sex; describe whether sex was considered in study design, methods used for assigning sex. Provide data disaggregated for sex where this information has been collected in the source data as appropriate; provide overall numbers in this Reporting Summary. Please state if this information has not been collected. Report sex-based analyses where performed, justify reasons for lack of sex-based analysis.*

Field-collected samples	*For laboratory work with field-collected samples, describe all relevant parameters such as housing, maintenance, temperature, photoperiod and end-of-experiment protocol OR state that the study did not involve samples collected from the field.*
Ethics oversight	*Identify the organization(s) that approved or provided guidance on the study protocol, OR state that no ethical approval or guidance was required and explain why not.*

Note that full information on the approval of the study protocol must also be provided in the manuscript.

Clinical data

Policy information about clinical studies

All manuscripts should comply with the ICMJE guidelines for publication of clinical research and a completed CONSORT checklist must be included with all submissions.

Clinical trial registration	*Provide the trial registration number from ClinicalTrials.gov or an equivalent agency.*
Study protocol	*Note where the full trial protocol can be accessed OR if not available, explain why.*
Data collection	*Describe the settings and locales of data collection, noting the time periods of recruitment and data collection.*
Outcomes	*Describe how you pre-defined primary and secondary outcome measures and how you assessed these measures.*

Dual use research of concern

Policy information about dual use research of concern

Hazards

Could the accidental, deliberate or reckless misuse of agents or technologies generated in the work, or the application of information presented in the manuscript, pose a threat to:

No	Yes	
☒	☐	Public health
☒	☐	National security
☒	☐	Crops and/or livestock
☒	☐	Ecosystems
☒	☐	Any other significant area

Experiments of concern

Does the work involve any of these experiments of concern:

No	Yes	
☒	☐	Demonstrate how to render a vaccine ineffective
☒	☐	Confer resistance to therapeutically useful antibiotics or antiviral agents
☒	☐	Enhance the virulence of a pathogen or render a nonpathogen virulent
☒	☐	Increase transmissibility of a pathogen
☒	☐	Alter the host range of a pathogen
☒	☐	Enable evasion of diagnostic/detection modalities
☒	☐	Enable the weaponization of a biological agent or toxin
☒	☐	Any other potentially harmful combination of experiments and agents

Plants

Seed stocks: N/A

Novel plant genotypes: N/A

Authentication: N/A

ChIP-seq

Data deposition

☐ Confirm that both raw and final processed data have been deposited in a public database such as GEO.

☐ Confirm that you have deposited or provided access to graph files (e.g. BED files) for the called peaks.

Data access links
May remain private before publication.: *For "Initial submission" or "Revised version" documents, provide reviewer access links. For your "Final submission" document, provide a link to the deposited data.*

Files in database submission: *Provide a list of all files available in the database submission.*

Genome browser session
(e.g. UCSC): *Provide a link to an anonymized genome browser session for "Initial submission" and "Revised version" documents only, to enable peer review. Write "no longer applicable" for "Final submission" documents.*

Methodology

Replicates: *Describe the experimental replicates, specifying number, type and replicate agreement.*

Sequencing depth: *Describe the sequencing depth for each experiment, providing the total number of reads, uniquely mapped reads, length of reads and whether they were paired- or single-end.*

Antibodies: *Describe the antibodies used for the ChIP-seq experiments; as applicable, provide supplier name, catalog number, clone name, and lot number.*

Peak calling parameters: *Specify the command line program and parameters used for read mapping and peak calling, including the ChIP, control and index files used.*

Data quality: *Describe the methods used to ensure data quality in full detail, including how many peaks are at FDR 5% and above 5-fold enrichment.*

Software: *Describe the software used to collect and analyze the ChIP-seq data. For custom code that has been deposited into a community repository, provide accession details.*

Flow Cytometry

Plots

Confirm that:

☐ The axis labels state the marker and fluorochrome used (e.g. CD4-FITC).

☐ The axis scales are clearly visible. Include numbers along axes only for bottom left plot of group (a 'group' is an analysis of identical markers).

☐ All plots are contour plots with outliers or pseudocolor plots.

☐ A numerical value for number of cells or percentage (with statistics) is provided.

Methodology

Sample preparation: *Describe the sample preparation, detailing the biological source of the cells and any tissue processing steps used.*

Instrument: *Identify the instrument used for data collection, specifying make and model number.*

Software: *Describe the software used to collect and analyze the flow cytometry data. For custom code that has been deposited into a community repository, provide accession details.*

Cell population abundance | *Describe the abundance of the relevant cell populations within post-sort fractions, providing details on the purity of the samples and how it was determined.*

Gating strategy | *Describe the gating strategy used for all relevant experiments, specifying the preliminary FSC/SSC gates of the starting cell population, indicating where boundaries between "positive" and "negative" staining cell populations are defined.*

☐ Tick this box to confirm that a figure exemplifying the gating strategy is provided in the Supplementary Information.

Magnetic resonance imaging

Experimental design

Design type | *Indicate task or resting state; event-related or block design.*

Design specifications | *Specify the number of blocks, trials or experimental units per session and/or subject, and specify the length of each trial or block (if trials are blocked) and interval between trials.*

Behavioral performance measures | *State number and/or type of variables recorded (e.g. correct button press, response time) and what statistics were used to establish that the subjects were performing the task as expected (e.g. mean, range, and/or standard deviation across subjects).*

Acquisition

Imaging type(s) | *Specify: functional, structural, diffusion, perfusion.*

Field strength | *Specify in Tesla*

Sequence & imaging parameters | *Specify the pulse sequence type (gradient echo, spin echo, etc.), imaging type (EPI, spiral, etc.), field of view, matrix size, slice thickness, orientation and TE/TR/flip angle.*

Area of acquisition | *State whether a whole brain scan was used OR define the area of acquisition, describing how the region was determined.*

Diffusion MRI ☐ Used ☐ Not used

Preprocessing

Preprocessing software | *Provide detail on software version and revision number and on specific parameters (model/functions, brain extraction, segmentation, smoothing kernel size, etc.).*

Normalization | *If data were normalized/standardized, describe the approach(es): specify linear or non-linear and define image types used for transformation OR indicate that data were not normalized and explain rationale for lack of normalization.*

Normalization template | *Describe the template used for normalization/transformation, specifying subject space or group standardized space (e.g. original Talairach, MNI305, ICBM152) OR indicate that the data were not normalized.*

Noise and artifact removal | *Describe your procedure(s) for artifact and structured noise removal, specifying motion parameters, tissue signals and physiological signals (heart rate, respiration).*

Volume censoring | *Define your software and/or method and criteria for volume censoring, and state the extent of such censoring.*

Statistical modeling & inference

Model type and settings | *Specify type (mass univariate, multivariate, RSA, predictive, etc.) and describe essential details of the model at the first and second levels (e.g. fixed, random or mixed effects; drift or auto-correlation).*

Effect(s) tested | *Define precise effect in terms of the task or stimulus conditions instead of psychological concepts and indicate whether ANOVA or factorial designs were used.*

Specify type of analysis: ☐ Whole brain ☐ ROI-based ☐ Both

Statistic type for inference | *Specify voxel-wise or cluster-wise and report all relevant parameters for cluster-wise methods.*

(See Eklund et al. 2016)

Correction | *Describe the type of correction and how it is obtained for multiple comparisons (e.g. FWE, FDR, permutation or Monte Carlo).*

Models & analysis

n/a	Involved in the study
☐	☐ Functional and/or effective connectivity
☐	☐ Graph analysis
☐	☐ Multivariate modeling or predictive analysis

Functional and/or effective connectivity

Report the measures of dependence used and the model details (e.g. Pearson correlation, partial correlation, mutual information).

Graph analysis

Report the dependent variable and connectivity measure, specifying weighted graph or binarized graph, subject- or group-level, and the global and/or node summaries used (e.g. clustering coefficient, efficiency, etc.).

Multivariate modeling and predictive analysis

Specify independent variables, features extraction and dimension reduction, model, training and evaluation metrics.
