# Supplementary material for: Active layer microbial inocula restore missing functions across thawed permafrost soils
Source: Nat Commun. 2026 Sep 25;17:10230. doi: 10.1038/s41467-026-78060-4 (PMC13614969; doi:10.1038/s41467-026-78060-4)
Supplement: Supplementary file 3 [file 41467_2026_78060_MOESM3_ESM.pdf]

nature portfolio

Peer Review File

Active layer microbial inocula restore missing functions across thawed permafrost soils

Corresponding Author: Dr Sylvain Monteux

This file contains all reviewer reports in order by version, followed by all author rebuttals in order by version.

Version 0:

Reviewer comments:

Reviewer #1

(Remarks to the Author)
Review report on manuscript by Monteux et al.

GENERAL COMMENT
Here, the authors have presented a thrilling study, where they investigated occurrence of microbial limitations for CO_2, CH_4 and N_2O production in permafrost. This was achieved by inoculating permafrost with inocula from active layer or from a functionally diverse community from elsewhere and studying the GHG production and how it is linked with changes in overall microbial community composition. The idea of study is brilliant in its simplicity, and the work is a logical continuation from previous work of the main author and the team. Inclusion of all main permafrost types makes the findings representative of large parts of the permafrost region. Importantly, the possibility of functional limitation by low abundance or complete lack of microbes has been broadened from N_2O production and methanogenesis - mediated by limited number of organisms - to overall heterotrophic activities. These findings have strong implications for predicting permafrost-climate feedback since they suggest that not only CH_4 production but also CO_2 production may be underestimated in incubation studies with permafrost soils. The methodology is rather basic, but well suitable for testing the hypotheses. The experiments have been designed and conducted carefully, paying attention to possible methodological artefacts. Writing is concise and clear, making the work accessible and interesting for broad readership, and it is well suitable for publishing in Nature Communications. Below are a few comments that I would like to ask the authors to consider, mostly minor ones.

MAJOR COMMENT
Line 105: Microbial community coalescence is a very central concept for this study and should be clearly defined already at first occurrence. Now the references to Rillig et al. 2015, 2016 are given only in the discussion, line 470, but should be given already here.
line 124: The soil layer used for active layer inocula is very shallow, top 15 cm, and represents oxic community unless the active layer was water-logged to the top. Thus, it does not represent the whole active layer, where redox changes towards deeper layers of the active layer, and so does the microbial community composition. Please acknowledge this depth variability in active layers, and which implications that might have for the results shown here vs. real environment.

MINOR COMMENTS
Line 28: N_2O production was increased in Tussock and Palsa, but not in Cryoturbated or Yedoma. Please modify the sentence to accurately represent this.

line 157: I assume incubations were done at field moisture content, is that correct? This should be specifically mentioned, because the water content is commonly adjusted in such incubations.

Line 240: Only anoxic incubation is mentioned here, not oxic, is this intentional? Data from oxic incubation is shown too.

Line 327: What is the basis for testing anoxic data with repeated measures ANOVA but not oxic, should not the same analysis be used for both data which are essentially similar?

Line 345-346: The results section now starts with results related to possible methodological artefacts. While it is very important to report them, I would recommend starting from the main results, GHG production and microbial results.

Line 388: It is not easy to get a good overview about the results presented here. Now, each individual soil is discussed separately, but would it be possible to provide a summary about the overall pattern across all soils? That would be very useful.

Line 399: Does 400 ppb refer to the detection limit here? In the methods 100 ppb is mentioned.

Lines 471-476: Selection and die-off during frozen conditions definitely play a role here, but how about legacies from the prevailing conditions prior to permafrost aggradation (e.g., drainage; see panarctic incubation meta-analysis by Treat et al. 2015, doi: 10.1111/gcb.12875)? Please discuss.

Lines 496-498: How about water content, was that an important factor for determining whether coalescence happened or not and to which extent (similar vs. very different moisture content in both inocula and recipient soil)?

line 508: full stop missing

line 546: ‘organic N’ probably refers to TDN, that is, to water-extractable organic N? Please give reference to the supplementary figure showing the data.

line 565: Yes, it would be definitely important to account for increased functional potential, but not easy achieve, not at all. What would be your suggestion for how this could be achieved?

lines 573-576: This could be broadened still: What does this mean for model simulations? And what are the implications for interpreting meta-analyses of soil incubation data from permafrost-affected soils? I feel the interpretation might be even too modest here, considering the importance of the results.

Reviewer #2

(Remarks to the Author)
The article by Montex et al. describes the results of two laboratory experiments (oxic and anoxic), in which permafrost samples from various locations were inoculated with microbial communities from the active layer of different permafrost soils and topsoil from Central Europe. The samples were then incubated for approximately one year (oxic experiment) or six months (anoxic experiment). During this time, the concentrations of CO_2, CH_4 and N_2O were repeatedly measured, the abundance of the microbial community was determined and the functional genes of the nitrogen cycles were quantified. Introducing new microbial communities increased microbial CO_2 production in all oxic incubations and affected N_2O production in a few samples. In the sample incubated under anoxic conditions, introducing new communities caused an increase in methane production and a decrease in CO_2 production. Inoculation affected microbial community composition and functional genes, but the effects were inconclusive.
Limitations of microbial functions in permafrost have been repeatedly described, e.g. in a previous manuscript of the authors (https://doi.org/10.1038/s41561-020-00662-4) and studies cited in this manuscript. The most consistent effect of inoculation in this study was a significant increase in CO_2 production in all oxic treatments. While the authors report this increase, they do not discuss potential reasons for it. The data on N_2O production are less impactful since N_2O concentrations were below the detection limit during most measurements, and the results on anoxic methane production are confirming results from studies that the authors cite. While there is little discussion on the reasons for the increase in CO_2 production after inoculation, the discussion on the nitrogen cycle is largely speculative since the data do not support unequivocal conclusions. In this context, the manuscript confirms observations from earlier studies and supports the importance of missing microbial functions in thawing permafrost rather than introducing entirely new concepts.
I have substantial reservations about some of the approaches used by the authors, e.g. how CO_2 was measured and quantified in the oxic incubations and how gas production rates were calculated from single-concentration measurements. Furthermore, the description of the methods, particularly of the two incubation experiments, can be substantially improved (see specific comments).
Lastly, I strongly advise against using the same name for different things (e.g. 'cryoturbated' for both a permafrost sample from a cryosol and the extracted microbial community from the active layer of a cryosol), as this is confusing for the reader.
Specific comments:
L79: Cryoturbation is an important process of active layer mixing as well.
L80f: Unclear, why active layer communities may not be established in thawing permafrost. We currently have no data on this and the establishment of new communities is certainly also a matter of time.
L87ff: This sentence is somewhat misleading. The presence or absence of certain biogeochemical processes in thawing permafrost, as well as in all other soils, depends not only on the presence or absence of the respective microbial communities conducting these processes, but also on environmental factors such as the presence or absence of electron acceptors like oxygen or nitrate.
L93-103: The information on the incubation experiment should be moved to the 'Materials and Methods' section. Furthermore, the terms 'negative control' and 'positive control' in the description of the incubation experiment are somewhat misleading. I understand that the 'negative controls' are permafrost samples without inoculation, whereas all the other treatments involve inoculation from different soils or layers. Hence, the terms 'negative', 'positive' and 'control' are misleading.
L102ff: The wording of the hypothesis could be improved. 1) It is the absence of microbial functions, rather than the presence of microbial communities, that limits biogeochemical processes. 2) What does 'stimulate' mean in this context? If a microbial

function is missing, introducing it may restore this function. 3) It not clear how a 'microbial community coalescence event' may be observed other than by reestablishing missing microbial functions. In this case h3 is included in h2.
L109ff: Please indicate the depth at which the permafrost samples were collected.
Figure 1: The colours used to represent the different treatments are not self-explanatory. If I understand correctly, they represent the permafrost sample in panel A and the inoculum of the active layer in panel B. Panel b states that N_2O and CH_4 fluxes were measured five times, but I assume concentrations were measured.
L139–142: This is a repetition of information from the introduction and can be deleted here.
L143 ff: I suggest not using the terms 'positive' and 'negative' control; see the comment above.
L150 ff: Does this mean that no samples were incubated without inoculum, as in the first incubation? Please explain how the active layer samples were sterilised, and how the inactivity of the sterilised inoculum was tested.
L153 ff: Please indicate that this is the description of the oxic incubation.
L175: This headline is confusing, as all the preceding paragraphs describe the oxic experiment.
L200: Could you please explain how you measured gas samples in a syringe using the EGM-5 IRGA? The EGM-5 IRGA is designed for measuring gas concentrations in a flow-through system, rather than in discrete, small samples.
L205 ff: Sved as CO_2 in tubstantial underestimation of CO_2 production occurs if only the CO_2 in the headspace is considered and not the dissolved inorganic carbon in the water, particularly at pH values above 7, where the DIC amount in the water may be higher than the CO2 amount in the headspace.
L212ff: This paragraph describes the analysis of CH_4 and N_2O concentrations, but does not explain how production rates were determined. Furthermore, the times at which the 'flux' measurements were taken do not correspond with those given in panel b of Fig. 1.
L315ff: I assume that the statistical analysis was performed on both the oxic and anoxic experimental data. Please explain.
L323: Does this paragraph relate to the oxic experiment data? Panel b of Fig. 1 gives five times for N_2O and CH_4 measurements; here, only four are given.
It is unclear to me why four measurements prevent the calculation of total N_2O production. Furthermore, lines 212 ff state that N_2O and CH_4 fluxes were measured on certain days, but here it is stated that the fluxes could not be determined since the concentrations were below the detection limit. This paragraph states that N_2O could only be measured on one day during the palsa soil incubations. How are fluxes calculated from a single concentration measurement? In the supplements, N_2O fluxes from the Alaskan tundra permafrost are also presented.
L327: What about the N_2O data in the anoxic experiment?
Fig. 2: I suggest using unambiguous designations for sites and treatments. The authors use 'cryoturbated', 'tussock', etc. for both sites and treatments, which is confusing. I suggest clearly differentiating between the naming of soil samples (e.g. 'tussock permafrost') and the naming of treatments (e.g. 'tussock active layer inoculum'). Furthermore, I suggest omitting the individual measurements and only including the SD for better visibility. The flux rates of N_2O in panel c are unclear. The unit on the y-axis relates to a rate, but the figure caption and the text below panel c relate to concentrations.
L476: The age of the permafrost samples should be reported to evaluate this statement.
L491f: Repetition from L468.
L544f: I understand that this discussion is about the results from the aerobic experiment, which suggests that denitrification is probably not very important.
Supplement Fig. S3: This graph is very difficult to understand.

Reviewer #3

(Remarks to the Author)
This manuscript addresses a highly topical and scientifically important question regarding microbial functional limitations in permafrost soils and the potential for active layer microbial communities to alleviate these limitations via microbial community coalescence. It thus tackles a crucial aspect of permafrost thaw feedbacks to climate change, as microbial-driven greenhouse gas emissions from thawing permafrost represent one of the largest expected feedbacks to climate change from natural ecosystems. The integration of microbial ecology, functional gene quantification, biogeochemistry, fluxes and controlled incubation experiments across different permafrost soil types (including Yedoma and Holocene deposits) is laudable and represents a novel and timely contribution to Arctic microbial ecology and biogeochemical research.
Particularly the concept of microbial community coalescence in thawing permafrost is gaining attention, yet empirical demonstrations linking microbial coalescence events with functional outcomes (CO2, N2O, CH4 production) are rare. This study fills thus an important knowledge gap by systematically testing multiple soils and inocula, showing consistent functional limitations and varying degrees of alleviation by inoculation with active layer communities or a functionally diverse positive control. The approach of simulating microbial transfer via thaw disturbance or bioturbation is highly relevant to realistic thaw scenarios.
Also, the authors measure not only CO2 and CH4 but also N2O fluxes, inorganic nitrogen pools, and functional gene abundances particularly involved in nitrification and denitrification pathways. This broad functional and molecular characterization clearly strengthens mechanistic interpretations.

Despite these strong points, there are important areas needing attention to improve the manuscripts clarity, and overall impact, which need to be adressed in major revisions:

1. An issue throughout the manuscript is the repeated interpretation of functional change as being directly linked to shifts in microbial community composition. I am concerned about the strength of the evidence supporting this link. While treatment effects are statistically tested within individual datasets, the manuscript primarily demonstrates co-occurrence of changes in community composition and biogeochemical responses and discussed them qualitatively. Quantitative cross-dataset analyses that directly test correlations between microbial and functional changes are lacking. If the central claim is that

community changes drive functional changes, the authors should clarify how strong this evidence is and discuss the limitations of inferring causality from the current analyses. Adding e.g. multivariate statistic results (e.g., redundancy analysis, co-inertia analysis) or correlation coefficients would strengthen claims about microbial drivers of function and help identify potential key taxa or functional guilds responsible.

2. N2O fluxes and connection with genes are a bit messy. Though this is in the nature of N2O and often the problem with incubations, the interpretation could be improved. The low concentration of N2O in many samples suggest that N2O reduction and complete denitrification could play a role (especially under anoxic conditions, or for water saturated peat soils this could be the case), though this fact does not receive much attention in the MS. Second, nirS and nosZ genes were similar across treamtents, and the author argue for nitrification as the main source of increased N2O production, supported by the introduction of archaeal amoA genes and nitrate accumulation concomitant with inoculation. This is a bit contradictory to the literature, since denitrifies are known to play an important role in N2O production in many arctic soils. It is also a bit contradictory to their own data since the fact that in many cases N2O concentration was reduced and below detection limit suggested that complete denitrification is driving N2O fluxes here. Also, nitrifiers do generally not release N2O in high amounts (and high amounts were occasionally detected after inoculation, though unfortuantely not always significant due to low samples size). Caveats: no RNA or transcriptomic data are provided which could give evidence of actual gene expression/activity. Bacterial amoA genes were not detected (primer problem? The dominance of archaeal ammonia oxidizers in so many different permafrost soils would be important as such and would deserve more explicit discussion in the context of existing literature), primers do not cover comammox Nitrospira, known to be relevant in Arctic soils. Gross nitrification rates were not measured (e.g. with pool dilution method). The text should explicitly acknowledge these limitations and discuss how future RNA-based or isotopic tracing studies would be critical to confirm functional pathways and their relative contributions to N2O fluxes. Overall, the interpretation of the N2O data is overstated relative to the data. Statistical support for treatment effect is limited. Alternative explanations cannot be ruled out. The conclusion that processes governing N2O production are affected by function limitations is overstated and needs to be toned down.
3. The strong increase in CH4 production following active layer inoculation of palsas is on the other side a robust and well-supported result. Saying here that there is functional limitation (without on-off switch) is reasonable. However, it bears me why functional genes related to CH4 production and oxidation were not studied to clearly link this process to changes and community structure and corroborate the main narrative of the MS? Despite extensive gene analysis, no methanogen-specific marker (e.g. mcrA) or taxon-level analysis were presented? The reference to Candids Mathanofloresn is good but relies on external studies rather than observations from the present one. Therefore, even here, the mechanistic interpretation of inoculation/microbial-driven methanogen introduction remains speculative and indirect.

4. For CO2, a clear functional limitation on CO2 production was observed in all permafrost soils, ranging from 20-60% across all soils, though increase with native community was less clear. The explanation of why sometimes coalescence was successful and sometimes not is clear and convincing. since CO2 is the main GHG, this is an important result.

5. The Cryoturbated permafrost soil consistently yielded low DNA quantities and scarce functional gene detection, leading the authors to exclude it from some analyses. While transparency is good, discussion surrounding how this affects interpretation of functional limitations across soil types, and whether methodological constraints might bias conclusions, should be expanded. Are there alternative methods or deeper sequencing approaches that could have resolved these issues?
6. Given the importance of coalescence to biogeochemical outcomes, a more nuanced discussion of possible drivers (e.g., soil physicochemical properties, microbial traits, spatial-temporal dynamics), supported by references to recent literature, would be valuable. Additionally, the ecological relevance of the inoculation approach (how it relates to natural thaw conditions?) is worth elaborating.
7. Incubations were conducted without plants, which likely influenced particularly N dynamics and N2O fluxes by removing plant N uptake. Strong NH4 immobilization as seen here as an indicator of N limitations of microbes is often the consequence. This limitation and its implications for extrapolating to field conditions should be discussed briefly.
8. The manuscript would benefit from explicitly reporting and discussing soil C/N ratios. These ratios are central to interpreting N availability, mineralization-immobilization balance, and N2O dynamics. Differences in C/N among soils may partly explain variation in nitrification, mineralization, N2O production and gene abundances.

Specific comments:

Line 59: The transition to Yedoma is abrupt and overly specific. It would improve readability to first mention more common and widespread permafrost soil types (e.g., permafrost peatlands, uplands, cryoturbated soils) and frozen conditions, and then introduce Yedoma
Lines 87–89: The statement implying that changes in biogeochemical processes would largely depend on changes in microbial community composition is too strong. It I also contradicting the functional limitation hypothesis of a given community of the current study. Functional limitations can also be alleviated by activation of existing microbial populations via substrates, exudates, or environmental modifications introduced by interacting communities, without major compositional turnover.

Lines 105–106: The third statement reads more like an interpretation than a testable hypothesis. It would be clearer to move it to the discussion and keep the hypothesis section focused on directly testable predictions about functional limitation and restoration.

Lines 149–150: Only the palsa mire was incubated under anaerobic conditions. The authors should briefly explain this choice (e.g., future subsidence and waterlogging are particularly relevant for palsa systems).
Line 212: Consider revising to: “Production and/or consumption rates of CH_4 and N_2O…”
Lines 311–312: Was the cooling potential associated with possible N_2O uptake considered? Waterlogged anaerobic peat soils can consume atmospheric N_2O, which could offset warming from CO_2 and CH_4. Even if N_2O was below detection, assuming upper and lower bounds (e.g., detection limit vs. zero) could help frame the potential cooling effect.
Lines 323–324: Please clarify why cumulative N_2O budgets could not be calculated (e.g., limited sampling frequency).

Figure 2: Indicating statistically significant differences relative to controls directly in the figure would greatly aid reader.
Line 406:If CH_4 production increased fourfold, describing it as “limited” may be misleading; consider rephrasing.
Line 455: Please specify clearly whether a lower nir/nos ratio is meant.
Line 573: The text should also acknowledge the strong decrease observed in N_2O.
Line 574: I strongly sugget to replace “carbon-climate feedback” with “permafrost-climate feedback,” since both carbon and nitrogen processes were studied.

Version 1:

Reviewer comments:

Reviewer #1

(Remarks to the Author)
Reviewer report

The authors have provided a carefully revised version of this interesting manuscript, where both reviewers’ concerns are in my opinion properly addressed. Additional discussion, analysis, and many small but important clarifying details have been added. Limitations of the study are discussed as well as the broader implications with some suggestions for future studies. Some parts of the new text (indicated below with line numbers) appear slightly raw compared to the rest of the text and would benefit from rephrasing. The article can be published after a minor revision related to those few points.

line 33: Based on N_2O section starting on line 413, the increases in N_2O were not statistically significant. Thus, this seems like an overstatement and some rephrasing is needed.

lines 376-381: This is a nice analysis, but I find the wording here unnecessarily complicated. Would be great if you could provide a concise plain language explanation of the aims of this analysis. Was the aim to investigate which microbial classes were most important in driving the treatment differences in cumulative CO_2 production?

line 386: Consider using a full stop between ’58 data points' and 'we’ instead of a comma.

line 486-> Based on this analysis, is it possible to say which microbial classes had a positive effect and which negative on the CO_2 production? Or, do you mention only those classes which had a positive effect? Please clarify this.

line 487: Soil chemistry is mentioned here, but no results regarding soil chemistry are reported in this section. It remains unclear if and how they were used. Please clarify.

line 494: Present verb (correlate) within a text written in past tense.

lines 499-502: A long and complicated sentence hard to understand; please revise for better clarity and text flow.

lines 622-625: Some rephrasing needed. The sentence is long and complicated and the meaning remains unclear.

lines 723-724: Neither of these two links seem to work?

Reviewer #2

(Remarks to the Author)
The authors thoroughly revised the manuscript and most of my initial concerns are considered in the revised manuscript. I have only some minor comments/suggestions.
However, some of my previous concerns remain. Firstly, rather than presenting a novel concept, the interesting data in this paper confirms the results of previous papers. Secondly, some of the data is interpreted overly speculatively, particularly with regard to the nitrogen cycle.
Specific comments:
L104f: unclear what positive and negative controls mean
L173: ‘increase’
L217ff: Flushing does not bring CO_2 concentrations to zero, at least as long as there is still CO_2 dissolved in the sample water. Please rephrase. Ignoring the remaining CO_2 in the flasks would result in an overestimation of gas production,

ignoring the dissolved inorganic carbon in the water would cause an underestimation of the gas production rates. Both should be mentioned. Not very clear to me, why you choose to ignore DIC in the incubations, you should have all the data needed to account for it.

L560ff: I was not able to extract the age data from the given reference since it uses these data to determine carbon accumulation rates without presenting ages in the main text. Please give the age range of the non-Yedoma permafrost as far as they are available to substantiate this conclusion.

Reviewer #3

(Remarks to the Author)

The authors have done an excellent job revising the manuscript in response to all reviewer comments. My own comments and concerns have been thoroughly addressed and satisfactorily resolved. In addition, the authors conducted further molecular analyses, which have strengthened the study and improved the overall quality of the manuscript.

Overall, the revisions have made the manuscript substantially stronger, and I have no further comments. I believe this work represents a valuable contribution to permafrost science and will be of broad interest to the community. I therefore support publication.

Open Access This Peer Review File is licensed under a Creative Commons Attribution 4.0 International License, which permits use, sharing, adaptation, distribution and reproduction in any medium or format, as long as you give appropriate credit to the original author(s) and the source, provide a link to the Creative Commons license, and indicate if changes were made.

In cases where reviewers are anonymous, credit should be given to 'Anonymous Referee' and the source.

The images or other third party material in this Peer Review File are included in the article’s Creative Commons license, unless indicated otherwise in a credit line to the material. If material is not included in the article’s Creative Commons license and your intended use is not permitted by statutory regulation or exceeds the permitted use, you will need to obtain permission directly from the copyright holder.

To view a copy of this license, visit https://creativecommons.org/licenses/by/4.0/

REVIEWER COMMENTS

Reviewer #1 (Remarks to the Author):

Review report on manuscript by Monteux et al.

GENERAL COMMENT

Here, the authors have presented a thrilling study, where they investigated occurrence of microbial limitations for CO2, CH4 and N2O production in permafrost. This was achieved by inoculating permafrost with inocula from active layer or from a functionally diverse community from elsewhere and studying the GHG production and how it is linked with changes in overall microbial community composition. The idea of study is brilliant in its simplicity, and the work is a logical continuation from previous work of the main author and the team. Inclusion of all main permafrost types makes the findings representative of large parts of the permafrost region. Importantly, the possibility of functional limitation by low abundance or complete lack of microbes has been broadened from N2O production and methanogenesis - mediated by limited number of organisms - to overall heterotrophic activities. These findings have strong implications for predicting permafrost-climate feedback since they suggest that not only CH4 production but also CO2 production may be underestimated in incubation studies with permafrost soils. The methodology is rather basic, but well suitable for testing the hypotheses. The experiments have been designed and conducted carefully, paying attention to possible methodological artefacts. Writing is concise and clear, making the work accessible and interesting for broad readership, and it is well suitable for publishing in Nature Communications. Below are a few comments that I would like to ask the authors to consider, mostly minor ones.

We thank the reviewer for their appreciative and constructive feedback of our manuscript, and answer their comments below in blue font.

MAJOR COMMENT

Line 105: Microbial community coalescence is a very central concept for this study and should be clearly defined already at first occurrence. Now the references to Rillig et al. 2015, 2016 are given only in the discussion, line 470, but should be given already here.

Thank you for this suggestion, we now refer to Rillig *et al.* 2015, 2016 at first mention of coalescence (L113).

line 124: The soil layer used for active layer inocula is very shallow, top 15 cm, and represents oxic community unless the active layer was water-logged to the top. Thus, it does not represent the whole active layer, where redox changes towards deeper layers of the active layer, and so does the microbial community composition. Please acknowledge this depth variability in active layers, and which implications that might have for the results shown here vs. real environment.

Indeed, active layer communities may vary substantially over depth, especially if there are steep redox gradients. We now clarify the choice of a topsoil inoculum in the methods (L133-135), and the variability over depth in the discussion (L607-609).

MINOR COMMENTS

Line 28: N2O production was increased in Tussock and Palsa, but not in Cryoturbated or Yedoma. Please modify the sentence to accurately represent this.
We rephrased this part of the abstract to reflect this nuance (L33-34).

line 157: I assume incubations were done at field moisture content, is that correct? This should be specifically mentioned, because the water content is commonly adjusted in such incubations.
Indeed, incubations were done near field capacity. All permafrost soils contained excess water from ground ice which was decanted prior to weighing the soils into flasks. We clarified this point (L166-167).

Line 240: Only anoxic incubation is mentioned here, not oxic, is this intentional? Data from oxic incubation is shown too.
We thank the reviewer for spotting this mistake, which has now been corrected (L257). This instead refers to the oxic incubations as we initially did not carry out molecular analyses on the anoxic incubation.

Line 327: What is the basis for testing anoxic data with repeated measures ANOVA but not oxic, should not the same analysis be used for both data which are essentially similar?
The basis is practical: higher rates and longer duration mean there were too many observations relative to the number of experimental units to carry out repeated measures ANOVA, although we would have preferred this more elegant approach. With 4 replicates and 3 to 5 treatments, we could not carry out Mauchly's test for sphericity or Greenhouse-Geiser sphericity corrections in excess of 10 measurement points, while the entire dataset contained 21 measurement points. We explored linear mixed effects models as an alternative way to specify repeated measurements within a jar, but could only obtain singular fits, with minute random effects. We therefore decided to use linear models with Inoculum, Time, their interaction, and Flask as fixed effects – to assess whether an Inoculum:Time interaction or the experimental unit itself had to be considered in interpretation. This allowed us to focus on the cumulative CO_2 produced over the whole incubation, which is ideal since we do not have supporting data at the temporal resolution of CO_2 fluxes to explain or discuss them beyond speculation. We have now added the summary statistics of one-way ANOVAs on linear models for each soil in Supplementary Figure S1.

Line 345-346: The results section now starts with results related to possible methodological artefacts. While it is very important to report them, I would recommend starting from the main results, GHG production and microbial results.
We have now moved this section further down in the Results section and reordered the supplementary display elements accordingly.

Line 388: It is not easy to get a good overview about the results presented here. Now, each individual soil is discussed separately, but would it be possible to provide a summary about the overall pattern across all soils? That would be very useful.
We have now added a sentence to describe the overall N_2O results (L420-422): *"Overall, N_2O was below detection limit in most soils regardless of*

inoculation, with detections mostly occurring in soils inoculated with Multifunctional (Tussock-PF, Palsa-PF) and Palsa-AL (Palsa-PF)."

Line 399: Does 400 ppb refer to the detection limit here? In the methods 100 ppb is mentioned.
This stems from small differences in the analytical approaches in the different laboratories where we conducted the oxic and anoxic incubation: in the oxic incubation our lowest reference gas was 400 ppb (L235) but in the anoxic incubation this was 100 ppb (L331).

Lines 471-476: Selection and die-off during frozen conditions definitely play a role here, but how about legacies from the prevailing conditions prior to permafrost aggradation (e.g., drainage; see panarctic incubation meta-analysis by Treat et al. 2015, doi: 10.1111/gcb.12875)? Please discuss.
This is a good point, which our setup cannot discriminate between. While we assume selection, drift and die-off to be predominant forces in the permafrost environment, it is entirely possible that some taxa and functions were initially absent, and that water-logging in particular, was a strong driver towards distinct communities. We note that beyond the snapshot of microbial communities initially differing, the water saturation at the time of permafrost aggradation may further affect ice and massive ice content, and thereby the downstream selection processes. We have now added some discussion of this and references to Treat *et al.*, 2015, Holm *et al.*, 2020, Barbato *et al.*, 2022 and Waldrop *et al.*, 2023 (L556-560).

Lines 496-498: How about water content, was that an important factor for determining whether coalescence happened or not and to which extent (similar vs. very different moisture content in both inocula and recipient soil)?
Water content did not explain these differences, for instance largely wetter inoculum soils did (Palsa-AL→Yedoma-PF ΔMOISTURE = +40%, Palsa-AL→Palsa-PF +51%, Tussock-AL→Yedoma-PF +42%) or did not (Tussock-AL→Tussock-PF +41%) coalesce, while similarly dry inoculum did (Multifunctional→Palsa-PF -3%) or did not (Cryoturbated-AL→Cryoturbated-PF +2%) coalesce. Likewise, wetter (Yedoma, Tussock-PF) or drier (Cryoturbated-PF, Palsa-PF) permafrost did not explain success or failure of coalescence events.
We have now added moisture to the list of factors which did not explain the findings (L583).

line 508: full stop missing
Thank you, fixed.

line 546: 'organic N' probably refers to TDN, that is, to water-extractable organic N? Please give reference to the supplementary figure showing the data.
"*Organic N*", here and throughout, refers to the difference between water-extractable TDN and the sum of inorganic N, as mentioned in methods at L250-251. We now added a reference to the reworked Supplementary Figure S5 which breaks down the dissolved N species in each sample to emphasize this increase in organic N.

line 565: Yes, it would be definitely important to account for increased

functional potential, but not easy achieve, not at all. What would be your suggestion for how this could be achieved?
We would recommend actively inoculating future permafrost incubations with active layer leachates, or even mixing active layer soil into the permafrost, to simulate abrupt thaw processes.
When it comes to the wealth of existing incubation-based data, we have no one-size-fits-all solution. Ideally, further work on the spatial variability of functional limitations will allow to generalize observations across broad groups of permafrost, either by edaphic characteristics, aboveground vegetation or deposition history. This could then be broadly related to incubation data similarly categorized, although possible differences in sterility during sampling across incubation studies need to be considered.
We now expand on this topic in the end of the discussion L689-703.

lines 573-576: This could be broadened still: What does this mean for model simulations? And what are the implications for interpreting meta-analyses of soil incubation data from permafrost-affected soils? I feel the interpretation might be even too modest here, considering the importance of the results.
We want to remain cautious , but we have now expanded on the potential implications for re-interpreting incubation data, together with the recommendations for future studies L689-703.

Reviewer #2 (Remarks to the Author):

The article by Montex et al. describes the results of two laboratory experiments (oxic and anoxic), in which permafrost samples from various locations were inoculated with microbial communities from the active layer of different permafrost soils and topsoil from Central Europe. The samples were then incubated for approximately one year (oxic experiment) or six months (anoxic experiment). During this time, the concentrations of CO_2, CH_4 and N_2O were repeatedly measured, the abundance of the microbial community was determined and the functional genes of the nitrogen cycles were quantified. Introducing new microbial communities increased microbial CO_2 production in all oxic incubations and affected N_2O production in a few samples. In the sample incubated under anoxic conditions, introducing new communities caused an increase in methane production and a decrease in CO_2 production. Inoculation affected microbial community composition and functional genes, but the effects were inconclusive.
Limitations of microbial functions in permafrost have been repeatedly described, e.g. in a previous manuscript of the authors (https://doi.org/10.1038/s41561-020-00662-4) and studies cited in this manuscript. The most consistent effect of inoculation in this study was a significant increase in CO_2 production in all oxic treatments. While the authors report this increase, they do not discuss potential reasons for it. The data on N_2O production are less impactful since N_2O concentrations were below the detection limit during most measurements, and the results on anoxic methane production are confirming results from studies that the authors cite. While there is little discussion on the reasons for the increase in CO_2 production after inoculation, the discussion on the nitrogen cycle is largely speculative since the data do not support unequivocal conclusions. In this context, the manuscript confirms observations from earlier studies and supports the

importance of missing microbial functions in thawing permafrost rather than introducing entirely new concepts.
We note the concerns about the novelty of our study. As we point out throughout the text, previous studies on functional limitations in permafrost – which we clearly acknowledge – have been carried out in Yedoma permafrost. Our earlier findings faced the recurring critique that Yedoma permafrost is an oddity, owing to the extreme age of these deposits, compared to Holocene deposits representing 70% of permafrost SOC. Consequently, the main goal of our study was to address that critique, and this is reflected in the experimental design. The novelty in our study consists in:
a) expanding the framework of functional limitations to major Holocene permafrost deposits,
b) demonstrating that functional changes require community coalescence events,
c) showing that functional limitation of methanogenesis owes not simply to the presence-absence of methanogens (as in literature) but to topsoil methanogens in particular, and finally
d) speculating on mechanisms underlying the drastic changes observed in terms of N-cycling.

We have now clarified the speculative nature of some of the N-cycling discussion, as well as added new analyses and discussion of taxa associated with the increases in CO_2 production (see more details below in our response to reviewer #3 on this point).

I have substantial reservations about some of the approaches used by the authors, e.g. how CO_2 was measured and quantified in the oxic incubations and how gas production rates were calculated from single-concentration measurements. Furthermore, the description of the methods, particularly of the two incubation experiments, can be substantially improved (see specific comments).
We have improved the description of the methods throughout the text and address specific concerns on CO_2 measurements in responses to comments below.

Lastly, I strongly advise against using the same name for different things (e.g. 'cryoturbated' for both a permafrost sample from a cryosol and the extracted microbial community from the active layer of a cryosol), as this is confusing for the reader.
We have now clarified the naming of soils and treatments throughout the text and display elements, which we agree improve readability.
To prevent ambiguities, the permafrost soils have been relabeled as *-PF and the active layer inoculums as *-AL. Moreover, the negative control has been renamed Control, and the positive control has been renamed Multifunctional. We believe these changes should rule out any misunderstandings and address the reviewer’s concerns.

Specific comments:
L79: Cryoturbation is an important process of active layer mixing as well.
Indeed, we have now rephrased to mention cryoturbation (L86).

L80f: Unclear, why active layer communities may not be established in

thawing permafrost. We currently have no data on this and the establishment of new communities is certainly also a matter of time.
Some studies in long-term permafrost thaw manipulations in the field (e.g. Monteux *et al.,* 2018, Doherty *et al.,* 2020, Johnston *et al.,* 2019) suggest establishment of new communities upon thaw within a few years or a decade. Those studies do not distinguish between coalescence and endogenous changes, there is therefore indeed very little data on coalescence *per se*. Doherty *et al*., 2025 show that different permafrost soils exhibit different coalescence dynamics upon mixing with active layer, e.g. which PF:AL ratio was needed to resemble AL communities. In our study we used much smaller amounts of microorganisms in our inoculums, to avoid increasing total microbial biomass in the process. Copiotrophs from the active layer should thrive in DOC-rich newly-thawed permafrost and outcompete slow-growing recyclers present under frozen conditions. There are likely also dormant copiotrophs in permafrost that become active under favourable conditions. Active layer microorganisms therefore need to outcompete those already present, and together with putative differences in soil chemistry, this may impede coalescence. We have clarified this by mentioning competition dynamics (L88-89).

L87ff: This sentence is somewhat misleading. The presence or absence of certain biogeochemical processes in thawing permafrost, as well as in all other soils, depends not only on the presence or absence of the respective microbial communities conducting these processes, but also on environmental factors such as the presence or absence of electron acceptors like oxygen or nitrate.
We fully agree and did not intend to minimize the role of thermodynamic constraints. To reflect our reasoning this sentence should have included a mention of *all things equal otherwise*, and while we measured a range of soil conditions we cannot ascertain that only microbial community composition was modified by inoculation. This sentence was also confusing to reviewer #3 and was not essential to the reasoning of the introduction, we have removed it and this paragraph now ends with the knowledge gap.

L93-103: The information on the incubation experiment should be moved to the 'Materials and Methods' section. Furthermore, the terms 'negative control' and 'positive control' in the description of the incubation experiment are somewhat misleading. I understand that the 'negative controls' are permafrost samples without inoculation, whereas all the other treatments involve inoculation from different soils or layers. Hence, the terms 'negative', 'positive' and 'control' are misleading.
Due to the formatting of Nature Communications articles, the Material and Methods section will appear after the Results and Discussion, we therefore chose to briefly summarize the experimental design at this point for ease of reading. However, we are willing to modify this at the editor's discretion.
We have now renamed the controls to 'Control' – negative control using sterile water – and 'Multifunctional' – temperate grassland inoculum considered as a positive control.

L102ff: The wording of the hypothesis could be improved. 1) It is the absence of microbial functions, rather than the presence of microbial communities, that limits biogeochemical processes. 2) What does 'stimulate' mean in this

context? If a microbial function is missing, introducing it may restore this function. 3) It not clear how a 'microbial community coalescence event' may be observed other than by reestablishing missing microbial functions. In this case h3 is included in h2.

We have now clarified the hypotheses to more directly refer to functions. Moreover, as pointed out also by reviewer 1, statement 3 is no longer an hypothesis and has been rephrased as an expectation flowing from our suggested mechanism instead. The hypothesis section (L108-115) now reads as follows:

We hypothesize that 1) missing functions in microbial communities limit biogeochemical processes in all permafrost types – although older permafrost deposits may be more strongly affected –, 2) active layer microbial communities can restore missing or stimulate hampered processes by introducing missing functions. We further anticipate that microbial community coalescence events (that is, the interchange of entire microbial communities upon 'invasion' or 'establishment', sensu refs. [Rillig2015,2016]) should be observed wherever functional limitations are detected, while failing to induce coalescence would suggest that microorganisms with missing functions could not establish.

L109ff: Please indicate the depth at which the permafrost samples were collected.

The depths at which the different permafrost samples were collected are mentioned at lines 125, 128, 130 and 132.

Figure 1: The colours used to represent the different treatments are not self-explanatory. If I understand correctly, they represent the permafrost sample in panel A and the inoculum of the active layer in panel B. Panel b states that N_2O and CH_4 fluxes were measured five times, but I assume concentrations were measured.

You understand correctly. Nevertheless, in addition to renaming the soils and treatments unambiguously, we have now reworked all display elements to convey the information more clearly. We have also corrected the inconsistency in the number of N_2O/CH_4 concentration measurements used to calculate net fluxes in Figure 1.

L139–142: This is a repetition of information from the introduction and can be deleted here.

We have rephrased this sentence to limit repetition (L148-149).

L143 ff: I suggest not using the terms 'positive' and 'negative' control; see the comment above.

This has now been modified accordingly throughout the text and display elements.

L150 ff: Does this mean that no samples were incubated without inoculum, as in the first incubation? Please explain how the active layer samples were sterilised, and how the inactivity of the sterilised inoculum was tested.

This control had a sterilized inoculum rather than water. The sterilization (gamma irradiation at 40 kGy) is explained in further details in Supplementary Text. The anoxic incubation happened after we secured initial results from the experiment shown in Supplementary Figure S6 on the potential artefacts from

inoculation. As we could demonstrate that sterilization resulted in no visible effect of inoculation, we felt confident in using such an inoculum as negative control for this experiment.

L153 ff: Please indicate that this is the description of the oxic incubation.
We now clarify this (L162).

L175: This headline is confusing, as all the preceding paragraphs describe the oxic experiment.
To clarify, we have renamed the previous section to "*Soil sampling and overall experimental design*", this section to "*Methods specific to the oxic experiment*", and the next section to "*Methods specific to the anoxic experiment*".

L200: Could you please explain how you measured gas samples in a syringe using the EGM-5 IRGA? The EGM-5 IRGA is designed for measuring gas concentrations in a flow-through system, rather than in discrete, small samples.
We used the *static sampling* mode of EGM-5 for measuring the concentration of small sample volumes: the pump is turned off and samples are manually injected. This measurement process is a standard feature of the instrument suggested by the manufacturer. While 5 ml are sufficient to fill in the sampling loop, we used 10-15 ml to ensure the loop was fully flushed out and residual CO_2 was not carried over from sample to sample. We now clarify this (L211-217).

L205 ff: Sved as CO_2 in tubstantial underestimation of CO_2 production occurs if only the CO_2 in the headspace is considered and not the dissolved inorganic carbon in the water, particularly at pH values above 7, where the DIC amount in the water may be higher than the CO2 amount in the headspace.
We only had pH values slightly above 7 in the Cryoturbated soil (Supplementary Figure S4), but perhaps more importantly, there were no differences in pH across treatments within a soil. This suggests that dissolved carbonates would not have affected our interpretation of effects between treatments, although they may affect total carbon budgets. We now acknowledge this in L225-227.

L212ff: This paragraph describes the analysis of CH_4 and N_2O concentrations, but does not explain how production rates were determined. Furthermore, the times at which the 'flux' measurements were taken do not correspond with those given in panel b of Fig. 1.
We apologize for the mistake in measurement dates in this sentence: the dates in Figure 3 are correct. Due to only 2 samples out of 58 having detectable N_2O at day 187 we did not carry out the measurement at day 389 that was initially planned and previously mentioned here and in Figure 1. This has now been corrected here (L228-230) and in Figure 1.
Net production of CO_2, CH_4 and N_2O from concentration measurements are calculated as described for CO_2 in the oxic experiment. Gas production rates can be derived from single concentration measurements because the flasks were flushed with CO_2-, N_2O- and CH_4-free synthetic gas mixes after each measurement. The extensive flushing (10 × headspace volume) ensures a zero-endpoint in addition to the concentration measurement, the ideal gas law

is used to calculate the quantity of gas present at the measurement time, and the rate is obtained by dividing that quantity by the time since zeroing.
As mentioned in Supplementary Figure S1, we are aware that the relationship is not directly proxying biogenic production as diffusion from the soil pores and porewater to the headspace is accelerated immediately after flushing due to the increased gradient to a GHG-free headspace. This is reflected in the higher estimated rates after shorter production windows at days 187 and 352, but these biases in diffusion dynamics affect all inoculation treatments indiscriminately.
We have made changes throughout this section (L210-237) to clarify these points.

L315ff: I assume that the statistical analysis was performed on both the oxic and anoxic experimental data. Please explain.
The different statistical approaches for anoxic and oxic flux data are described in text. We could not use the more elegant RM-ANOVA approach in the oxic incubation, due to a too high number of measurements within each experimental unit. We instead used one-way ANOVAs on cumulative CO_2 produced integrated over the 389 days, first (as described in text) because we do not have other data matching the temporal resolution of CO_2 fluxes which could be used to explain or discuss them beyond speculation, and second because we could rule out Treatment:Time interactions through linear models. We have now included summary statistics of these linear models in Supplementary Figure S1, see also our response to reviewer #1 above on this topic.

L323: Does this paragraph relate to the oxic experiment data? Panel b of Fig. 1 gives five times for N_2O and CH_4 measurements; here, only four are given.
Yes, we did not carry out the last measurement at day 389, since only 2 samples were above detection limit at day 187. We have now corrected this discrepancy.

It is unclear to me why four measurements prevent the calculation of total N_2O production.
We now clarify (L228-230 and 354-356) that these measurements only cover the net production between the previous flushing (for CO_2 measurement) and this date, depicting the net flux for these time periods (days 2-7, 7-17, 33-40 and 180-187, but preventing from calculating a budget over the whole incubation. We now depict the N_2O and CH_4 sampling events as well as the net production period they cover in Supplementary Figure S1.

Furthermore, lines 212 ff state that N_2O and CH_4 fluxes were measured on certain days, but here it is stated that the fluxes could not be determined since the concentrations were below the detection limit. This paragraph states that N_2O could only be measured on one day during the palsa soil incubations. How are fluxes calculated from a single concentration measurement? In the supplements, N_2O fluxes from the Alaskan tundra permafrost are also presented.
N_2O could be measured on more occasions than just one day in the Palsa soil, data points in Figure 3c show all measurements above 200ppb (0.5×detection limit). Only in the Palsa soil at day 7 was there a sufficient number of measurement above the detection limit to allow statistical testing of the

results – the rest of the soil:dates combination are solely described and cannot be subjected to statistical analyses. We have now rephrased this section to clarify this point (L356-359).

L327: What about the N_2O data in the anoxic experiment?
We mention at L331-332 that N_2O remained below detection limit throughout the anoxic experiment.

Fig. 2: I suggest using unambiguous designations for sites and treatments. The authors use 'cryoturbated', 'tussock', etc. for both sites and treatments, which is confusing. I suggest clearly differentiating between the naming of soil samples (e.g. 'tussock permafrost') and the naming of treatments (e.g. 'tussock active layer inoculum'). Furthermore, I suggest omitting the individual measurements and only including the SD for better visibility. The flux rates of N_2O in panel c are unclear. The unit on the y-axis relates to a rate, but the figure caption and the text below panel c relate to concentrations.
As mentioned in our response above, we have now relabeled soils and treatments unambiguously.
Regarding removing individual measurements, Nature Communications editorial guidelines state that individual data points should be shown if $n<10$, but we are willing to make this change at the editor’s discretion.
Figure 2c presents rates, however the detection limit applies to concentrations in the samples, which correspond to distinct rates depending on dry weights and time between two measurements, the caption therefore mentions ppb. We now clarify this in the figure caption.

L476: The age of the permafrost samples should be reported to evaluate this statement.
The age of the SOM in the Palsa permafrost is mentioned at L681. We cannot provide dates for the permafrost as such, as we lack precise data and proxies to know when the soils were last thawed. The SOM age in the Palsa permafrost is an overly conservative estimate as the permafrost cannot be older than the SOM it contains, and this is already substantially younger than any Pleistocene-age Yedoma deposit – notably that of the CRREL tunnel which has been dated 11 to 33 ky BP. We have now added the reference dating the Palsa permafrost in this sentence L562.

L491f: Repetition from L468.
Thank you, we have now removed this repetition.

L544f: I understand that this discussion is about the results from the aerobic experiment, which suggests that denitrification is probably not very important.
Indeed, denitrification would not be very important in fully aerobic settings. Despite the oxic headspace and small soil volume, we could not absolutely rule out anaerobic microsites within the incubation flasks, and the gas (N_2) used for flushing would not allow us to detect denitrification. We have rephrased this sentence to clarify this (L635-638).

Supplement Fig. S3: This graph is very difficult to understand.
We have now simplified this figure, which following other changes is now Supplementary Figure S5: we removed the color-coding by inoculum, as well

as the bars depicting the individual breakdown of N-species per sample. Instead, mean±SE are shown along with individual values for each N-species within a given soil:inoculum combination, and the general colour-coding of panels used throughout the new display elements indicate which soil is in focus.

Reviewer #3 (Remarks to the Author):

This manuscript addresses a highly topical and scientifically important question regarding microbial functional limitations in permafrost soils and the potential for active layer microbial communities to alleviate these limitations via microbial community coalescence. It thus tackles a crucial aspect of permafrost thaw feedbacks to climate change, as microbial-driven greenhouse gas emissions from thawing permafrost represent one of the largest expected feedbacks to climate change from natural ecosystems. The integration of microbial ecology, functional gene quantification, biogeochemistry, fluxes and controlled incubation experiments across different permafrost soil types (including Yedoma and Holocene deposits) is laudable and represents a novel and timely contribution to Arctic microbial ecology and biogeochemical research.
Particularly the concept of microbial community coalescence in thawing permafrost is gaining attention, yet empirical demonstrations linking microbial coalescence events with functional outcomes (CO_2, N_2O, CH_4 production) are rare. This study fills thus an important knowledge gap by systematically testing multiple soils and inocula, showing consistent functional limitations and varying degrees of alleviation by inoculation with active layer communities or a functionally diverse positive control. The approach of simulating microbial transfer via thaw disturbance or bioturbation is highly relevant to realistic thaw scenarios.
Also, the authors measure not only CO_2 and CH_4 but also N_2O fluxes, inorganic nitrogen pools, and functional gene abundances particularly involved in nitrification and denitrification pathways. This broad functional and molecular characterization clearly strengthens mechanistic interpretations.

Despite these strong points, there are important areas needing attention to improve the manuscripts clarity, and overall impact, which need to be adressed in major revisions:
We thank the reviewer for their appreciation of our work and answer their concerns below.

1. An issue throughout the manuscript is the repeated interpretation of functional change as being directly linked to shifts in microbial community composition. I am concerned about the strength of the evidence supporting this link. While treatment effects are statistically tested within individual datasets, the manuscript primarily demonstrates co-occurrence of changes in community composition and biogeochemical responses and discussed them qualitatively. Quantitative cross-dataset analyses that directly test correlations between microbial and functional changes are lacking. If the central claim is that community changes drive functional changes, the authors should clarify how strong this evidence is and discuss the limitations of inferring causality

from the current analyses. Adding e.g. multivariate statistic results (e.g., redundancy analysis, co-inertia analysis) or correlation coefficients would strengthen claims about microbial drivers of function and help identify potential key taxa or functional guilds responsible.
We have now carried out additional analyses to this end using machine learning for variable selection prior to correlation testing. The patchiness of data such as N_2O or NO_3 would limit the use of db-RDA or CIA to a narrow subset of samples, hampering the scope of a cross-dataset analysis. We therefore focused on CO_2 because this variable was observed throughout the dataset and had comparatively little mechanistic discussion. As we therefore tested a univariate variable against a large number of potentially collinear predictors, we used a regression tree approach to variable selection prior to correlation testing, described in more details below. We have now added methods (L376-397), results (L486-502) and discussion (L613-625) to reflect these new analyses.

We used cumulative CO_2 production at day 389 detrended by soil type to account for the large differences in CO_2 release across soil types and focus on the inoculum manipulation instead. To compare the contribution of microbial community composition vs measured soil chemistry variables, we used a randomForest machine learning approach combining VSURF (variable selection using randomForest) and rfPermute (permutations of predictor variables in randomForest to assess significance of variable importance). We used relative microbial abundances at Class level, resulting in 94 bacterial classes, and chemistry variables expressed per soil dry weight and per soil organic matter. We retained only variables congruent between methods, i.e. retained at VSURF interpretation phase and with a $P<0.05$ for significance of both importance metrics of rfPermute (increase in node purity and increase in MSE). Nine variables were retained by that approach, which were all microbial classes: soil chemistry variables were not as important as bacterial classes in explaining the variation in CO_2 across samples.
To focus on general patterns, those nine classes were further tested for correlation between their relative abundance and detrended cumulative CO_2 production in each soil – omitting the Cryoturbated-PF soil where DNA quality was suboptimal – five of these nine microbial classes significantly correlated with CO_2 production in each soil type, all positively, and mainly due to being introduced by the Multifunctional and Cryoturbated-AL inocula (Acidobacteriota Subgroup5 and Holophagae, Chloroflexi Dehalococcoidia, Armatimonadota Fimbriimonadia and Crenarchaeota Nitrososphaeria).

2. N2O fluxes and connection with genes are a bit messy. Though this is in the nature of N2O and often the problem with incubations, the interpretation could be improved. The low concentration of N2O in many samples suggest that N2O reduction and complete denitrification could play a role (especially under anoxic conditions, or for water saturated peat soils this could be the case), though this fact does not receive much attention in the MS. Second, nirS and nosZ genes were similar across treamtents, and the author argue for nitrification as the main source of increased N2O production, supported by the introduction of archaeal amoA genes and nitrate accumulation concomitant with inoculation. This is a bit contradictory to the literature, since denitrifies are known to play an important role in N2O production in many arctic soils. It is also a bit contradictory to their own data since the fact

that in many cases N2O concentration was reduced and below detection limit suggested that complete denitrification is driving N2O fluxes here. Also, nitrifiers do generally not release N2O in high amounts (and high amounts were occasionally detected after inoculation, though unfortuantely not always significant due to low samples size).

We agree that the interpretation of linkages between N2O fluxes and gene abundances is challenging. Two explanations arise for not detecting N_2O upon measurement time:

- N_2O is not being produced
- There is a balance between N_2O production and consumption, either between N_2O producers and consumers or because of complete denitrification, without exceeding detection limit (at any time in the anoxic incubation, at most occasions in the oxic incubation). Our experimental design does not allow us to distinguish between these two explanations and we acknowledge that (L635-638). The effect of AOA on N_2O is likely indirect, i.e. they are not responsible for the occasional high production of N_2O but rather indirectly contribute by providing nitrate for denitrifiers.

We have reworked and nuanced the paragraph of the Discussion on N_2O results L626-666.

Caveats: no RNA or transcriptomic data are provided which could give evidence of actual gene expression/activity. Bacterial amoA genes were not detected (primer problem? The dominance of archaeal ammonia oxidizers in so many different permafrost soils would be important as such and would deserve more explicit discussion in the context of existing literature), primers do not cover comammox Nitrospira, known to be relevant in Arctic soils. Gross nitrification rates were not measured (e.g. with pool dilution method). The text should explicitly acknowledge these limitations and discuss how future RNA-based or isotopic tracing studies would be critical to confirm functional pathways and their relative contributions to N2O fluxes. Overall, the interpretation of the N2O data is overstated relative to the data. Statistical support for treatment effect is limited. Alternative explanations cannot be ruled out. The conclusion that processes governing N2O production are affected by function limitations is overstated and needs to be toned down.

We fully agree with the reviewer that transcriptomics and gross rate measurements have a high potential in further explaining the mechanisms underlying our observations, and we have clarified the limitations of our approach. When it comes to bacterial *amoA*, primer problems are unlikely as the primers we used have been used before and detect AOB, including in our previous study ref. [10]. In that study we also explored comammox *amoA* with both universal comammox primers and separate primer pairs targeting clades A and B, but this proved unsuccessful, therefore we did not follow up with comammox in the present study. In support of our decision, we note that Nitrospira, which includes comammox bacteria, were scarcely detected with our 16S primers, although we also acknowledge that highly divergent Nitrospira lineages may not be detected by our 16S primers and we can therefore not rule out their presence (L652-655). Contrary to the reviewer's understanding, our results do not show a dominance of AOA across many permafrost soils. Instead, AOA are absent from all permafrost soils unless inoculated with the Multifunctional inoculum, and were only detected in the active layer of the Cryoturbated soil prior to incubation. All permafrost soils,

however, appear hospitable to the establishment of AOA populations, and we now added discussion on this topic (L648-650).

Regarding limitations and possible speculative aspects when interpreting the N-cycling results, we now further emphasize precautions to reflect this (e.g. L637) and as mentioned in response to the previous comment have reworked the Discussion section on N_2O results over L626-666. However, we still suggest the functional limitations of ammonia oxidation, with consequences for N_2O production.

3. The strong increase in CH4 production following active layer inoculation of palsas is on the other side a robust and well-supported result. Saying here that there is functional limitation (without on-off switch) is reasonable. However, it bears me why functional genes related to CH4 production and oxidation were not studied to clearly link this process to changes and community structure and corroborate the main narrative of the MS? Despite extensive gene analysis, no methanogen-specific marker (e.g. mcrA) or taxon-level analysis were presented? The reference to Candids Mathanofloresn is good but relies on external studies rather than observations from the present one. Therefore, even here, the mechanistic interpretation of inoculation/microbial-driven methanogen introduction remains speculative and indirect.
We agree that additional mechanistic support would be a valuable addition. Consequently, we have now carried out qPCR assays of *mcrA* and *pmoA* genes in the anoxic incubation. *pmoA* genes were detected in all pre-incubation permafrost samples but, as expected, were not detected in any sample at the end of the anoxic incubation. Our results suggest that gammaproteobacterial methane oxidizers were present (but likely inactive in the anoxic conditions of the incubation since they are aerobic bacteria).
We detected *mcrA* in all samples during the incubation, but it was ~3 times lower at the end of the incubation than in pre-incubation permafrost. This suggests that *mcrA* genes were initially present, but were not activated or belonged to relic DNA or dead cells. We suspect that only a fraction of *mcrA* copies belong to active methanogens, and that while this active fraction may be higher in inoculated samples, the total number did not differ due to a concomitant increase in turnover of DNA. However, we cannot confirm this interpretation without an approach tailored to this question, such as transcriptomics or qSIP, which we therefore recommend for follow-up studies (L670-673).
We have added the new data to the manuscript and to Figure 4, and accordingly updated the Methods and Supplementary Table S2.

4. For CO2, a clear functional limitation on CO2 production was observed in all permafrost soils, ranging from 20-60% across all soils, though increase with native community was less clear. The explanation of why sometimes coalescence was successful and sometimes not is clear and convincing. since CO2 is the main GHG, this is an important result.
We agree that these results are important and intriguing. We now further emphasize them through the additional cross-dataset analyses we have added, including new Supplementary Figure S3.

5. The Cryoturbated permafrost soil consistently yielded low DNA quantities

and scarce functional gene detection, leading the authors to exclude it from some analyses. While transparency is good, discussion surrounding how this affects interpretation of functional limitations across soil types, and whether methodological constraints might bias conclusions, should be expanded. Are there alternative methods or deeper sequencing approaches that could have resolved these issues?
We assume the low quantity of DNA from this mineral permafrost soil stems from low biomass in the permafrost itself – presumably because the lack of sufficient organic matter did not allow a substantial microbial community to establish, with or without inoculum addition. Besides the observed low 16S rRNA gene copy number, DNA concentrations in the DNA extracts themselves were orders of magnitudes below those from the other permafrost soils, even in the pre-incubation samples. Deeper sequencing may thus not be very helpful here. Extracting DNA from larger amounts of soil (e.g. using PowerMax commercial kit, or saturated phosphate buffer extraction) would be an option, however we did not have enough material to proceed with this. We now acknowledge this and recommend increasing sample amounts for mineral permafrost soils (L517-519).

6. Given the importance of coalescence to biogeochemical outcomes, a more nuanced discussion of possible drivers (e.g., soil physicochemical properties, microbial traits, spatial-temporal dynamics), supported by references to recent literature, would be valuable. Additionally, the ecological relevance of the inoculation approach (how it relates to natural thaw conditions?) is worth elaborating.
We have now included soil moisture as a putative driver, although it did not show any consistent explanation in our data (see also our response to Reviewer #1 above). We added some discussion about the realism of our inoculation method in the context of recent literature, and suggestions on experimental approaches to progress on this topic (L689-703).

7. Incubations were conducted without plants, which likely influenced particularly N dynamics and N2O fluxes by removing plant N uptake. Strong NH4 immobilization as seen here as an indicator of N limitations of microbes is often the consequence. This limitation and its implications for extrapolating to field conditions should be discussed briefly.
Indeed, manipulating solely microbial community composition limits our ability to extrapolate to field conditions. We have now added some discussion of how future studies should integrate the role of plants and soil fauna and interactions between and among those and microorganisms to further improve our mechanistic understanding of thawing permafrost systems (L662-664 and L685-688).

8. The manuscript would benefit from explicitly reporting and discussing soil C/N ratios. These ratios are central to interpreting N availability, mineralization-immobilization balance, and N2O dynamics. Differences in C/N among soils may partly explain variation in nitrification, mineralization, N2O production and gene abundances.
We agree that differences in C:N ratios are central to different N and N_2O dynamics across soils, although they are unlikely to explain inoculum effects within a given soil. While we do not have EA data to present, ratios of DOC:TDN are very similar in the different permafrost soils (ranging from 7.8

to 9.0) and we did not see differences in TDC or TDN between treatments within a soil by the end of the incubation. We have now added permafrost and inoculum TDC:TDN ratios to Supplementary Table S1.

Specific comments:

Line 59: The transition to Yedoma is abrupt and overly specific. It would improve readability to first mention more common and widespread permafrost soil types (e.g., permafrost peatlands, uplands, cryoturbated soils) and frozen conditions, and then introduce Yedoma
We considered reorganizing this section, but felt that it would lose in clarity. As we need to introduce Yedoma deposits before explaining that functional limitations have only been evidenced in those deposits, discussing other permafrost types first would induce more back-and-forth. For the sake of conciseness, we retained the previous version of this paragraph.

Lines 87–89: The statement implying that changes in biogeochemical processes would largely depend on changes in microbial community composition is too strong. It I also contradicting the functional limitation hypothesis of a given community of the current study. Functional limitations can also be alleviated by activation of existing microbial populations via substrates, exudates, or environmental modifications introduced by interacting communities, without major compositional turnover.
We agree, and while we did measure a variety of physical and chemical parameters, we cannot ensure only microbial community composition was manipulated in our approach. We have now removed this statement which did not fully reflect our intended meaning, see also our response to Reviewer #2 above.

Lines 105–106: The third statement reads more like an interpretation than a testable hypothesis. It would be clearer to move it to the discussion and keep the hypothesis section focused on directly testable predictions about functional limitation and restoration.
We have now reworked the hypotheses following this input and that from Reviewer #2. This statement is now decoupled from the hypotheses and instead presented as an expectation of the mechanisms underlying observations related to hypotheses 1) and 2) (L108-115).

Lines 149–150: Only the palsa mire was incubated under anaerobic conditions. The authors should briefly explain this choice (e.g., future subsidence and waterlogging are particularly relevant for palsa systems).
We now justify the choice of the palsa mire system for exploratory work on anaerobic processes at L158-159.

Line 212: Consider revising to: “Production and/or consumption rates of CH4 and N2O…”
We have now revised this to “*net production rates*” (L229), because our experimental design did not allow to specifically measure consumption rates or distinguish between production and consumption. See also our response to the next comment.

Lines 311–312: Was the cooling potential associated with possible N2O uptake considered? Waterlogged anaerobic peat soils can consume atmospheric N2O, which could offset warming from CO2 and CH4. Even if N2O was below detection, assuming upper and lower bounds (e.g., detection limit vs. zero) could help frame the potential cooling effect.
While we are aware of the importance of N_2O sink potential post-thaw (e.g. Voigt *et al.* 2017), here we could not consider N_2O uptake dynamics and subsequent cooling potential, as the headspace gases (Linde Gas HiQ synthetic air 5.0 in the oxic incubation, Strandmöllen Lab Line N_2 5.0 in the anoxic incubation) did not contain N_2O and detectable amounts were not observed in the anoxic incubation. For N_2O uptake to happen, say from detection limit to zero, this N_2O would have to be first produced by the microorganisms in the incubation, yielding at most a net null effect and preventing uptake estimates.
We now mention this caveat in discussion (L636-638) and recommend future studies include detectable concentrations of N_2O and CH_4 in headspace gas, to allow for measuring cooling effects due to N_2O uptake or methanotrophy (L661-664).

Lines 323–324: Please clarify why cumulative N2O budgets could not be calculated (e.g., limited sampling frequency).
We now clarified at L354-359 how the sampling frequency and regular flushing of the headspace for CO_2 concentration analyses prevented us from calculating full N_2O budgets, see also our response to Reviewer #2 above.

Figure 2: Indicating statistically significant differences relative to controls directly in the figure would greatly aid reader.
The significance letters of the post-hoc tests within each panel consider comparisons across all treatments, and thus allow at a glance to see which treatments differ from the controls. We are willing to revise this at the editor’s discretion.

Line 406: If CH4 production increased fourfold, describing it as “limited” may be misleading; consider rephrasing.
We have rephrased this (L443-444).

Line 455: Please specify clearly whether a lower nir/nos ratio is meant.
We now clarify the direction of this difference (L514).

Line 573: The text should also acknowledge the strong decrease observed in N2O.
As discussed in our response above, the headspace was flushed between each measurement with gas not containing N_2O, thus we did not witness a decrease in N_2O. However, we now mention the relevance of N_2O uptake for future studies at 636-638 & 661-664.

Line 574: I strongly sugget to replace “carbon-climate feedback” with “permafrost-climate feedback,” since both carbon and nitrogen processes were studied.
This is a very good point, thank you for the suggestion, we have now rephrased this accordingly (L711).

RESPONSE TO REVIEWERS' COMMENTS

Reviewer #1 (Remarks to the Author):

Reviewer report

The authors have provided a carefully revised version of this interesting manuscript, where both reviewers' concerns are in my opinion properly addressed. Additional discussion, analysis, and many small but important clarifying details have been added. Limitations of the study are discussed as well as the broader implications with some suggestions for future studies.
We thank the reviewer for their appreciation of our revisions.

Some parts of the new text (indicated below with line numbers) appear slightly raw compared to the rest of the text and would benefit from rephrasing. The article can be published after a minor revision related to those few points.

line 33: Based on N2O section starting on line 413, the increases in N2O were not statistically significant. Thus, this seems like an overstatement and some rephrasing is needed.
We consider that detecting N_2O in higher concentrations in inoculated soils (14 occurrences), but not in the controls (2 occurrences), suggests an increase, which could not be statistically tested in most cases due to too few measurements above detection limit. Retaining measured values below detection limit, or setting all values below detection limit to half the detection limit would lead to detecting significant increases from Multifunctional inoculum in Palsa-PF (day 17) and Tussock-PF (day 187). These approaches either rely on data which is not reliably quantitative (below detection limit) or produce an artificially low variation (set value), we therefore deemed those inelegant and they were not included in the manuscript. Although not supported by robust statistics, we do consider our results as evidence of increases.
We have rephrased the statement in the abstract from "increase" to "onset" to better reflect the lack of statistical support while still acknowledging the findings (L31).

lines 376-381: This is a nice analysis, but I find the wording here unnecessarily complicated. Would be great if you could provide a concise plain language explanation of the aims of this analysis. Was the aim to investigate which microbial classes were most important in driving the treatment differences in cumulative CO2 production?
We have now added a short plain language summary of the approach to the beginning of this paragraph (L681-684), and simplified the phrasing throughout this paragraph. We think the text now better describes the aim which was to look for the best descriptors of differences in CO_2 production.

line 386: Consider using a full stop between '58 data points' and 'we' instead of a comma.
Done.

line 486-> Based on this analysis, is it possible to say which microbial classes had a positive effect and which negative on the CO2 production? Or, do you mention only those classes which had a positive effect? Please clarify this.
All the significant correlations we identified were positive correlations, we now clarify this at L197-198.

line 487: Soil chemistry is mentioned here, but no results regarding soil chemistry are reported in this section. It remains unclear if and how they were used. Please clarify.
We now rephrased (L192-195) to clarify that both soil chemistry and bacterial classes were included as variables, but only bacterial classes were retained by the analysis as being important descriptors of the variability in CO_2 production.

line 494: Present verb (correlate) within a text written in past tense.
Fixed.

lines 499-502: A long and complicated sentence hard to understand; please revise for better clarity and text flow.
We have now rephrased this sentence (L202-206).

lines 622-625: Some rephrasing needed. The sentence is long and complicated and the meaning remains unclear.
We have now clarified the confounding factors of inoculation treatment and increase in CO_2 production (L324-327).

lines 723-724: Neither of these two links seem to work?
The DOI for the revision of the code and data was not minted at the time, this is now solved.

Reviewer #2 (Remarks to the Author):

The authors thoroughly revised the manuscript and most of my initial concerns are considered in the revised manuscript. I have only some minor comments/suggestions.
We thank the reviewer for their appreciation of our revisions.

However, some of my previous concerns remain. Firstly, rather than presenting a novel concept, the interesting data in this paper confirms the results of previous papers. Secondly, some of the data is interpreted overly speculatively, particularly with regard to the nitrogen cycle.
We addressed the concerns regarding novelty and further clarified and refrained from the speculative nature of parts of the nitrogen cycling discussion in the previous revision. Since these concerns were not raised by the other reviewers and the editor, we respectfully acknowledge the difference in perspective.

Specific comments:
L104f: unclear what positive and negative controls mean
Thank you for spotting this oversight left from renaming the treatments in the previous round. We have now clarified this at L101.

L173: ‘increase’
Fixed.

L217ff: Flushing does not bring CO2 concentrations to zero, at least as long as there is still CO2 dissolved in the sample water. Please rephrase. Ignoring the remaining CO2 in the flasks would result in an overestimation of gas production, ignoring the dissolved inorganic carbon in the water would cause an underestimation of the gas production rates. Both should be mentioned. Not very clear to me, why you choose to ignore DIC in the incubations, you should have all the data needed to account for it.
In our tests, the CO_2 concentration in the jars after flushing was hardly detectable by our instrument, no more than 10-20ppm v:v, in contrast to several thousands ppm at measurement time. We did not directly measure DIC at the same temporal resolution as CO_2. At this point we could only estimate DIC based on pH measured at the beginning and end of the incubation, which would not allow to account for it throughout the 21 CO_2 measurement times. We have now added clarifications regarding flushing and DIC outgassing (L519-520 and 527-530), clarifying that while it would not affect our comparisons between the different treatments, it should be considered when trying to derive quantitative insights from our data, such as for upscaling exercises.

L560ff: I was not able to extract the age data from the given reference since it uses these data to determine carbon accumulation rates without presenting ages in the main text. Please give the age range of the non-Yedoma permafrost as far as they are available to substantiate this conclusion.
In that reference ^{210}Pb chronology was only applied to the top 20cm of the active layer, and ^{14}C throughout the whole cores. The oldest ^{14}C-derived ages from this reference reach 9600 cal.BP (Supplementary Figure S6 in Olid et al. 2020 doi:10.1111/gcb.15283), which closely matches the estimates of ice sheet retreat for the region (10 ka, Hughes et al. 2016 doi:10.1111/bor.12142). The Tussock-PF permafrost is at the limit of the estimated Yedoma extent and it is uncertain whether it is underlain at depth by Yedoma or lies on more recent glacial deposits. Nearby sites underlain by Yedoma yield ^{14}C ages of ~8000 cal.BP for the depth we sampled (Kanevskyi et al., 2011, doi:10.1016/j.yqres.2010.12.003), while Itkillik II glaciation would push it to about 12 ka. We now have clarified the date and added a reference to Kanevskyi et al. 2011 (L266).

Reviewer #3 (Remarks to the Author):

The authors have done an excellent job revising the manuscript in response to all reviewer comments. My own comments and concerns have been thoroughly addressed and satisfactorily resolved. In addition, the authors conducted further molecular analyses, which have strengthened the study and improved the overall quality of the manuscript.

Overall, the revisions have made the manuscript substantially stronger, and I have no further comments. I believe this work represents a valuable contribution to permafrost science and will be of broad interest to the community. I therefore support publication.
We thank the reviewer for their appreciation of our revisions.
